# Incorporation of Noncanonical Amino Acids into Rosetta and Use in Computational Protein-Peptide Interface Design

**DOI:** 10.1371/journal.pone.0032637

**Published:** 2012-03-14

**Authors:** P. Douglas Renfrew, Eun Jung Choi, Richard Bonneau, Brian Kuhlman

**Affiliations:** 1 Department of Biology, Center for Genomics and Systems Biology, New York University, New York, New York, United States of America; 2 Courant Institute for Mathematical Sciences, Department of Computer Science, New York, New York, United States of America; 3 Department of Biochemistry and Biophysics, University of North Carolina at Chapel Hill, Chapel Hill, North Carolina, United States of America; University of South Florida College of Medicine, United States of America

## Abstract

Noncanonical amino acids (NCAAs) can be used in a variety of protein design contexts. For example, they can be used in place of the canonical amino acids (CAAs) to improve the biophysical properties of peptides that target protein interfaces. We describe the incorporation of 114 NCAAs into the protein-modeling suite Rosetta. We describe our methods for building backbone dependent rotamer libraries and the parameterization and construction of a scoring function that can be used to score NCAA containing peptides and proteins. We validate these additions to Rosetta and our NCAA-rotamer libraries by showing that we can improve the binding of a calpastatin derived peptides to calpain-1 by substituting NCAAs for native amino acids using Rosetta. Rosetta (executables and source), auxiliary scripts and code, and documentation can be found at (http://www.rosettacommons.org/).

## Introduction

From the original full automated sequence design of Dahiyat and Mayo [1] to recently designed enzymes [2], [3] and influenza binders [4], computational protein design has become an increasingly powerful tool for protein engineers. In most cases, computational design programs have been constructed to primarily work with the twenty canonical amino acids (CAAs) found in humans. The ability to apply the tools and techniques, developed to design proteins, to other protein-like polymers could allow for the creation of new therapeutics and biological tools. A logical step towards this goal is the incorporation of noncanonical side chains (NCAA) in to computational protein design software. The use of NCAAs in protein design programs has advantages both biologically and computationally.

Biochemists and biologists have already demonstrated the utility of NCAA derived polymers, and CAA-NCAA hybrids. For example, changing the chirality of a protein by constructing it entirely out of D-enantiomers has been shown to provide proteolytic resistance [5], an issue which has been a problem for protein therapeutics [6]. Protein stability has been increased without significantly disturbing protein structure by replacing common hydrophobic residues with fluorinated derivatives [7]. Numerous protein crystal structures have be solved with the aid of selino-methionine phasing [8]. Chemically restrained amino acids that have particular ϕ and ψ angle preferences have been used to promote helix formation [9]. Modified residues have been shown to improve enzyme kinetics and expand endogenous function [10]. These results have been obtained without extensive computational modeling and were probably limited in the scope of what they could design by similarity to the CAAs.

The use of NCAAs in design will dramatically increase the number of sequences and side chain conformations that can be sampled during a design simulation. The additional diversity may allow for the creation of more tightly packed hydrophobic cores and new hydrogen bond networks. Additionally, incorporating amino acids with intrinsic torsional constraints can lower the entropic cost for assuming a folded or bound state.

The term “nonnatural amino acid” is often used to denote NCAAs, but the use of the term “nonnatural” is perhaps a misnomer in this context, as amino acids that differ from the canonical twenty are frequently found in nature. The most common NCAAs are residues with pre-/co-/post-translational modifications that provide them with additional functionality [11]–[13]. Eukaryotes, prokaryotes, and archea have all been found to have selenocysteine residues which are genetically encoded indirectly by overloading the UGA stop codon in conjunction with a selenocysteine insertion sequence element [14]. Additionally some methanogenic archaea genetically encode pyrrolysine indirectly by overloading the UAG stop codon in conjunction with a pyrrolysine insertion sequence element [15].

Computational protein design programs typically contain two major components: an energy or scoring function to evaluate how well a particular amino acid sequence fits a given scaffold and a search function that samples sequences as well as backbone and side chain conformations. Energy functions for protein-design often contain a combination of physically-based and knowledge-based terms [16]. Knowledge-based terms are generated from naturally occurring protein structures, and are generally based on the probability of observing a particular structural feature in a set of structures. Knowledge-based potentials are often information rich and quick to evaluate, but care must be taken to avoid double counting between components of the energy function [17]. Knowledge-based potentials that function at the level of amino acid identity can not be built for NCAAs because there are not enough structures in the Protein Data Bank that contain NCAAs to derive meaningful statistics. To enable the modeling of NCAAs in the program Rosetta [18], we have removed knowledge-based terms incompatible with NCAAs and replaced them with more general functional forms typically found in molecular mechanics energy functions such as Amber and CHARMM.

By using Rosetta for design with NCAAs, we gain access to a wide variety of kinematic and optimization based methods for exploring backbone and side chain configurations. Conformational searches of backbone degrees of freedom are typically performed using small perturbations to the backbone dihedral angles, fragment insertions, backrub movements, or using more sophisticated procedures like using robotic arm motion planning inspired loop-closure algorithms [19]–[21]. Conformational searches of side chain degrees of freedom are performed in a discrete space of high probability side chain conformations typically encoded as backbone-dependant rotamer libraries. Rotamer libraries are lists of commonly seen side chain dihedral angles [22] accompanied by the probability of observing each rotamer in naturally occurring proteins. In Rosetta, the side chain coordinates are constructed using dihedral angles from the rotamer library and idealized bond lengths, bond angles, and non-χ dihedrals [23].Amino acid rotamers are not observed with equal frequency in large databases of experimental structures. The probability of seeing a given rotamer given its local structure context can be used to compute a pseudo-energy that represents the internal energy of the amino acid. Rosetta (and many other related methods) assumes a Boltzmann distribution and uses the log of the probability of seeing a given rotamer with particular ϕ and ψ backbone dihedral angles to estimate rotamer energy as shown below.
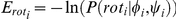



Where *E_roti_* is the energy of rotamer *i*, ϕ_i_ and ψ_i_ are the ϕ and ψ backbone dihedral angles at position *i*, and *P_roti_* is the probability of seeing rotamer *i* when the backbone dihedral are ϕ_i_ and ψ_i_. The probabilities in this equation come from the Dunbrack rotamer library [24]. The frequency of rotamers also provides a way of limiting the conformational search to the statistically most likely conformation. Building rotamer libraries for NCAAs is a prerequisite to using these NCAAs in Rosetta, or any Rosetta-like design procedure. As with the knowledge-based potentials, the use of statistically derived rotamers libraries to provide common side chain coordinates is not possible for NCAAs as there are not enough solved structures to compute accurate statistics. We have thus developed a method to create rotamer libraries for NCAAs that can reproduce the rotamers seen in CAA. The modifications we have made to the energy function that allow for the scoring of NCAAs and the ability to create rotamers libraries allows us to use NCAAs in the computational protein design program Rosetta. We created condition dependent rotamer libraries for 114 NCAAs and have incorporated these NCAAs into Rosetta. These NCAA rotamer libraries, the code used to construct new rotamer libraries and the modified version of Rosetta corresponding to this work are freely available to academic groups at: http://www.rosettacommons.org.Archives that include everything required to reproduce this work (scripts, input data, example runs and directory structure, and tutorials) are included in a single archive included as Supporting Information S2.

**Figure 1:**
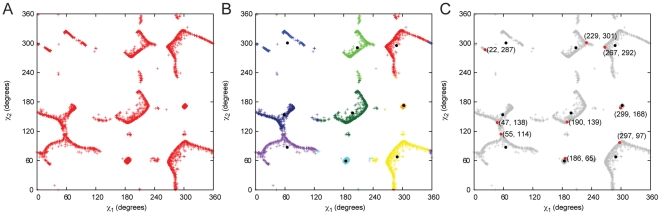
The structure of calpain and calpastatin. (A)The calpain-1 DI-DVI (green) with calpain-4 DVI (cyan) with a calpastatin subdomains A,B, and C (magenta). Dashed lines are where there was no density in the crystal structure for calpastatin. (B) Enlarged view of the interaction between subdomain C of calpastatin and DVI of calpain-4 indicated in A by black square.

Here, we used our modified version of Rosetta to increase the binding affinity of subdomain C of the calpastatin peptide for domain DVI of the calcium dependant cysteine protease calpain. Calpain, is involved in many important cellular pathways [25]. The number of proteins targeted for proteolysis by calpain implicates it in a variety of diseases [26]–[28] implying that inhibitors of calpain could be of potential therapeutic use. Structural characterization of the calpain/calpastatin interface has shown that calpastatin subdomain C forms an amphipathic α-helix that binds to a hydrophobic patch on the DIV domain of calpain (figure 1) [29]–[31]. We have computationally redesigned positions on the interface between calpastatin and calpain by allowing NCAAs at the calpastatin positions as a first test of our integration of NCAAs into Rosetta, and show that we can improve binding of a calpastatin-derived peptide with calpain.

## Materials and Methods

### Modification of the Rosetta Energy Function

The Rosetta energy function is a linear sum of individually weighted terms as shown below and in Rohl *et al.* [19]. It contains a physically-based inter-residue Lennard-Jones term split into repulsive and attractive components (*E_inter_*__*rep*_ and *E_inter_atr_*) [32], a implicit solvation term implemented as described by Lazarids and Karplus (*E_solvation_*) [33], knowledge-based reside pair electrostatics term (*E_pair_*), orientation dependent hydrogen bonding term (*E_sc/bb hb_*, *E_bb/bb hb_* and *E_sc/sc hb_*) [34], a knowledge-based term that measures the internal energy of an amino acid based on probabilities from rotamer libraries (the rotamer internal energy term, *E_dunbrack_*), a knowledge-based term that measures Ramachandrin backbone torsion preferences of a position (the rama term, *E_rama_*), and a reference energy term that represents the energy of the unfolded state of a protein (*E_ref_*) [35], [36].
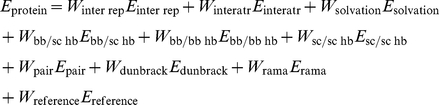



The inter-residue attractive and repulsive terms are physically based and can be applied to NCAAs. The solvation term and the hydrogen bonding terms are evaluated on atom-atom pairs and thus applicable without modification to NCAAs. The rotamer internal energy term, the rama term and the pair term are knowledge-based, conditioned on residue identity and are not compatible with NCAAs. To replace the internal energy term and the rama term we have implemented a intra-residue molecular mechanics Lennard-Jones term and a matching molecular mechanics torsion term, both described below. The reference energy term has been replaced with a term that uses an explicit unfolded state model described below. The pair electrostatic term has been omitted. The modified energy function used for scoring CAAs and NCAAs is shown below.
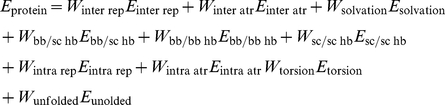



In contrast to molecular mechanics programs which often views proteins as a fixed set of atoms, bonds, bond angles, and dihedral angles, the energy functions used by computational protein design programs must be able to rapidly handle changes to the protein amino acid sequence. This is achieved by decomposing the energy function in to terms that can be evaluated between pairs of prospective amino acid rotamers. Here we denote energy terms that that can be evaluated without information about the surrounding rotamers “one-body” terms (*e.g. E_dunbrack_*), while energy terms that require information about the surrounding rotamers are referred to as “two-body” terms (*e.g. E_intra rep_*). The combination of the molecular mechanics torsion and intra-residue Lennard-Jones terms can accurately describe the rotation about a bond in a protein design scenario using fixed bond lengths and angles [37].

Instead of using a molecular mechanics potential to model side chain torsion energies, we considered using quantum mechanics (QM) single point energy calculations to determine rotamer preferences. With this strategy, the alternate rotamers of a side chain are modeled in the context of a dipeptide and the internal energy of each side chain conformation is calculated with high level QM simulations. The QM derived internal energies are then assigned to the appropriate rotamers while performing full protein design simulations. Previously we showed that this approach works well for valine, leucine and isoleucine, and that in some scenarios the QM derived energies outperformed molecular mechanics energies in side chain prediction tests [17].We choose not to use this approach for the NCAA side chains because it would require a very large amount of computer time (>100 million CPU hours) for the full set of NCAA rotamers that we are considering and because our QM-based approach does not work well with polar side chains. The QM simulations are performed in a vacuum and therefore polar side chains typically form strong interactions with their own backbone, interactions that would be partially shielded in a solvated environment.

### Implementation of the CHARMM Torsion and Lennard-Jones Potentials in Rosetta

We have implemented a molecular mechanics torsion term of the form shown below using the CHARMM27 parameter set [38].
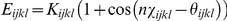



Where the four atoms that comprise the dihedral angle are indicated *i*, *j*, *k*, and *l*, *K* is a constant, *n* is the multiplicity (e.g. n = 2 for sp2, n = 3 for sp3), χ is the angle of the dihedral, and θ is the offset. Note that a single chemical bond may have more than one of these terms such that the sum is expressed as a Fourier series. The torsion term is evaluated for all sets for 4 connected atoms in a protein.

We have matched the molecular mechanics torsion term with a matching molecular mechanics Lennard-Jones term of the form shown below also using the CHARMM27 parameter set [38].
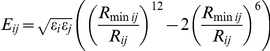



Where for two atoms of types *i* and *j*, 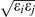 is the well depth, 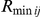 is the distance at which atoms of type *i* and *j* are at an energetic minimum, and 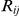 is the distance between the two atoms. The term is evaluated between all pairs of atoms within an amino acid rotamer that are separated by three or more chemical bonds.

### Estimating an Explicit Unfolded Energy Term for NCAAs

The reference energy term in Rosetta represents the unfolded energy of the protein; this term corrects for the relative difficulty of packing large side chains and side chains with large numbers of rotamers, and has been shown to be essential for native amino acid recovery performance [1], [2], [3] (a primary test of any design procedure). The individual values for each CAA reference value (one per amino acid type) are independent degrees of freedom that represent the average value of that scoring term in the unfolded state; weight fitting for the Rosetta-design reference energy is done using a training set of proteins that contain only CAAs and the reference energy is therefore not applicable to NCAAs. We have implemented a term to replace the reference energy term that uses an explicit unfolded state model and is compatible with both CAAs and NCAAs. To estimate the unfolded energy of an amino acid we first use fragments of protein structures to create a random backbone ensemble, and then repack the NCAA in question into each structure in this ensemble. To create an “unfolded” backbone ensemble we break a set of ∼1500 high resolution, low redundancy, protein structures into randomly chosen 5-mer fragments. The list of structures was generated from the a subset of the pdb culled with PISCES to remove redundancy and low resolution structures [39]. The central residue of each fragment in this ensemble is mutated, and the full five-mer is allowed to repack. The unweighted energies of each energy term for each central residue ensemble are averaged and stored. When scoring a particular position, the averaged unweighted residue-based energies are multiplied by the weight from the respective energy term as shown in below. 
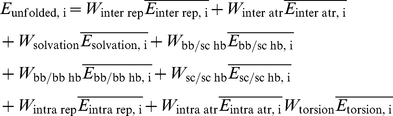



Where 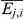 is the average unweighted energy for energy term *j* and residue type *i*, and the weights are the identical weights used for each energy component in the Rosetta energy function.

### Determining weights for individual energy terms

The Rosetta energy function is the sum of individual weighted energy terms as show above. Substantial changes to the terms in the energy function require a re-optimization of the weights on the individual terms. The weights are trained to maximize the probability of seeing the native amino acid at each position in a set of high-resolution protein structures during a complete sequence redesign. The weights on certain terms can be kept fixed or allowed to change. The fitting is done by calculating the unweighted energies for all rotamers at all positions in all of the structures and then optimizing the weights on the free terms using a combination of particle swarm optimization [40] and quasi-Newton minimization [41] to maximize a fitness function. The fitness function used is designed to maximize the probability that the native amino acid (in the context of a high resolution crystal structure) is scored with a lower energy than all other amino acids and is shown below. Lastly, the new set of weights is used to redesign the set of training proteins and native sequence recovery is tested [42]. If the sequence recovery increases, the new set of weights is accepted. If the sequence recovery decreases the new weight set is averaged with the previous weight set. These three steps are repeated 10 times. The fitness function, *F*, which is maximized during the optimization, is shown below.
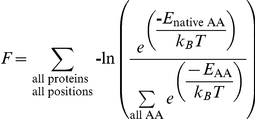



Where *E* is the Rosetta energy, *kB* is the Boltzmann constant, and *T* is the temperature.

### Rotamer Library Creation

**Figure 2:**
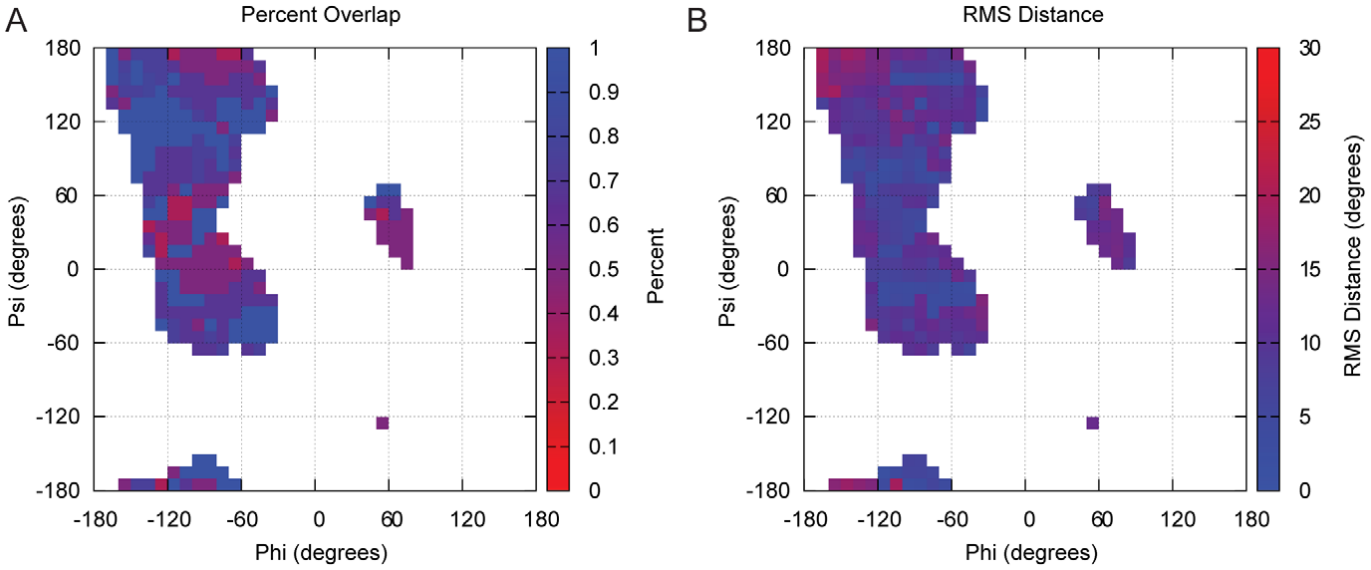
Rotamer library creation protocol. The steps of the MakeRotLib protocol are shown for leucine with ϕ = −60 and ψ = −40. For a given ϕ and ψ a set of leucine dipeptides is created with side chain angles initially set to all χ_1_ and χ_2_ values in 5 degree intervals. (A) Each dipeptide is minimized keeping the ϕ and ψ fixed okay each trip up to. (B) Side-chain dihedral values are clustered all members of each cluster are indicated using separate colors. (C) Black points indicate centroids of the clusters depicted in (B), Red dots indicate the lowest energy cluster member. The lowest energy set of side chain dihedrals in each cluster (red points) comprise the rotamer library for leucine.

We have developed a protocol, called MakeRotLib, which can create backbone dependent amino acid rotamer libraries for both CAAs and NCAAs as shown in figure 2. The rotamer calculations are performed using an amino acid dipeptide model system, a single residue with an acetylated N-terminus and an N-methylated C-terminus. The dipeptide system mimics all ϕ- and ψ-dependent side-chain interactions with the surrounding protein backbone. ϕ and ψ backbone dihedrals are sampled in 10 degree intervals creating 1296 ϕ/ψ bins. For each ϕ/ψ bin, a set of amino acid dipeptides are created with χ dihedrals sampled in varying size intervals depending on the number of χ angles, the composition of the side chain (*e.g.* 1 χ angle for Val, 2 χ angles for Phe), and the expected number of rotamers (this can be a function of the number of dihedrals, but is a user defined parameter).

Each dipeptide (built to test/sample a given χ, ϕ and ψ) is minimized with 25 steps of linear-gradient minimization to the closest local minimum with ϕ, ψ and non-χ side chain dihedrals kept fixed during minimization. Linear minimization was chosen over other forms of minimization because it explores the nearest local minimum (the correct behavior, as we wish to characterize many separate minima as distinct rotamers). The rotamers of amino acids side chain are simply the local minimum in the side chain energy landscape. The set of minimized side chain dihedral angles for leucine with α-helical backbone dihedrals (ϕ = −60 and ψ = −40), with both side chain χ angles starting values sampled at 5 degree intervals, is shown in figure 2A.

Following minimization, the sets of minimized side chain dihedral angles are clustered using a K-means clustering algorithm to reduce the explored minima to a smaller set of distinct rotamers. The K-means algorithm works by first calculating the root mean squared distance between each set of side chain dihedral angles and each member of a set of cluster centroids. Each set of side chain dihedrals is assigned to the closest cluster centroid. Second, the cluster centroids are recalculated to be the geometric mean of the members of that cluster following reassignment at the prior iteration. The algorithm iterates between these two steps until no side chain dihedral sets change clusters or 500 iterations. The minimized angles are shown for leucine in figure 2B.

We do not predefine limits or bins in which rotamers can exist. A major drawback of our approach is that it requires the number of clusters and an estimate of the starting positions of the cluster centroids to be set before hand. The number of rotamer bins for each amino acid and the starting values of the cluster centroid positions are determined using test runs and expected results based on previous rotamer libraries. The set of side chain dihedral angles to be used as the angles for each rotamer is the lowest energy set of angles in each cluster after the iterative clustering procedure. The final rotamers for leucine with α-helical backbone dihedrals (ϕ = −60 and ψ = −40) are shown in figure 2C. In order to properly interpolate between rotamer bins in the Rosetta framework and to more directly conform to the format of the commonly used Dunbrack library the number of rotamer bins for each ϕ/ψ bin must be equal, requiring us to populate all rotamer dihedral bins (including high energy, rare configurations). The Dunbrack rotamer library provides standard deviations that describe the width of rotamer bins. Rosetta, and other codes, use these standard deviations to calculate off-rotamer side chain conformations that increase the number of rotamers sampled. To calculate standard deviations for NCAAs needed for Rosetta (and other design programs) we sample around each side chain χ angle and report angle deviations consistent with estimated energy increases of 0.5 kcal/mol.

Rosetta makes use of the probabilities of a given rotamer listed in the Dunbrack rotamer library for determining the internal energy but also as a way to eliminate high-energy rotamers prior to full energy function evaluation. Rosetta only uses the top 95% of rotamers, ranked by probability, for each ϕ/ψ bin during side chain optimization. The rotamer libraries generated here are not used for energy evaluation but only as starting points for the side chain packing. However the removal of high-energy rotamers speeds up side chain optimization. We therefore convert the energies to probabilities for this purpose using:
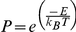



Where *P* is the probability, *E* is the energy of the rotamer, and *k_B_T* is the Boltzmann constant. Probabilities are normalized to sum to 100% for each ϕ/ψ bin.

### Selection of NCAAs for initial Rotamer Library

NCAAs were chosen: 1) based on commercial availability, 2) to have good model-ability using the existing CHARMM torsion and Lennard-Jones parameters, and 3) to have four or fewer heavy atom side chain χ angles. Some conformers of NCAAs are difficult to model using rotamer libraries because they involve coordinated movements of multiple torsion angles (*e.g.* the transition between the “chair” and “boat” cyclohexo ring conformers). In these cases the different conformers were modeled as independent residue types. For a full list of the NCAAs added see the Supporting Information S1.

### Comparison to Knowledge-Based Rotamer Libraries

**Figure 3:**
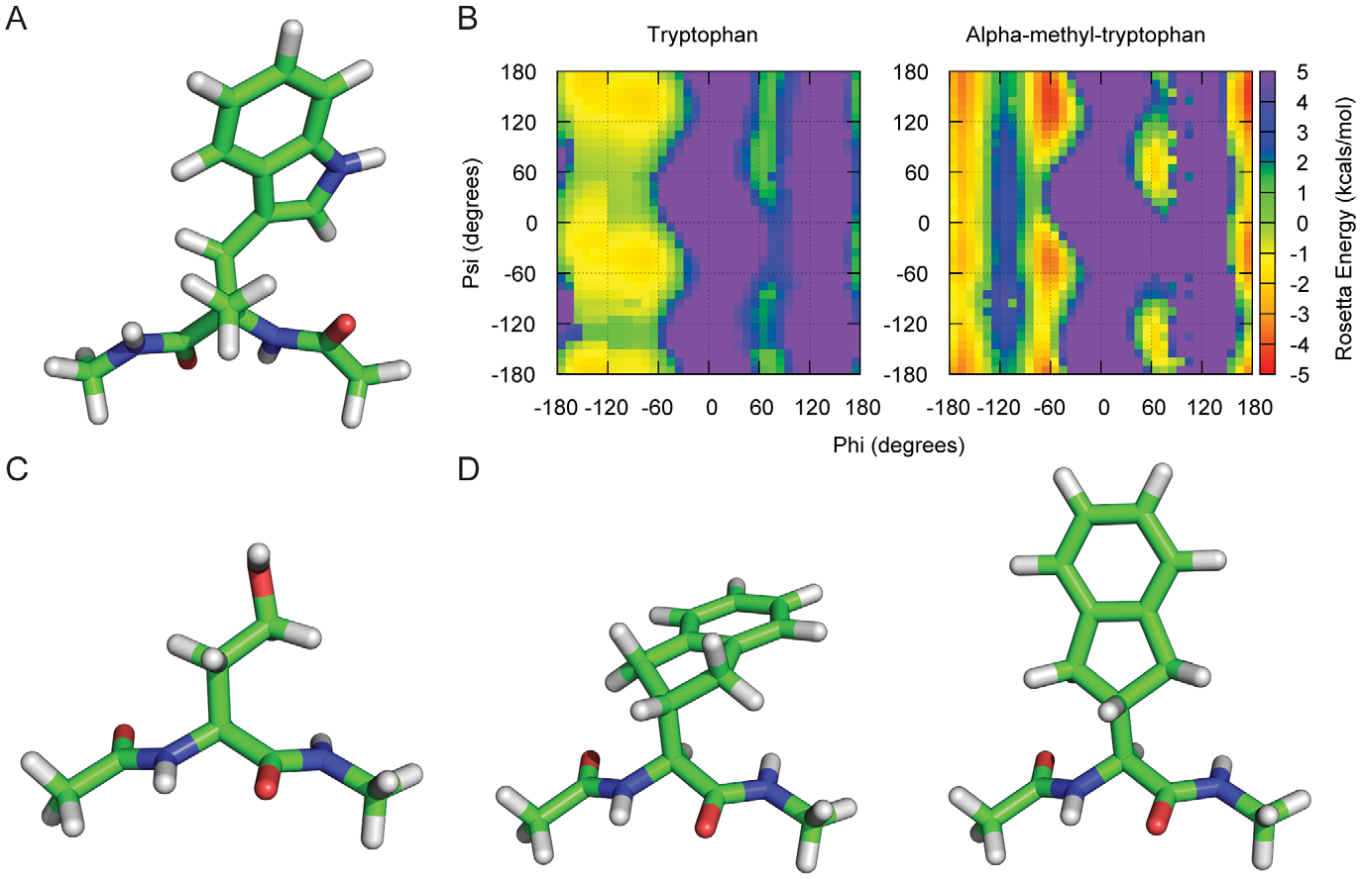
Percent overlap and RMS distance for the top 95% of rotamers between the Dunbrack rotamer library and the rotamer predicted by the MakeRotLib protocol for leucine. (A,B), asparagine (C,D), and phenylalanine (E,F). For each ϕ/ψ bin with more than 10 observations in the Dunbrack rotamer library, the percent overlap between the rotamer bins that comprise the top 95% of rotamer bins is calculated. For each pair of rotamer bins that overlap the root mean square distance in degrees is calculated. See methods for additional details on creation and results for details on analysis. A full description of how overlap and RMSD are calculated, given two rotamer sets for a given residue, are provided in the methods section.

To test the MakeRotLib we compared the overlap in rotamer identity between the top 95% of rotamers predicted by the MakeRotLib protocol and the top 95% of Dunbrack rotamers for each ϕ/ψ bin and for all amino acids except alanine, gylcine and proline (this is the relevant comparison as Rosetta uses only the top 95% of rotamers given by the Dunbrack rotamer library for each ϕ/ψ bin). For each ϕ/ψ bin where the Dunbrack rotamer library has more than 10 observations for a particular amino acid, we compare the percent overlap between the identities of the rotamers bins. Percent overlap is calculated for each ϕ/ψ bin by first reading rotamers in order from most probable to least probable from both Dunbrack and MakeRotLib rotamer libraries until the summed probabilities of those rotamers is > = 95% individually. The fraction of rotamers in the MakeRotLib set that have the same rotamer bin as the rotamers in the Dunbrack set is the percent overlap. Rotamer bins are considered overlapping if the root mean squared (RMS) distance between side chain dihedral angles, calculated by taking the square root of the sum of the squared differences between the individual χ angles, is less than 30 degrees. Comparisons of the percent overlap and RMS deviations for matching rotamer bins for Leu, Asn, and Phe rotamer libraries are discussed below and shown in figure 3 (see Supporting Information S1 for all other CAA comparisons).

### CAA Sequence and Rotamer Recovery Benchmarks

The modified energy function was tested using its ability to score CAAs using two benchmarks: a rotamer recovery benchmark, and a sequence recovery benchmark. For both benchmarks the “pack rotamers” (*vida infra*) side chain optimization procedure is first performed on the set of high-resolution protein structures we use to benchmark our procedure. In the rotamer recovery benchmark, the rotamers used are limited to the rotamers of the native amino acid present at each position in each benchmark structure and the percent of native rotamer recovered is recorded. In the sequence recovery benchmark, the rotamers of all CAAs are allowed at each position and the sequence identity is recorded.

### Peptide-Protein Interface Design Protocol

The design protocol designs a peptide-protein interface, starting from an experimental structure of the peptide-protein interface, allowing only peptide side chains to change sequence. The protocol iterates between exploring backbone conformations and a design phase that searches for low energy sequences to fit the current backbone. The backbone phase has 2 parts: a backbone perturbation and a round of “rotamer trials.” First one of the following backbone perturbations is performed on the peptide/protein complex: a “small” move, where ϕ or ψ of a randomly chosen residue on the peptide is rotated by up to 3 degrees, a “shear” move, where the ϕ of a random residue on the peptide is rotated up to 3 degrees and the ψ of the preceding residue is rotated by an equal amount in the opposite direction, a ridged-body translation of the peptide in the binding pocket, or a ridged-body rotation of the peptide in the binding pocket. Each one of these perturbations is followed by a round of rotamer trials where for each residue that increased in energy during the backbone movement, the best rotamer is chosen given the current context; positions in the peptide are optimized in random order.

The design phase consists of two parts: a round of the “pack rotamers” routine followed by gradient minimization. The “pack rotamers” routine optimizes rotamers given a set of residues using a simulated annealing Monte Carlo/Metropolis search. The routine randomly chooses a single rotamer to replace (rotamers can be from different amino acid types than the current amino acid at the position) and determines the energy of the complex if the change is made; changes are accepted based on the Metropolis criterion. Following the pack rotamers routine, gradient-based minimization of the complex is performed. Both backbone and side chain dihedrals of the peptide and side chain dihedrals of the protein as well as the distance between the peptide and the protein are allowed as degrees of freedom. All residue side chains on the peptide are allowed to repack but only protein residues within 6 angstroms of the peptide are repacked. To generate a single design we performed 50 iterations of 100 cycles of the perturbation phase followed by 1 cycle of the design phase. The protocol is not designed to find a new binding mode but to allow enough flexibility in the interface to allow the possible incorporation of NCAAs.

All designs were created using the 2.0 angstrom resolution crystal structure of a calpain-4 domain DVI bound to a 19mer peptide of calpastatin comprising subdomain C of the first inhibitory repeat (PDB code 1NX1) [10]. Only 11 residues of the peptide were resolved in the crystal structure (positions 601–611). The structure contains a homodimer of DVI in the asymmetric unit with a calpastatin bound to each monomer. The Cα RMSD between the calpain chains is 0.28 angstroms. Calpain chain A and calpastatin chain C were used for the design as the b-factors of residues at the calpastatin binding site were lower than in the other interface. Before designing, the entire protein was repacked using the pack rotamers routine and backbone and side chain dihedral angles were minimized using gradient based minimization.

To select mutations for the initial rounds of experimental testing we performed 500 independent runs allowing all NCAAs at a single position while keeping the sequence of the other positions fixed for each position in the peptide. Additionally we performed 256 independent runs where we individually tried each NCAA at each position in the peptide. Results were evaluated based on the total energy of the structure and the predicted binding energy of the structure calculated as the difference in energy of the complex and the unbound chains.

### Purification of Calpain, Calpastatin, and Calpastatin Mutants

Calpain was expressed as a GST-fusion protein in *E. coli* that had been transfected with a pet41b vector that contained the gene encoding porcine calpain-1. The cells were grown in Luria-Bertani broth with 50 ug/ml kanamycin at 37 degrees Celsius to an OD_600_ of 0.6 at which point 1 mM isopropyl β-D-1-thiogalactopyranoside (IPTG) was added to induce expression. Cells were grown for an additional 4 hours and harvested by centrifugation. Cells were resuspended in a buffer containing 50 mM NaPO_4_, 150 mM NaCl, 5 mM BME at pH 8.0 and lysed by sonication. Lysate was centrifuged at 12500 g for 30 minutes and the supernatant was run over a GSTrap-FF column that had been equilibrated with the lysis buffer. After loading, the column was washed with 10 column volumes of the lysis buffer before the GST-calpain was eluted with 50 mM Tris and 10 mM reduced glutithione, pH 8.0. The eluent was monitored by absorbance at 280 nm.5 mL fractions were collected. Those fractions which absorbed at 280 nm were pooled. Thrombin was added to separate the calpain from the GST and the cleavage reaction was allowed to cleave overnight at 4 Celsius. To remove the GST the protein was further purified with a Sephacryl S-200 gel filtration column using a buffer containing 50 mM NaPO_4_, 50 mM NaCl, 5 mM BME, 1 mM CaCl_2_, 1 mM EDTA at pH 8.0. The protein was concentrated through centrifugation via a spin column with a 15 K molecular weight cutoff and found to be pure and ran true to predicted size on SDS-PAGE gels.

Seven peptides were synthesized for experimental validation (table 1). The wild type and 4-methyl-phenylalanine (4MF) mutant peptides were synthesized by the Tuffs University Core Facility. These sequences were labeled with a fluorescein isothiocyanate (FITC) dye through an N-terminal β-alanine (βALA) linker. Peptides for the 1-methyl-histidine (1MH), amino-butyric acid (ABU), homoserine (HSE), and norvaline (NVL) mutant peptides were synthesized by the Peptide Synthesis and Analysis Facility in the Strahl laboratory at the University of North Carolina at Chapel Hill. These peptides were labeled with a 5-carboxyfluorescein (5FAM) dye also through a N-terminal βALA linker.

**Table 1:** Summary of the Rosetta energy predictions for the redesign of the calpain/calpastatin interface and experimentally determined disassociation constants.

Position	Peptide Sequence	Predicted (REU)		Experimental K_D_ (µM)
		Total	Binding	
WT	FITC–βALA–PDDAIDALSDDFTS-amide	−57.9	−13.4	5.77±0.31
	5FAM–βALA–PDDAIDALSDDFTS-amide	−57.9	−13.4	6.60±0.65
607	5FAM–βALA–PDDAIDAL(ABU)DDFTS-amide	−61.9	−15.1	5.80±1.20
	5FAM–βALA–PDDAIDAL(NVL)DDFTS-amide	−61.9	−14.4	8.56±0.95
609	5FAM–βALA–PDDAIDALSD(1MH)FTS-amide	−61.8	−15.2	5.81±0.64
	5FAM–βALA–PDDAIDALSD(HSE)–FTS-amide	−59.5	−14.2	7.48±0.68
610	FITC–βALA–PDDAIDALSDD(4MF)TS-amide	−60.0	−14.2	2.60±0.25

### Fluorescence Polarization Binding Assays

Purified calpain was manually titrated in to a solution containing 500 nM calpastatin with 50 mM NaP, 50 mM NaCl, 5 mM BME, 1 mM CaCl_2_, 1 mM EDTA at pH 8.0, till the change in fluorescence polarization reached a plateau. Binding assays were performed at room temperature, 3 polarization readings were averaged for each concentration. Disassociation constants were calculated by fitting the data to a single state binding model using Sigma plot software.

## Results

### Energy Function Modifications required for NCAAs

#### Explicit Unfolded State Energy

Using peptides to model the unfolded state, we have calculated average unfolded state energies for the 20 CAAs and the 114 NCAAs that we have added to Rosetta. This term captures the average internal energy of each of the amino acids, Lennard-Jones and torsional energies, as well the average strength of non-covalent interactions with residues close in primary sequence. For the amino acids with six membered rings (PHE, TYR and TRP) repulsive energies are calculated between the 1 and 4, 2 and 5, and 3 and 6 carbons, which leads to large unfavorable unfolded state energies. However, these repulsive energies largely cancel out when the unfolded state energies are subtracted from the energy of the folded state. During optimization of the modified energy function an overall weight was placed on the unfolded state score of the protein, the final fitted value was close to one and had a value of 0.91. In the standard version of Rosetta the unfolded state energies, referred to as reference values, are not explicitly calculated for the amino acids but rather are empirically determined to reproduce naturally occurring amino acid compositions when redesigning large sets of proteins. A direct comparison between the ‘standard’ Rosetta reference values and the new unfolded state values is not meaningful because of differences in the way intra-residue energies are calculated.

#### Optimization of energy function component weights

The weights on the energy function terms have been optimized using a procedure that maximizes sequence recovery when redesigning a set of proteins (see methods, table 2). The weights on the Lennard-Jones inter residue attractive term were kept fixed during the weight fitting while all others were allowed to be free. The weights on the terms shared by standard Rosetta and the modified version remain similar with the exception that the Lennard-Jones inter-residue repulsive energy and solvation energy were upweighted.

**Table 2:** Weights on the stock Rosetta energy function and on the modified energy function.

Energy Term	Stock Weight	Modified Weight
Inter-repulsive	0.44	0.63
Inter-attractive	0.80	0.80
Solvation	0.65	1.16
Pair	0.49	-
Bb/bb HB	0.59	0.67
Bb/sc HB	1.17	1.45
Sc/sc HB	1.10	1.19
Dunbrack	0.56	-
Omega	0.50	-
Rama	0.20	-
Reference	1.00	-
Torsion	-	0.27
Intra-repulsive	-	0.32
Intra-attractive	-	0.54
Unfolded	-	0.90

#### CAA Sequence and Rotamer Recovery

Sequence and rotamer recovery benchmarks were run using the standard Rosetta and modified energy functions as described in the methods section. χ1 rotamer recovery for the stock energy function was 84% overall, 93% in the core, and 74% on the surface. χ1 and χ2 rotamer recovery for the stock energy function was 64% overall, 74% in the core, and 53% on the surface. χ1 rotamer recovery for the modified energy function was 75% overall, 91% in the core, and 59% on the surface. χ1 and χ2 rotamer recovery for the modified energy function was 53% overall, 71% in the core, and 37% on the surface. The overall sequence recovery was 35% for the standard Rosetta energy function and 28% for the modified energy function. When the weight fitting protocol is run using the standard energy function with the reference energy term replaced with the explicit unfolded energy term the sequence recovery is 30%.

Rotamer recoveries between the two energy functions are nearly identical for buried residues indicating that the modified energy function performs well when there are multiple constraints dictating side chain conformation. On the protein surface, the internal energy of the side chains play a larger role in determining their conformations and the modified energy function performs well, but not as well as the standard Rosetta knowledge-based potential. This result highlights the usefulness of knowledge-based potentials. However, in most practical applications of design with NCAAs we will be interested in buried positions, either in a protein core or at an interface, and in these situations both potentials perform equally well. The standard Rosetta potential also has higher rates of sequence recovery than the modified energy function. This is partially because the new energy function has 20 less degrees of freedom when weight fitting, because there are no longer adjustable reference energies for each amino acid [42], [43]. Additionally, the fragment based method of calculating unfolded energies typically places the central residue (the residue for which unfolded state energies are being estimated) in a position where it experiences far fewer contacts than if it were in a folded protein or at a protein interface. Thus, the largest systematic bias in our unfolded-state energies, stemming from this low contact density, is that our unfolded term under estimates the attractive component of the energy function for larger amino acids. This gives larger amino acids a bias when designing because they contain more atoms.

### Rotamer Library Creation

#### Canonical Amino Acid Rotamer Library Creation and Validation of MakeRotLib

Rosetta currently uses the 2002 update to the Dunbrack backbone-dependent rotamer library [24]. To test the MakeRotLib protocol we have used it to create rotamer libraries for all CAAs except alanine, glycine and proline, and compared them to the Dunbrack backbone-dependent rotamer library [24]. We use the notation developed by Lovell *et al.* to describe the rotamers because of its clarity and brevity [44]. We compare the libraries produced by each method using the percent overlap of matching rotamer bins and the RMS distance in degrees of the matching rotamer bins. Overall we see good agreement between the Dunbrack rotamer libraries and those generated by the MakeRotLib protocol. All CAAs but Asn and Asp have a more than 70% overlap in rotamer bins. For all matching rotamer bins the average RMS deviation is 9.5 degrees. As would be expected, the CAAs with the best percent overlap are those with fewer degrees of freedom as evidenced by all amino acids with one χ angle getting at least 88% percent overlap. Deviations between the rotamer libraries are most often seen in ϕ/ψ bins where there are few counts in the Dunbrack library. The MakeRotLib protocols perform less well for short polar amino acids, ASN and ASP perhaps because there are electrostatic interactions between these side chains and backbone atoms not well modeled by our procedure. For large aromatics (Phe and Tyr) we see less accuracy in the prediction of the most favorable χ, this is probably because these minima are less sharp in nature as well. The highest, lowest and average percent overlap and RMS distance for each CAA over all populated ϕ/ψ bins are shown in table 3. The results for Leu, Asn, and Phe are described below, the results for the other CAAs are described in the Supporting Information S1.

**Table 3:** Comparison of the top 95% of CAA rotamers predicted by the MakeRotLib protocol to the rotamers given by the Dunbrack rotamer library.

CAA	RMS Distance (degrees)			Percent Overlap (%)		
	lowest	highest	average	lowest	highest	average
ARG	5.8	11.2	7.7	57	100	87
ASN	0.3	18.1	12.6	0	100	67
ASP	6.8	21.1	13.5	0	100	58
CYS	0.2	15.1	6.1	50	100	98
GLN	11.1	18.5	15.0	33	100	76
GLU	6.1	15.7	8.8	43	100	71
HIS	7.9	17.1	12.1	60	100	86
ILE	4.0	18.7	9.7	50	100	81
LEU	1.7	19.9	9.4	0	100	72
LYS	2.8	10.0	5.6	36	100	79
MET	3.3	10.4	5.9	56	100	86
PHE	3.0	17.5	11.7	50	100	93
SER	0.3	19.4	7.0	50	100	97
THR	0.0	27.8	7.8	0	100	91
TRP	5.4	14.7	9.0	33	100	73
TYR	1.4	20.7	11.5	0	100	93
VAL	1.5	21.9	8.6	50	100	88

Low, high, and average values (see methods) are calculated over all ϕ/ψ bins where the Dunbrack rotamer library reports more than 10 observations. A high percent overlap (see methods) indicates that the rotamers predicted by the MakeRotLib protocol are in agreement with the rotamers predicted by the Dunbrack rotamer library. A low average RMS distance indicates that the dihedral angles for rotamer bins that overlap are in good agreement.

#### Leucine

Leucine is an example of a CAA in which the MakeRotLib protocol performs well. Leucine has 2 χ angles with 3 χ1 rotamer wells (**mp** and **t**) and 3 χ2 rotamer wells (**mp** and **t**), for a total of 9 rotamers. At the −110/130 ϕ/ψ bin the top 4 rotamers comprise 98% of the Dunbrack rotamers and 100% of the MakeRotLib rotamers, while at the −60/40 ϕ/ψ bin the top 3 rotamers comprise 97% of the Dunbrack rotamers and 99% of the MakeRotLib rotamers. Overlap for the −110/130 ϕ/ψ bin is 75% while overlap for the −60/−40 ϕ/ψ bin is 100%. Both the Dunbrack rotamer library and the MakeRotLib protocol favor the **mt** and **tp** rotamers in most ϕ/ψ bins with probabilities >90%. Major differences in the overlap are generally the result of different preferences in the third and/or fourth most favorable rotamer (*i.e.* major differences are found primarily in higher energy, rarer, rotamers). Of the overlapping rotamers the average RMS angle distance is 9.5° for the −110/130 ϕ/ψ bin and 11.2° for the −60/−40 ϕ/ψ bin. χ1–2 rotamer angles cluster well around −60, 60, and 180. The **tt** rotamer is often skewed, to 190°, 140° by the MakeRotLib protocol; this skew can place the rotamer out of overlap range and therefore decrease the overall overlap. The skew is not consistent with the Dunbrack rotamer but is consistent with the preferred angles of Lovell *et al.* [44].

#### Aspargine

Aspargine is an example of where the MakeRotLib protocol has difficulty modeling. Asparagine has 2 χ angles with 3 χ1 rotamer wells (**mpt**), 6 χ2 rotamer wells (centered on −120, −60, −10, 40, 80, 140). At the −110/130 ϕ/ψ bin the top 9 rotamers comprise 96% of the Dunbrack rotamers and 97% of the MakeRotLib rotamers, while at the −60/40 ϕ/ψ bin the top 10 rotamers comprise 96% of the Dunbrack rotamers and 99% of the MakeRotLib rotamers. Overlap for the −110/130 ϕ/ψ bin is 56% while overlap for the −60/−40 ϕ/ψ bin is 80%. Asparagine is a difficult residue to match because of the large number of rotamers that span a full rotation about the χ2 dihedral. Additionally the rotamers are biased by local interactions in common secondary structures and because it places polar groups from the side chain in close proximity to the polar groups in the peptide backbone [44], [45]. If we look at the distribution of the Dunbrack library for χ2 angles for rotamers that are seen more then 10% probability are we find they mostly fall near −60, −20, 20, and 60. Dunbrack uses a large number of rotamers to cover the spread of angles and the MakeRotLib protocol does not find rotamers with χ2 near 0. This significantly lowers the overlap. Additionally the χ1 preferences of the MakeRotLib protocol differ from the Dunbrack library which also lowers the overlap. The MakeRotLib protocol strongly favors rotamers with a χ1 of **m** followed by **p** and then **t** while the Dunbrack is more evenly distributed. The top rotamers predicted by the MakeRotLib protocol have a higher percent overlap for the α-helical region than the β-strand region. Of the overlapping rotamers the average RMS angle distance is 12.3 for the −110/130 ϕ/ψ bin and 15.2 for the −60/−40 ϕ/ψ bin. Our modified energy function additionally doesn't take into account internal electrostatic interactions that are important for the small polar amino acids like Asp and Asn. The MakeRotLib protocol does not take into account increases in rotamer stability induced through interactions with neighboring side chains such as hydrogen bonding. These interactions can bias rotamer libraries when using the probabilities to compute energies but may also prevent strained rotamers (which would be compensated by other beneficial interactions) from being sampled because they would not be included in the database [44].

#### Phenylalanine

Phenylalanine is an example of an amino acid where the MakeRotLib protocol produces rotamers that are less accurate. Phenylalanine has 2 χ angles with 3 χ1 rotamer wells (**mpt**), 2 χ2 rotamer wells (centered on 90 and 0), for a total of 6 rotamers. At the −110/130 ϕ/ψ bin the top 3 rotamers comprise 99% of the Dunbrack rotamers and 97% of the MakeRotLib rotamers, while at the −60/40 ϕ/ψ bin the top 3 rotamers comprise 96% of the Dunbrack rotamers and 99% of the MakeRotLib rotamers. Overlap for the −110/130 ϕ/ψ bin is 100% while overlap for the −60/−40 ϕ/ψ bin is 100%. The MakeRotLib protocol finds rotamers with χ1 of −60, 60, and 180 and χ2 of 90 and −40. The Dunbrack rotamers with χ2 centered on 0 are not seen. That rotamer well is wide as evidenced by the large standard deviations. Lovell *et al.* note that phenylalanine rotamers with a χ2 near 0 often have bond angle deviations that would not be captured by the MakeRotLib protocol and could account for the deviation away from 20 or −20 [44]. Of the overlapping rotamers the average RMS angle distance is 14.2 for the −110/130 ϕ/ψ bin and 16.7 for the −60/−40 ϕ/ψ bin. The overlap is good because the 40 degrees is close enough by our measure to be the same rotamer. The assumption of ideal bond lengths and bond angles speed up protein design calculations. If the same assumption is made during rotamer creation amino acids that show slight bond angle deviations in certain conformations can be obscured (e.g. phenylalanine and tyrosine). The ideal bond and angle assumption can also induce systematic biases in the shapes of rotamer wells as the only degrees of freedom are torsions.

Directly comparing the results of our protocol to those of knowledge-based rotamer libraries is currently the best test of MakeRotLib's performance. Our method of creating rotamers unfortunately suffers because it does not take into account electronic effects that have not been adequately captured by the molecular mechanics terms and our energy function which are captured by knowledge-based rotamer libraries. However, the knowledge-based rotamer libraries can be biased because of long range sidechain-sidechain interactions [17]. In this study we have identified that tryptophan rotamers with α-helical ϕ and ψ, like valine and leucine rotamers, are biased because of long-range effects typically present in an α-helix. Additionally for amino acids the size of arginine or larger, the dipeptide model system used in the protocol allows rotamers that place the amino acid side chain in a position that would clash with the backbone of neighboring side chains (i+1, i−1). This could however lead to more accurate sampling of rotamers at protein termini that would most likely be under represented in a knowledge based rotamer library.

### Noncanonical Amino Acid Rotamer Library Creation

The full list of NCAAs that were added to Rosetta and for which rotamer libraries have been created is listed in the Supporting Information S1. Here we present a few examples in detail.

#### 2-Indanyl-Glycine

**Figure 4:**
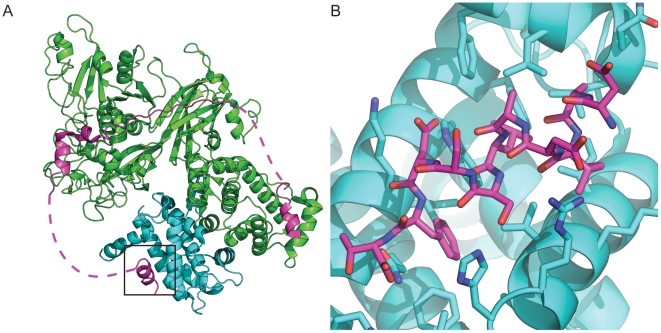
The structures of the example NCCA side chains. The structure of α-methyl-tryptophan is shown in a dipeptide context with ϕ = −150 and ψ = 150 (A). Plots of backbone the energy landscape of α-methyl-tryptophan and tryptophan (left) and canonical tryptophan (right) as calculated by Rosetta (B). Calculations were done in a didpeptide context where the backbone ϕ and ψ were fixed, the side chain was repacked and minimized for each ϕ and ψ bin in 5 degree intervals. Colors represent energy of the didpeptide in kcals/mol with red being the lowest energy and most preferred backbone conformation. The structure of homoserine in a didpeptide context with ϕ = −150 and ψ = 150 (C). The structure of 2-indynal-glycine is shown in a dipeptide context with ϕ = −150 and ψ = 150 (D). The different pucker state of the five member ring of 2-indynal glycine are modeled as separate amino acid type by Rosetta because of the difficulty in using rotamer libraries to capture coordinated movements that involved simultaneous rotation about multiple dihedral angles. There is a 1.45 kcal/mol energy difference between the “exo” conformer (left) and the “endo” conformer (right) with the “endo” conformer lower in energy.

2-indanyl-glycine is a hydrophobic amino acid that was initially synthesized as a constrained phenylalanine with particular χ1 torsional preferences.2-indanyl-glycine exists in 2 conformers due to the pucker of the 5-membered ring. The structures of both conformers are shown in figure 4. The “exo” conformer is 1.45 kcals/mol higher in energy than the “endo” conformer as determined by QM when both structures were minimized in preparation for rotamer creation. The amino acid has 1 χ angle about the Cα-Cβ bond. The 5-membered ring mimics the β-branched structure of valine and the rotamers are similar as shown in table 4. Both 2-indanyl-glycine and valine show a strong preference (>90%) for the **t** rotamer at both alpha-helical and beta-strand secondary structure conformations. χ1 distribution of the “exo” conformation has less spread than the “endo” because of the side chain backbone clashes that occur at rotamers other than **t**.

**Table 4:** The rotamers of 2-indanyl-glycine predicted by the MakeRotLib protocol with the rotamer for valine from the Dunbrack rotamer library for β-strand and α-helical ϕ and ψ.

Name	ϕ	ψ	Prob (%)	χ1	σ1
2IG “exo”	−110°	130°	0.9963	178.3°	10.0°
			0.0036	−76.3°	7.6°
			0.0001	73.7°	10.7°
	−60°	−40°	0.9990	177.8°	9.6°
			0.0009	−81.5°	7.2°
			0.0001	68.8°	10.3°
2IG “endo”	−110°	130°	0.9112	−179.9°	10.6°
			0.0834	−69.8°	8.9°
			0.0054	47.3°	6.5°
	−60°	−40°	0.9577	179.1°	11.6°
			0.0411	−72.0°	9.1°
			0.0011	43.8°	6.7°
VAL	−110°	130°	0.9408	178.0°	6.1°
			0.0338	57.8°	9.5°
			0.0254	−62.5°	12.7°
	−60°	−40°	0.9181	171.9°	5.2°
			0.0515	68.0°	10.1°
			0.0304	−61.0°	11.2°

#### α-Methyl-Tryptophan

α-Methyl-tryptophan is a tryptophan derivative that is taken up and retained by the brain because of it resemblance to serotonin. Labeled α-methyl-tryptophan is commonly used as a brain imaging tool [46]. It is identical to the canonical tryptophan amino acid with the addition of a methyl group replacing the Hα as seen in figure 4A. The addition of the methyl group restricts the rotamers that the side chain can adopt as shown table 5. The tryptophan χ2 rotamers near 0° occupy wide wells (represented in our libraries by large standard deviations, see table 4). The addition of the methyl group in α-methyl-tryptophan causes a clash with the χ2 = 0° rotamer and limits the rotamers that the amino acid can have to 6.The χ1 of α-methyl-tryptophan cluster around **m, p**, and **t** and the χ2 cluster around −90° and 90°. Additionally the methyl group also restricts the ϕ and ψ backbone dihedrals the residue can occupy, as shown in figure 4B. No structures have been deposited in the protein databank containing α-methyl-tryptophan.

**Table 5:** The rotamers of α-methyl-tryptophan predicted by the MakeRotLib protocol with the rotamer for tryptophan from the Dunbrack rotamer library for β-strand and α-helical ϕ and ψ.

Name	ϕ	ψ	Prob (%)	χ1	χ2	σ1	σ2
AMT	−110°	130°	0.5772	−70.9°	−91.7°	7.0°	9.8°
			0.2789	−173.9°	81.4°	4.3°	4.0°
			0.1065	−79.0°	76.8°	4.6°°	20.2°
			0.0258	44.1°	104.0°	4.8°	2.7°
			0.0109	175.8°	−91.3°	6.3°	5.8°
			0.0007	39.1°	−80.6°	7.6°	3.6°
	−60°	−40°	0.5034	−66.4°	−94.2°	10.6°	9.7°
			0.3157	−68°	88.1°	10.3°	10.2°
			0.1017	177.5°	87.1°	8.6°	7.0°
			0.0620	179.2°	−87.6°	9.4°	8.7°
			0.0100	40.3°	−77.6°	8.1°	5.1°
			0.0073	35.9°	102.8°	9.1°	6.6°
TRP	−110°	130°	0.5385	−69.0°	90.5°	6.3°	11.8°
			0.1645	−67.0°	3.4°	9.2°	23.4°
			0.1212	−69.7°	−92.5°	10.7°	10.2°
			0.0984	179.3°	−100.5°	15.7°	11.7°
			0.0660	178.9°	88.2°	5.3°	11.0°
			0.0091	−177.6°	18.0°	10.6°	26.6°
			0.0014	60.9°	−89.8°	9.3°	8.8°
			0.0008	61.5°	87.7°	10.0°	10.0°
			0.0001	66.0°	−6.3°	8.2°	42.3°
	−60°	−40°	0.2687	−179.3°	85.5°	7.7°	8.6°°
			0.2511	179.7°	−107.7°	11.7°	14.4°
			0.2030	−73.6°	109.2°	12.1°	14.5°
			0.1242	−70.5°	−11.5°	10.4°	22.2°
			0.0794	68.8°	−89.6°	7.4°	6.8°
			0.0516	−173.7°	16.7°	11.1°	36.1°
			0.0162	−89.8°	−119.8°	14.8°	22.4°
			0.0054	73.0°	91.3°	17.8°	12.0°
			0.0004	67.4°	−6.8°	7.8°	37.8°

#### Homoserine

**Figure 5:**
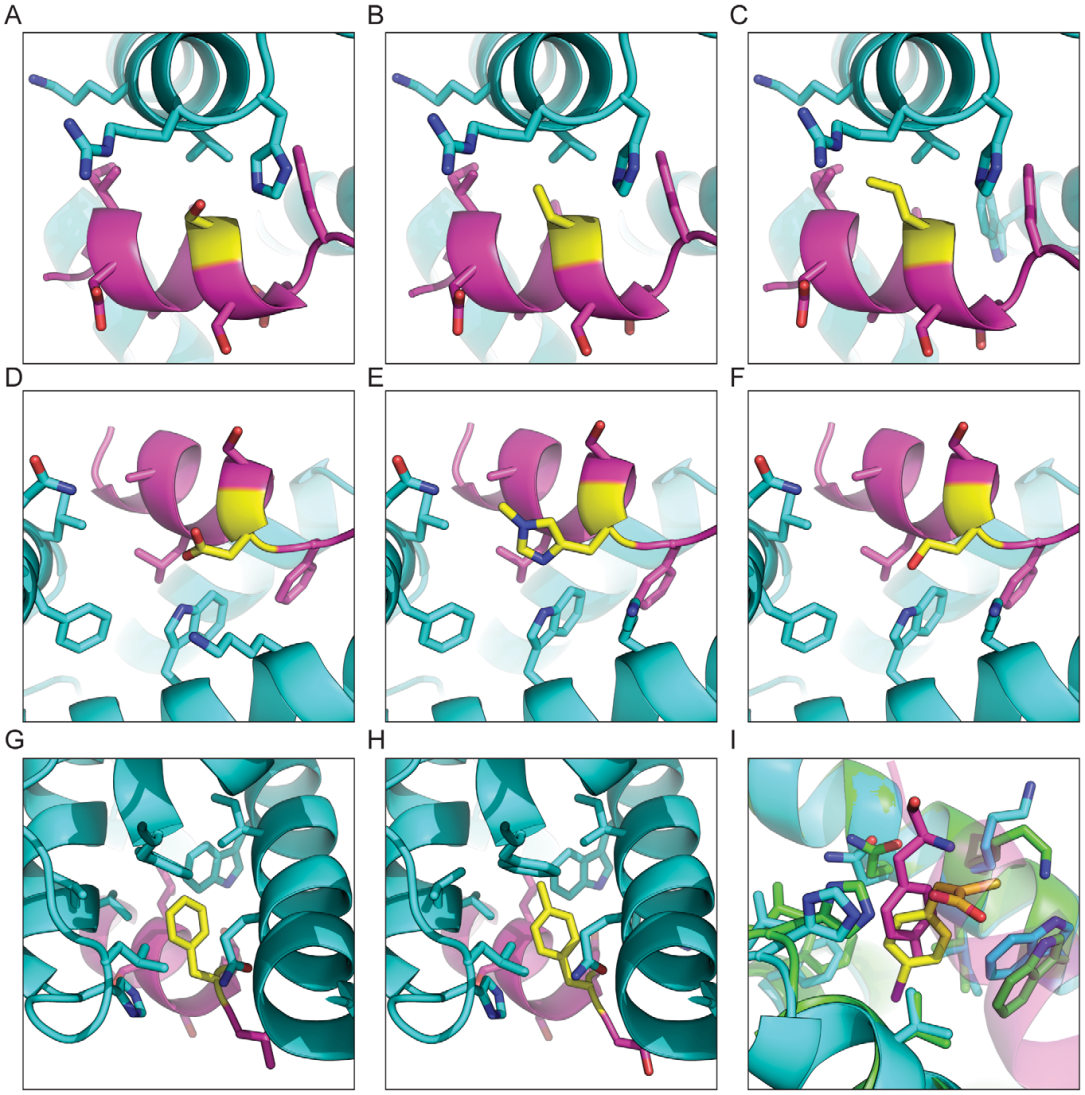
Rosetta predictions for experimentally tested calpain/calpastatin interface redesigns. Calpain is shown in cyan and calpastatin is shown in magenta, with the calpastatin position shown in yellow. Rosetta predictions for calpastatin position 607, wild type serine (A), amino-butyeiric acid (B), norvaline (C). Rosetta predictions for calpastatin position 609, wild type aspartic acid (D), 1-methyl-histidine (E), and homoserine (F). Rosetta predictions for calpastatin position 610, wild type phenylalanine (G), and 4-methyl-phenyl-alanine (H). Comparison of the PD150560 (yellow) inhibitor and predicted conformation of the 4-methyl-phenyl-alanine mutation at position 610 (I). The structure of 4-methyl-phenylalanine closely resembles that of the inhibitor and the orientation of PD150560 is identical to the predicted binding mode of the 4-methyl-phenylalanine.

Homoserine is a medium sized, unbranched, polar residue that has been added to Rosetta. Homoserine differs from the canonical serine due to the addition of a methylene group in the side chain, essentially making a longer serine residue (figure 4). Homoserine is a precursor in the biosynthesis of several amino acids. It is small and flexible and could be advantageous in designing hydrogen bonds at protein interfaces as seen in figure 5. χ1–2 cluster around the **m, p**, and **t** rotamers. The side chain is comparable to the χ1–2 of methionine with a **t** χ3 rotamer (table 6).

**Table 6:** The rotamers of homoserine predicted by the MakeRotLib protocol β-strand and α-helical ϕ and ψ.

Name	ϕ	ψ	Prob (%)	χ1	χ2	χ3	σ1	σ2	σ3
HSE	−110°	130°	0.7381	−58.9°	−62.8°		10.9°	12.8°	
			0.0790	−177.1°	56.7°		21.2°	22.8°	
			0.0649	−176.6°	176.6°		23.2°	26.6°	
			0.0621	−60.9°	177.8°		25.5°	26.6°	
			0.0368	−176.9°	−67.4°		4.3°	3.4°	
			0.0104	52.7°	178.6°		21.5°	24.7°	
			0.0075	−68.7°	69.9°		20.6°	20.9°	
			0.0010	51.4°	−77.3°		19.6°	14.6°	
			0.0001	54.3°	87.6°		18.4°	13.9°	
	−60°	−40°	0.6652	−178.3°	56.8°		0.1°	0.1°	
			0.1178	−59.1°	−62.1°		11.7°	12.2°	
			0.0939	−175.7°	174.7°		10.2°	11.1°	
			0.0850	−58.9°	−178.4°		11.1°	10.8°	
			0.0210	−169.4°	−74.3°		10.9°	10.2°	
			0.0089	−68.2°	69.6°		10.6°	12.1°	
			0.0079	48.9°	178.8°		10.4°	11.3°	
			0.0004	47.6°	−75.5°		0.1°	5.9°	
			0.0000	52.8°	88.0°		8.9°	7.5°	
MET	−110°	130°	0.0805	−62.6°	−178.7°	−177.0°	6.9°	11.5°	18.2°
			0.04913	178.2°	179.3°	−179.8°	6.6°	9.5°	13.6°
			0.04274	−60.2°	−65.2°	168.8°	6.2°	7.0°	21.8°
			0.02718	−170.4°	70.3°	−167.4°	10.7°	9.6°	16.7°
			0.00790	−175.8°	−86.1°	175.0°	11.4°	14.4°	17.8°
			0.00229	64.7°	−176.2°	−174.8°	7.3°	7.6°	19.1°
			0.00055	−78.9°	69.3°	−175.6°	13.8°	13.8°	23.8°
			0.000303	57.7°	78.1°	177.7°	17.2°	12.1°	26.3°
			0.000051	72.2°	−70.4°	174.3°	12.8°	14.7°	23.8°
	−60°	−40°	0.099862	−70.2°	178.0°	−178.0°	7.4°	8.4°	19.6°
			0.028043	−177.2°	177.1°	176.7°	10.6°	11.9°	22.7°
			0.021287	−172.5°	68.5°	−163.3°	6.6°	8.4°	24.7°
			0.019891	−65.7°	−63.7°	166.2°	7.4°	11.3°	28.5°
			0.001252	−179.7°	−82.3°	174.0°	10.8°	10.4°	17.0°
			0.001103	−78.5°	69.9°	172.4°	11.4°	11.4°	26.2°
			0.001103	65.8°	−174.4°	−175.2°	5.7°	6.4°	14.8°
			0.000023	57.7°	78.3°	177.6°	17.1°	12.3°	26.2°
			0.000004	72.2°	−70.4°	174.3°	12.8°	14.7°	23.8°

### Incorporation of NCAAs into the calpain/calpastatin interface redesign improves binding

As an initial test for our new methods in Rosetta for modeling NCAAs we examined if the software could be used to identify mutations to a peptide that would enhance its affinity for a target protein. Peptide design is an attractive arena for design with NCAAs because NCAAs are easily used in standard peptide synthesis protocols. Calpastatin binds as an amphipathic α-helix in a hydrophobic pocket between EF hands 1 and 2 of calpain DVI. Todd *et al.* identified calpastatin positions leu606 and phe610 as being the main residues involved in binding based on the crystal structure [31]. We have performed sequence optimization of the calpastatin peptide using a design protocol that iterates between backbone refinement and side chain design (see methods). 114 NCAAs were considered in the design runs. The results of the design runs were screened based on the predicted total energy and predicted change in binding energy. From preliminary simulations we noticed that it was not uncommon for the design protocol to favor substitutions to larger amino acids that would result in large structural perturbations to the interface when the backbone was relaxed. This is probably a consequence of the modified energy function which favors larger amino acids more than the standard Rosetta potential (see above).To avoid designs of this type, any designs where the peptide moved out of the binding groove were removed from consideration.

At positions 601, 603, 604, 605, and 608 of Calpastatin, Rosetta was unable to identify any mutations to CAA or NCAA that scored better than the wild type residue (table 1).The wild type serine at position 607 potentially forms a weak hydrogen bond with His129 (figure 5A), but is also surrounded by a hydrophobic packet formed by Val125, Ile603, and the methylene groups of Arg128.Substituting the serine with amino butyric acid (figure 5B) is predicted to increase the binding affinity by approximately 2 Rosetta energy units (REU) and norvaline (figure 5C) is predicted to increase the binding affinity by 1 REU. Neither of these mutations is predicted to affect the position of the peptide in the binding pocket.

The wild type aspartic acid at position 609 makes a hydrogen bond with Trp166, one of three hydrogen bonds between the peptide and the protein (figure 5D). The amino acids preferred by Rosetta keep this hydrogen bond intact. 1-Methyl-histidine (figure 5E) forms an ideal hydrogen bond with Trp166. The hydrogen to acceptor distance is 1.9 angstroms. The aliphatic part of the methyl-histidine packs against Phe99, Leu102, Lys170, and Ala605. Homoserine (figure 5F) is also able to make the hydrogen bond to trp166. The hydrogen to acceptor distance is 2.1 angstroms. The difference in functional groups between the aspartic acid and the homoserine allows the homoserine to form more ideal hydrogen bond geometry.

At position 610, the wild type phenylalanine is buried in a large hydrophobic pocket and along with Leu606 forms the main hydrophobic interface with calpain. The phenylalanine interacts with Trp166, His129, Leu132, Val125, Ile169, Phe224, and the hydrophobic portion of Gln173 (figure 5G). The crystal structure shows that the pocket is not entirely filled by the phenylalanine. Rosetta predicts that a 4-methyl-phenylalanine (figure 5H) can fill more of the cavity and creates more hydrophobic contacts without disrupting the overall binding, and would therefore have an increased binding affinity.

#### Fluorescence Polarization Binding Assays

Fluorescence polarization binding assays were conducted with five of the designed peptides, each containing a single point mutation: Ser607 to amino butyric acid (ABU), Ser607 to norvaline (NVL), Asp609 to 1-methyl-histidine (1MH), Asp609 to homoserine (HSE), and Phe610 to 4-methyl-phenylalanine (4MF) (Supporting Information S1). Except for Phe610 to 4MF, the peptides had affinities for Calpain that were the same as the wild peptide, within the errors of the experiment. The peptide with 4MF at position 610 showed a two-fold increase in binding affinity, 2.6 mM compared to 5.8 mM. It is encouraging that all the designs bind well to Calpain, indicating that the design procedure was able to find mutations that are compatible with the target interface, even in cases where the polarity of the amino acid is changed. The increase in binding affinity of Phe610 to 4MF is consistent with previous results that show that increasing buried hydrophobic surface are at an interface can improve binding affinity [47].

## Discussion

We have developed a version of the Rosetta energy function that is compatible with NCAAs. The performance of the new energy function is slightly worse than the standard Rosetta energy function in rotamer and sequence recovery tests, but still comparable to other programs that have been developed for this problem [48], [49].This new capability will allow the use of NCAAs in a wide variety of Rosetta protocols including procedures for modeling and designing proteins, RNA, DNA, enzymes, small molecules, surfaces and hybrid systems.

Rotamer libraries are a powerful tool in protein modeling. We have developed methods to create rotamer libraries that are compatible with NCAAs, and we have shown that they are able to find the majority of CAA side chain rotamers. Additional uses of the rotamer creation protocol could be the creation of context dependent rotamer libraries for situations that may be under- represented in protein structures and therefore difficult to model using knowledge-based potentials. Examples of such context dependent situations are pre/post proline positions, terminal positions, and common terminal modifications [50]. The assumption that amino acid side chains are rotameric has been discussed in the past, with the majority concluding that they are [22], [24], [44]. We have found via our comparison of our CAA libraries to known structures that low energy conformations are seen the most frequently; the average cluster-member/cluster-centroid distance is low for the lowest energy rotamers, and that the shape indicates that the energetic landscape local to a given rotamer conformation is well fit by a simple Gaussian or modal distribution. However, some of the higher energy rotamers (lower probability structures) do not fit well to a Gaussian distribution and do not appear to be rotameric (figure 2C).

The modification to Rosetta presented here allows for the design of peptides and proteins with NCAAs. The NCAAs added to this point have α-amino acid backbones. NCAAs do not however have to be simple side chain substitutions. Extensions of the tools created here could be applied to scaffolds other than just α-peptide backbone, such as peptoids [51] or other foldamers.

We have shown that including NCAAs in computational protein design can be used to increase the binding affinity of a peptide-protein complex. The design of small molecule inhibitors of calpain is an area of active research, and it is thought that molecules that bind outside the active site are more likely to be specific for calpain [26].The inhibitor 3-(4-iodophenyl)-2-mercapto-(Z)-2-propenoic acid (also known as PD150606) discovered by Wang *et al.* [52] binds to calpain in the same hydrophobic pocket as position 610 and resembles the 4MF predicted by Rosetta [31]. The structure of the inhibitor bound to the calpain has been solved (protein databank code 1NX3) and is shown superimposed with our design in figure 5. The high degree of structural similarity between the inhibitor and 4MF and the similarity between the predicted binding mode and the structure of the bound inhibitor gives us confidence that our peptide is binding in a similar fashion [31], [52]. Although it is clear that additional experimental screening needs to be developed and performed in additional model systems, we are encouraged by these results that suggest that Rosetta NCAA design (the novel procedure described here) can be used to optimize peptide-protein interfaces.

## Supporting Information

Supporting Information S1 **Description, list, and figures of all of the NCAAs.** Results, discussion and figures for the compassion of the MakeRotLib rotamer libraries and the Dunbrack libraries for CAAs other than Leu, Phe, and Asn. Fluorescence polarization binding curves for calpain and peptides with wild-type calpastatin sequence and designed sequences.
(PDF)

Supporting Information S2 **“Protocol capture” that includes descriptions, instructions, command lines and processing scripts to reproduce the NCAA parameter files, explicit unfolded state energies, and rotamer libraries.** Additionally the protocol capture includes descriptions, instructions, command lines and processing scripts used to redesign the NCAA peptides.
(GZ) Click here for additional data file.
