# Supplementary material for: Incorporation of Noncanonical Amino Acids into Rosetta and Use in Computational Protein-Peptide Interface Design
Source: PLoS One. 2012 Mar 14;7(3):e32637. doi: 10.1371/journal.pone.0032637 (PMC3303795; doi:10.1371/journal.pone.0032637)
Supplement: Supporting Information S1 — Description, list, and figures of all of the NCAAs. Results, discussion and figures for the compassion of the MakeRotLib rotamer libraries and the Dunbrack libraries for CAAs other than Leu, Phe, and Asn. Fluorescence polarization binding curves for calpain and peptides with wild-type calpastatin sequence and designed sequences. (PDF) [file pone.0032637.s001.pdf]

Incorporation of Noncanonical Amino Acids into Rosetta and use in Computational Protein-Peptide Interface Design: Supporting Information S1

Table of Contents

Supplementary Methods 2
Selection of Noncanonical Amino Acids 2
Supplemental Figure 1 3
Supplemental Figure 2 4
Supplemental Figure 3 4
Supplemental Figure 4 5
Supplemental Figure 5 7
Supplemental Figure 6 9
Supplemental Figure 7 10
Supplemental Figure 8 11
Supplemental Figure 9 12
Supplemental Figure 10 13
Supplemental Figure 11 14
Supplemental Figure 12 15
Supplemental Figure 13 16
Supplemental Figure 14 17
Supplementary Results 17
Comparison between Dunbrack Rotamer Library and MakeRotLib Rotamer Library for the CAAs 17
Arginine 17
Supplemental Figure 15 18
Aspartic Acid 18
Supplemental Figure 16 19
Cysteine 19
Supplemental Figure 17 20
Glutamine 20
Supplemental Figure 18 21
Glutamic acid 21
Supplemental Figure 19 22
Histidine 22
Supplemental Figure 20 23
Isoleucine 23
Supplemental Figure 21 24
Lysine 24
Supplemental Figure 22 25
Methionine 25
Supplemental Figure 23 26

Serine 26
Supplemental Figure 24 27
Threonine 27
Supplemental Figure 25 28
Tryptophan 28
Supplemental Figure 26 29
Tyrosine 29
Supplemental Figure 27 30
Valine 30
Supplemental Figure 28 31
Fluorescence Polarization Binding Assays 32
Supplemental Figure 29 32
Supplementary Bibliography 34

Supplementary Methods

Selection of Noncanonical Amino Acids

We have added the following list of NCAAs to Rosetta and generated rotamer libraries for the ones with rotatable side chain dihedrals. For convenience they have been grouped based on analogy to the CAAs or based on their primary characteristics. In all figures carbons are shown in grey, hydrogens in white, nitrogens in colbalt, oxygens in red, sulfers in yellow, fluorines in sky blue, chlorines in light green, bromines in rust brown, iodines in purple. NCAAs are shown in the context of an amino acid dipeptide with a phi or -150 and a psi of 150 degrees. Images were created using PyMol[1].

Supplemental Figure 1

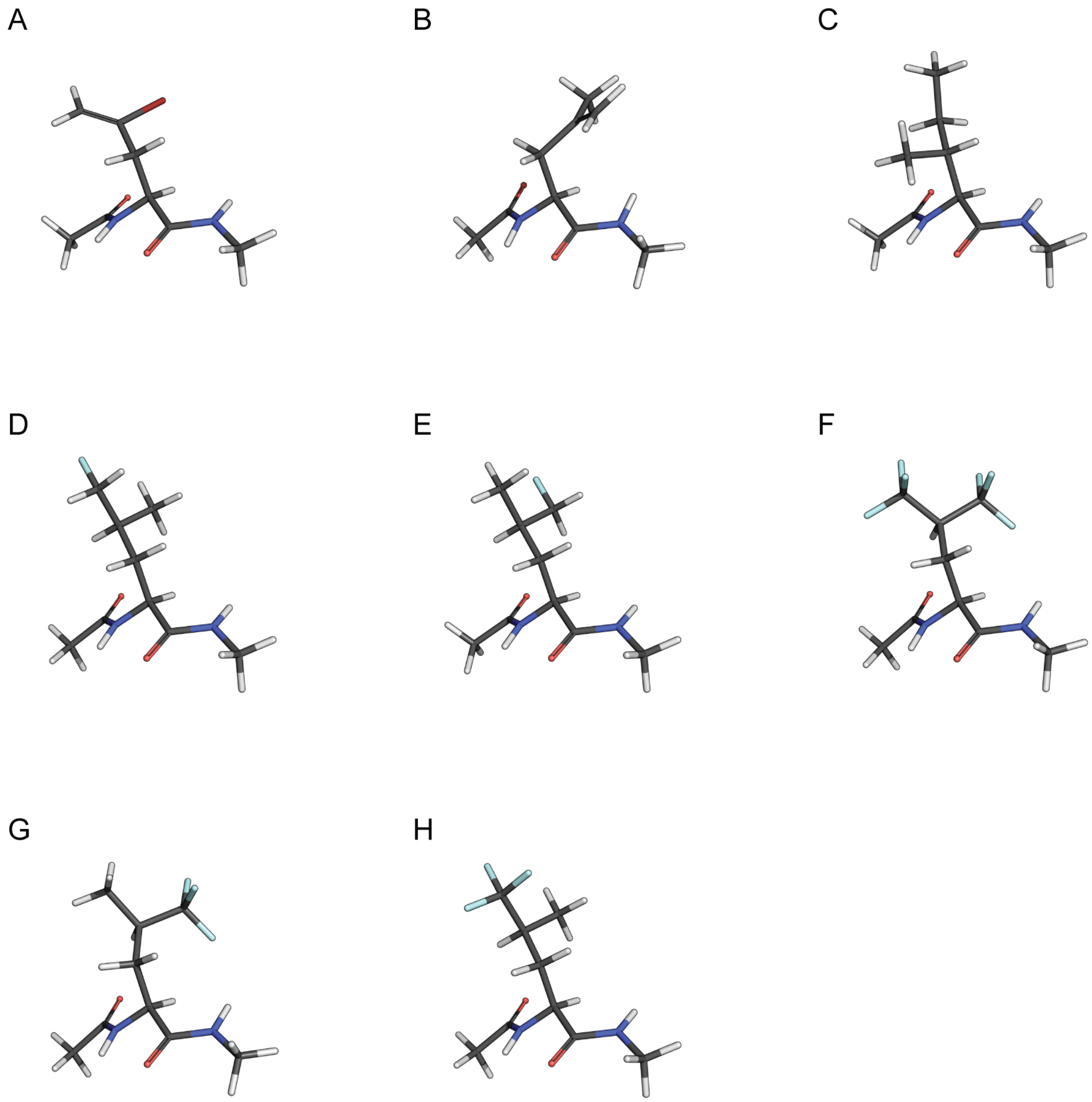

Analogs of Leucine and Isoleucine. (A) 2-amino-4-bromo-4-pentenoic acid, (B) 4.5-dehydro-leucine, (C) allo-isoleucine, (D) fluoro-leucine (enantiomer 1), (E) fluoro-leucine (enantiomer 2), (F) hexafluoro-leucine, (G) trifluoro-leucine (enantiomer 1), (H) trifluoro-leucine (enantiomer 2).

Supplemental Figure 2

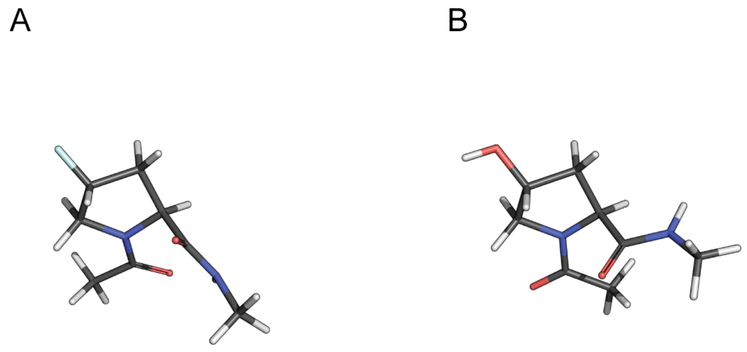

Analogs of Proline. (A) 4-fluoro-proline (1 of 2 conformers shown), (B) hydroxy-proline.

Supplemental Figure 3

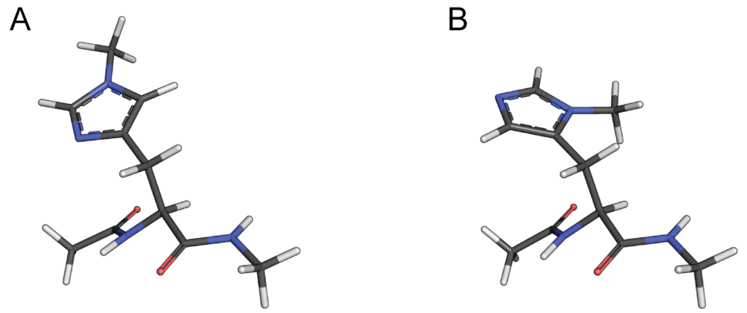

Analogs of Histidine. (A) 1-methyl-histidine (protonated not shown), (B) 3-methyl-histidine (protonated not shown).

Supplemental Figure 4

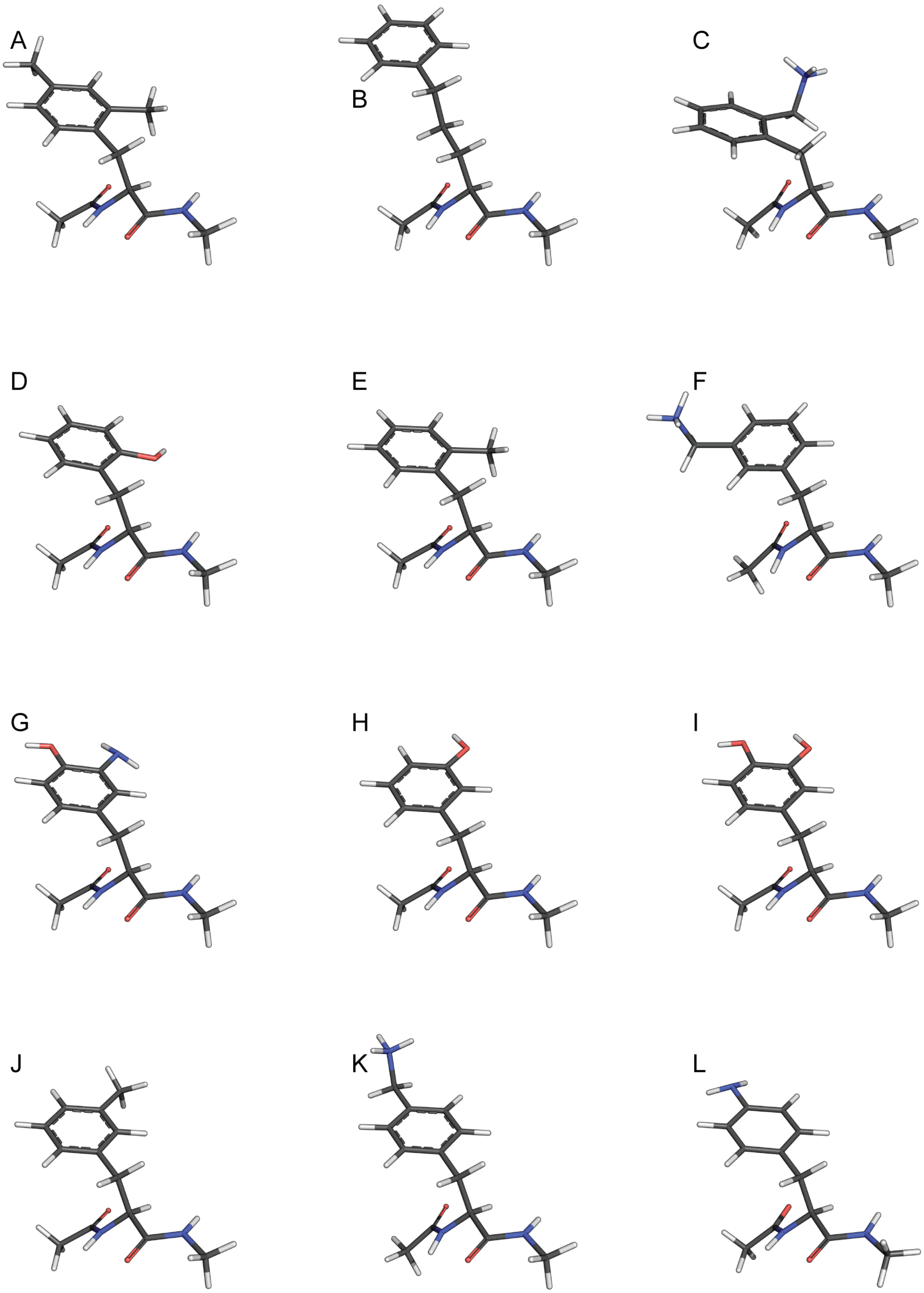

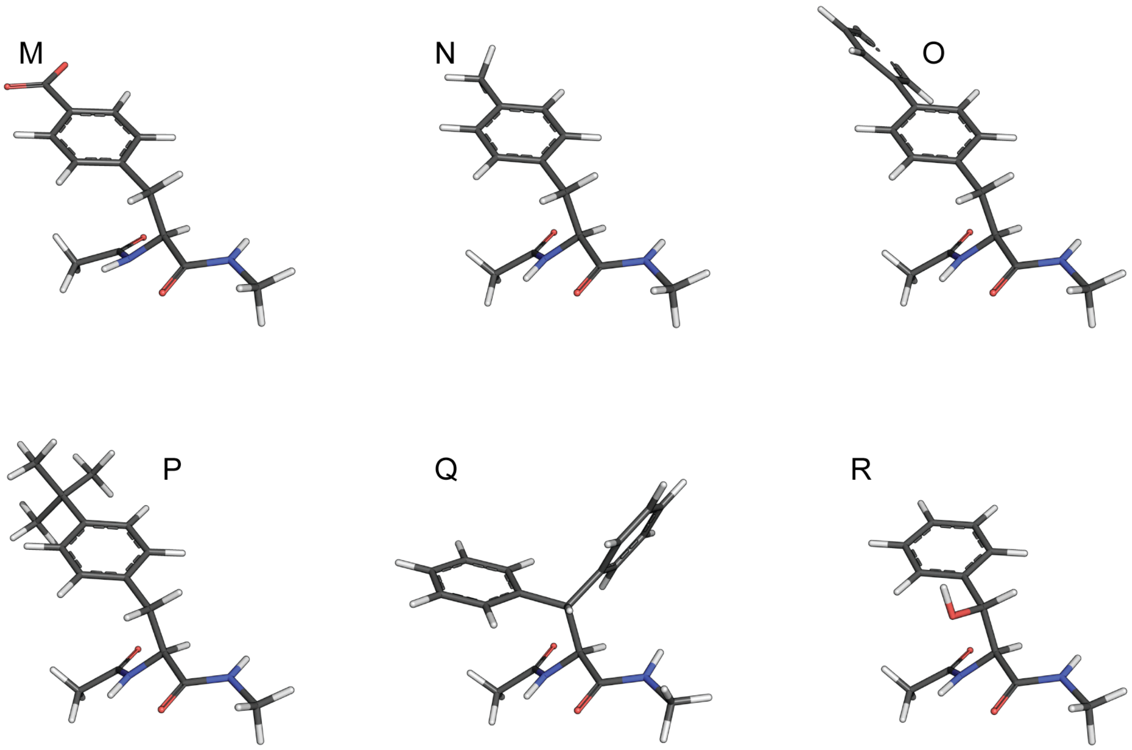

Analogs of Phenylalanine and Tyrosine. (A) 2.4-dimethyl-phenylalanine, (B) 2-amino-5-phenyl-pentanoic acid, (C) 2-aminomethyl-phenylalanine, (D) 2-hydroxy-phenylalanine, (E) 2-methyl-phenylalanine, (F) 3-aminomethyl-phenylalanine, (G) 3-amino-tyrosine, (H) 3-hydroxy-phenylalanine, (I) 3-hydroxy-tyrosine, (J) 3-methyl-phenylalanine, (K) 4-aminomethyl-phenylalanine, (L) 4-amino-phenylalanine, (M) 4-carboxy-phenylalanine, (N) 4-methyl-phenylalanine, (O) 4-phenyl-phenylalanine, (P) 4-tert-butyl-phenylalanine, (Q) β.β-diphenyl-alanine, (R) phenyl-serine.

Supplemental Figure 5

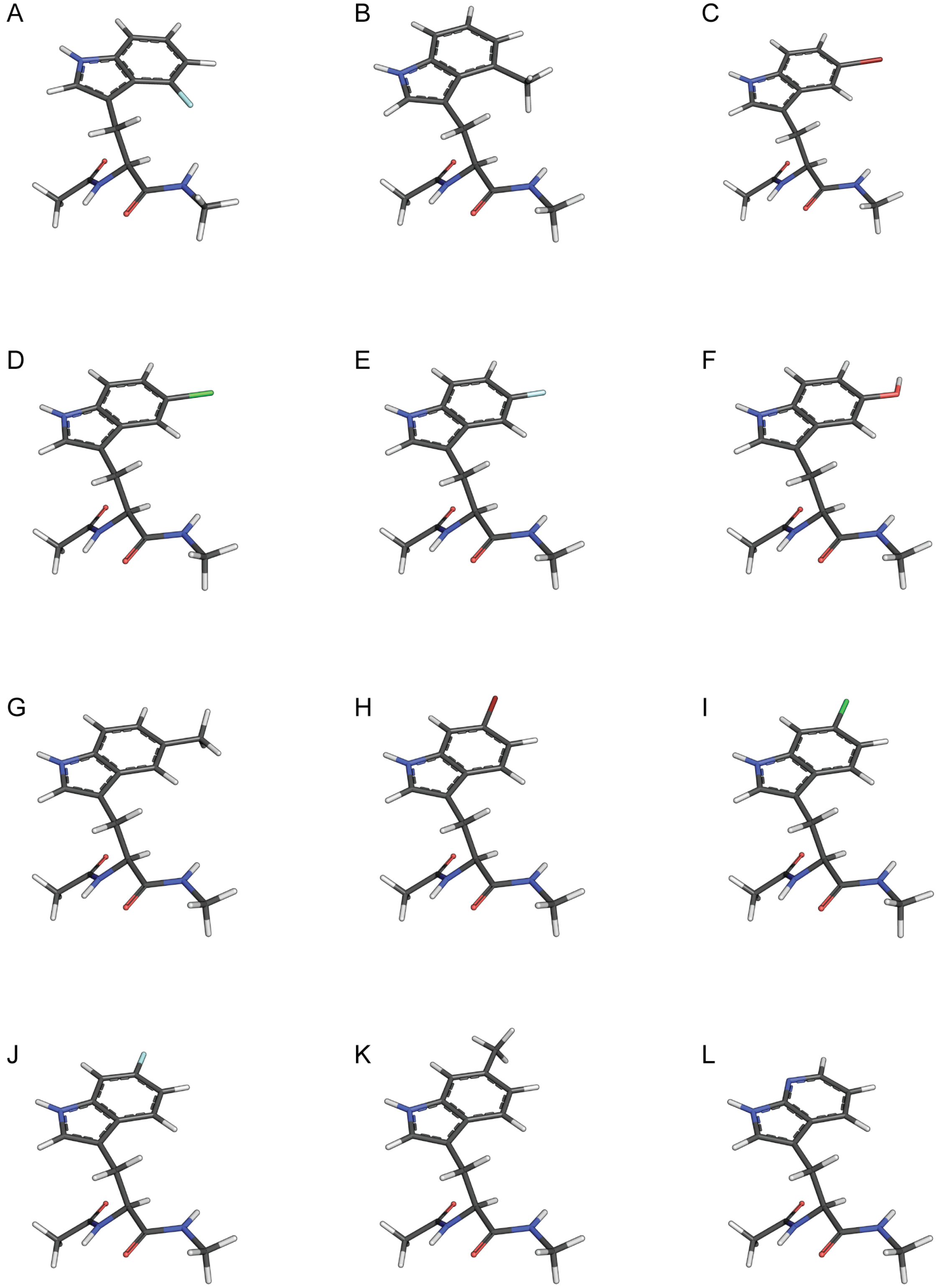

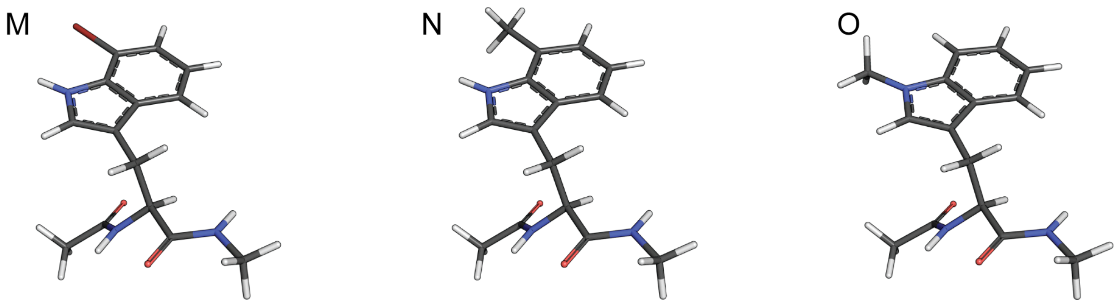

Analogs of Tryptophan. (A) 4-fluoro-tryptophan, (B) 4-methyl-tryptophan, (C) 5-bromo-tryptophan, (D) 5-chloro-tryptophan, (E) 5-fluoro-tryptophan, (F) 5-hydroxy-tryptophan, (G) 5-methyl-tryptophan, (H) 6-bromo-tryptophan, (I) 6-chloro-tryptophan, (J) 6-fluoro-tryptophan, (K) 6-methyl-tryptophan, (L) 7-azatryptophan, (M) 7-bromo-tryptophan, (N) 7-methyl-tryptophan, (O) n-in-methyl-tryptophan.

Supplemental Figure 6

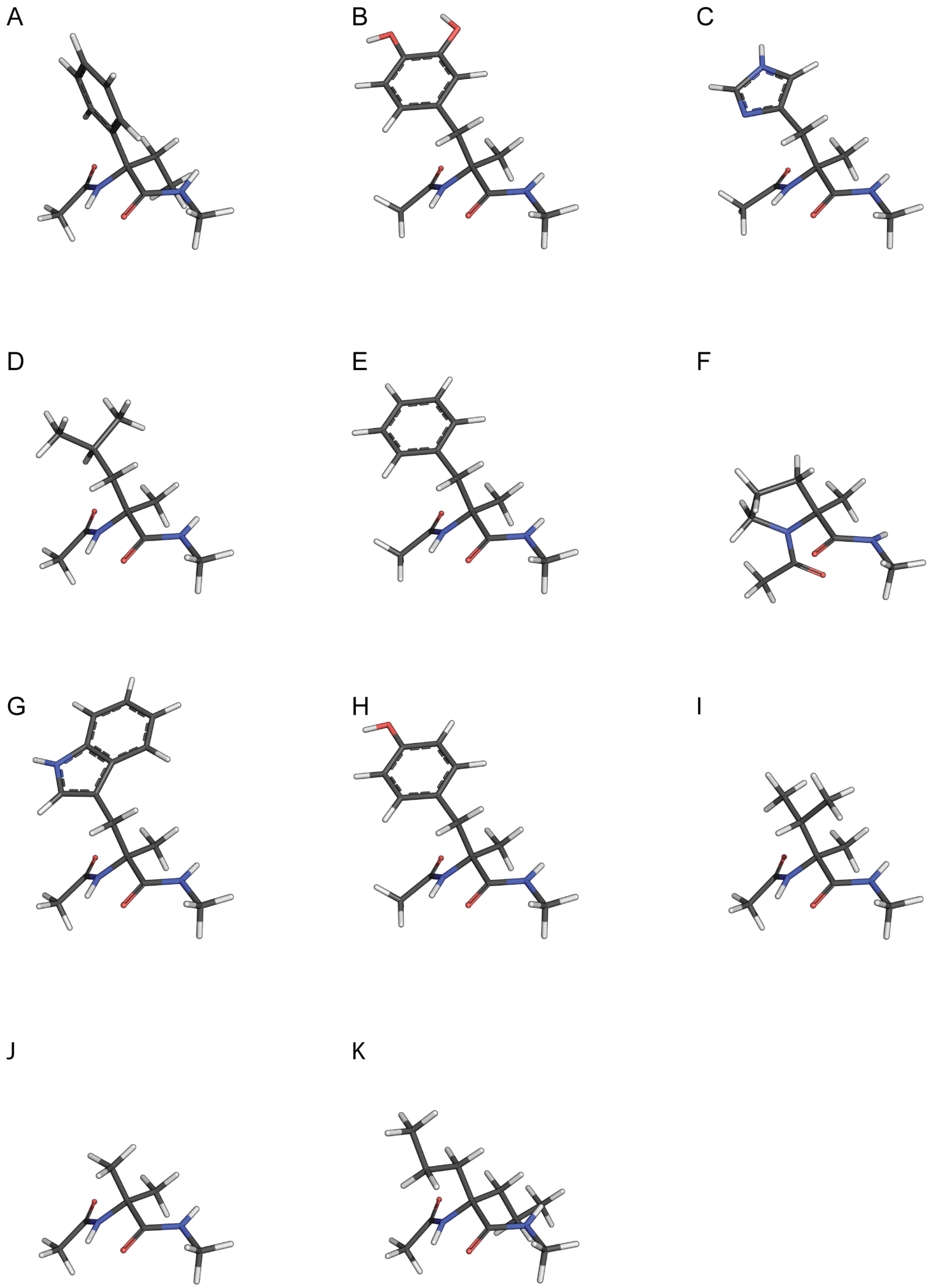

A-substituted. (A) 2-amino-2-phenylbutyric acid, (B) α-methyl-3-hydroxy-tyrosine, (C) α-methyl-histidine, (D) α-methyl-leucine, (E) α-methyl-phenylalanine, (F) α-methyl-proline, (G) α-methyl-tryptophan, (H) α-methyl-tyrosine, (I) α-methyl-valine, (J) aminoiso-butyric acid, (K) dipropyl-glycine.

Supplemental Figure 7

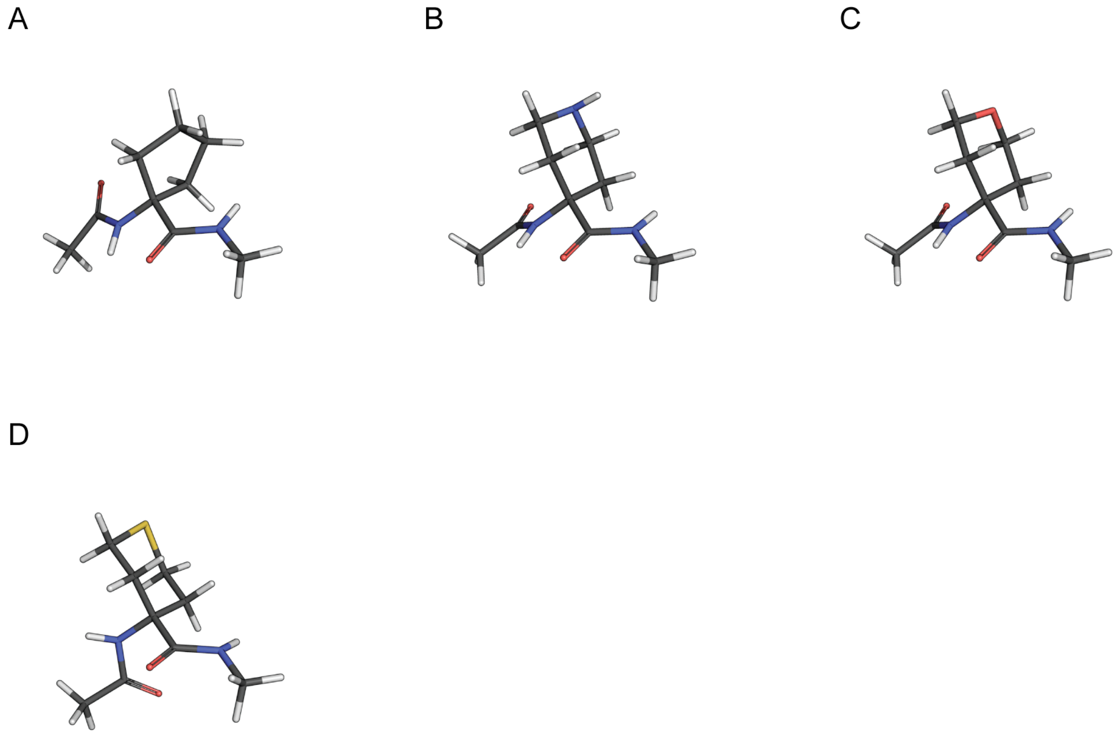

A-cyclic. (A) 1-amino-cyclopentane-carboxylic acid (2 conformers), (B) 4-amino-piperidine-4-carboxylic-acid (4 conformers), (C) 4-amino-tetrahydropyran-4-carboxylic acid (4 conformers), (D) 4-amino-tetrahydrothiopyran-4-carboxylic acid (4 conformers).

Supplemental Figure 8

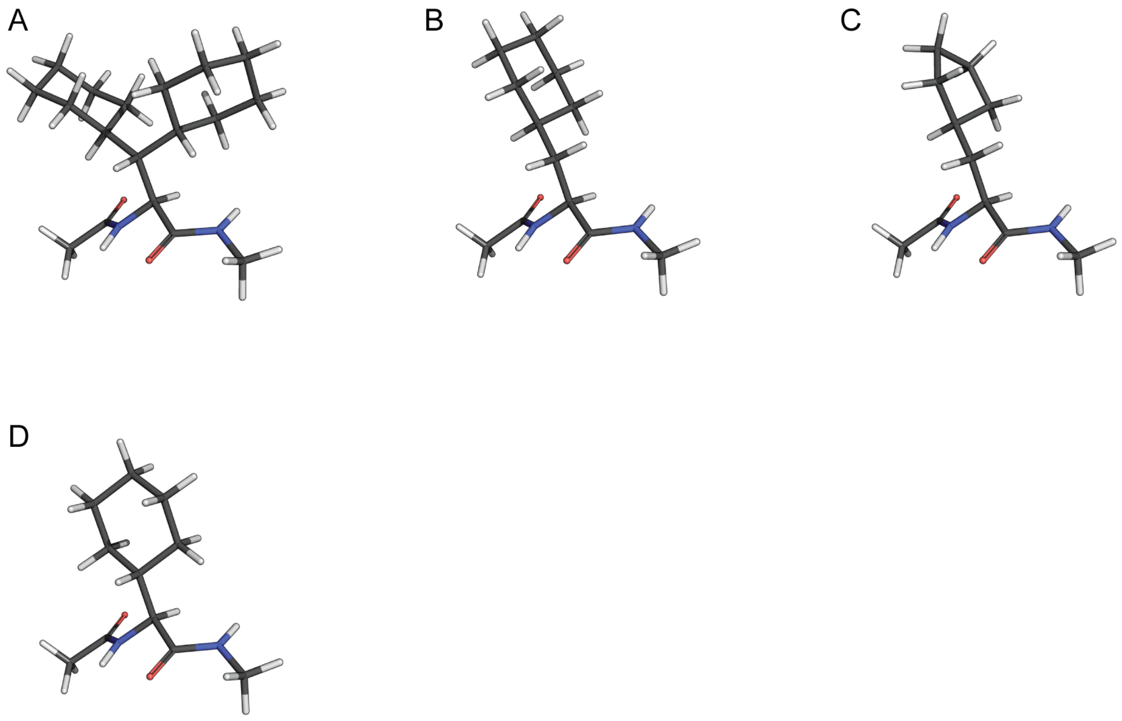

B-cyclic. (A) β-β-dicyclohexyl-alanine (4 conformers), (B) β-cyclohexyl-alanine (2 conformers), (C) β-cyclopentyl-alanine (2 conformers), (D) cyclohexyl-glycine (2 conformers).

Supplemental Figure 9

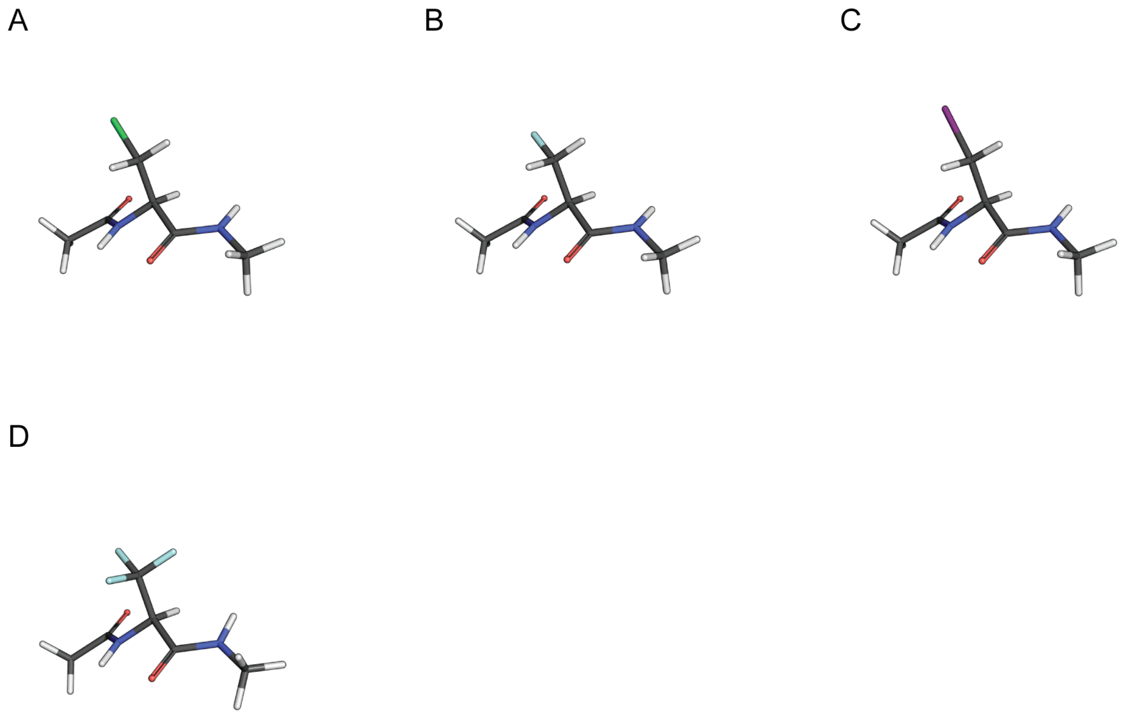

B-halo. (A) β-chloro-alanine, (B) β-fluoro-alanine, (C) β-iodo-alanine, (D) trifluoro-alanine.

Supplemental Figure 10

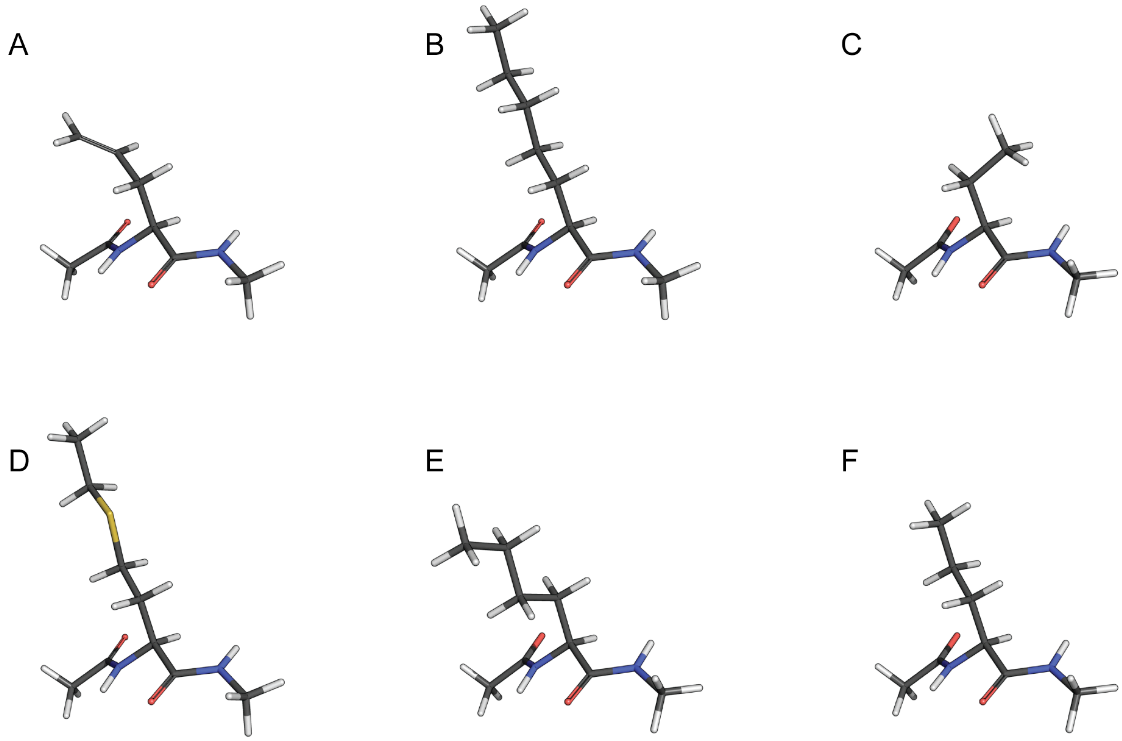

Single chain hydrophobic. (A) 2-allyl-glycine, (B) 2-amino-heptanoic acid, (C) amino-butyric acid, (D) ethionine, (E) norleucine, (F) norvaline.

Supplemental Figure 11

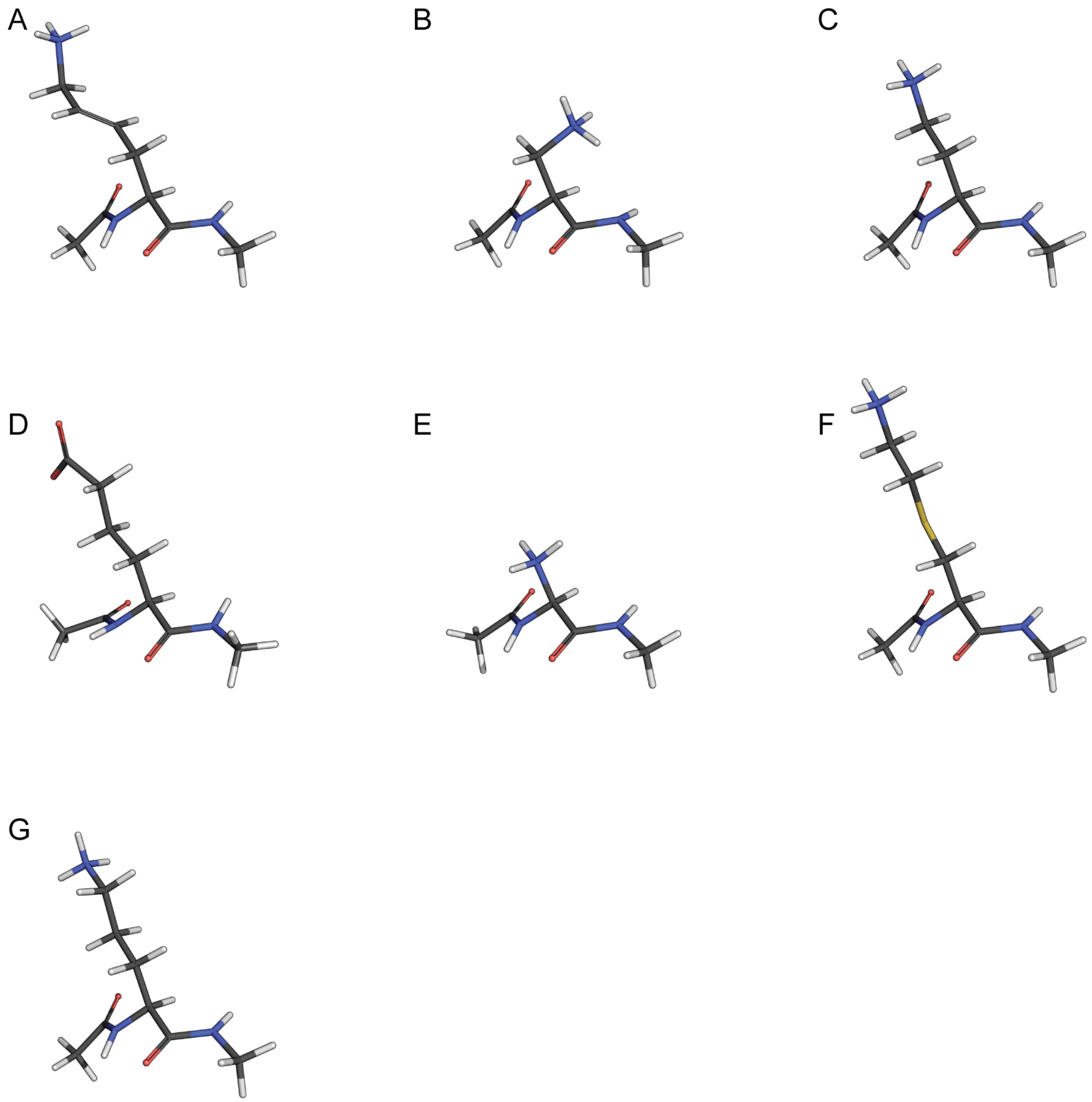

Single chain polar. (A) 4.5-dehydro-lysine, (B) α.β-diaminopropionic acid, (C) α-amino-glycine, (D) α-aminoadipic acid, (E) α. γ -diaminobutyric acid, (F) amino-ethyl-cysteine, (G) ornithine.

Supplemental Figure 12

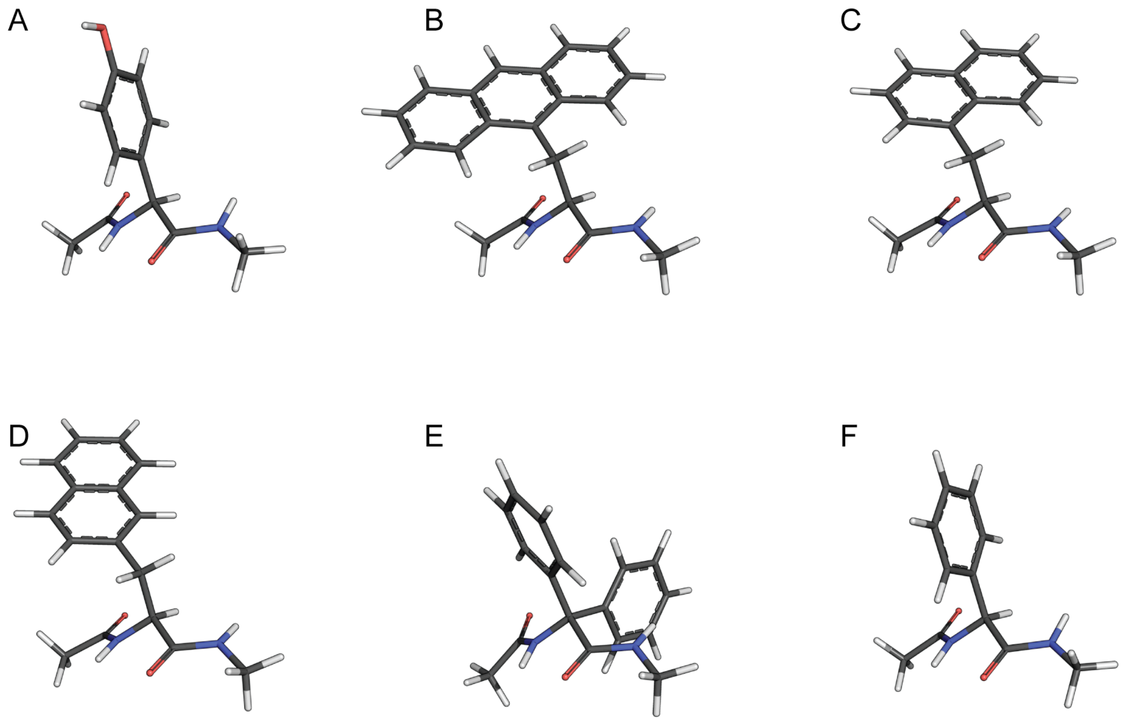

Other aromatic. (A) 4-hydroxy-phenylglycine, (B) 9-anthryl-alanine, (C) β-(1-naphthyl)-alanine, (D) β-(2-naphthyl)-alanine, (E) diphenylglycine, (F) phenylglycine.

Supplemental Figure 13

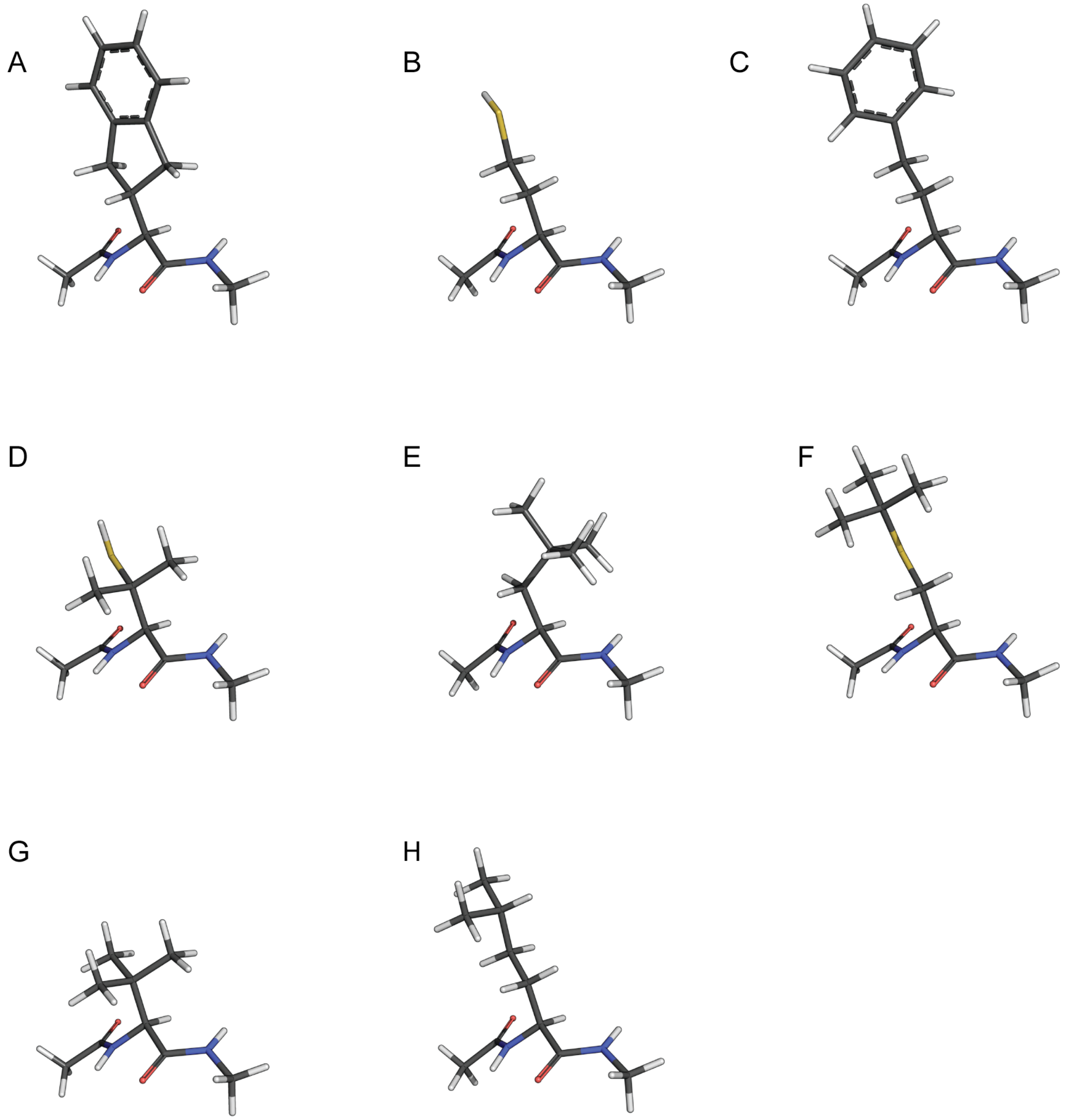

Other hydrophobic. (A) 2-indanyl-glycine (2 conformers), (B) homocysteine, (C) homophenylalanine, (D) penicillamine, (E) tert-butyl-alanine, (F) tert-butyl-cysteine, (G) tert-butyl-glycine, (H) homoleucine.

Supplemental Figure 14

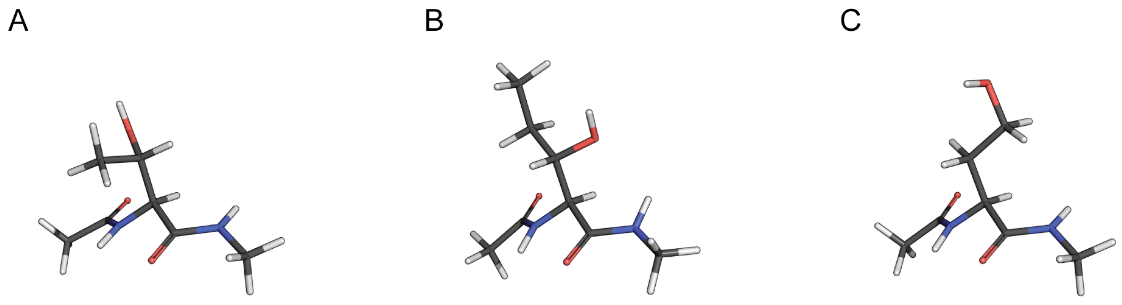

Other polar. (A) allo-threonine, (B) β-hydroxy-norvaline, (C) homoserine.

Supplementary Results

Comparison between Dunbrack Rotamer Library and MakeRotLib Rotamer Library for the CAAs

Arginine

Arginine has 4 χ angles with 3 χ1 rotamer wells (mpt), 3 χ2 rotamer wells (mpt), 3 χ3 rotamer wells (mpt), and 3 χ4 rotamer wells (mpt), for a total of 81 possible rotamers. At the -110/130 ϕ/ψ bin, the top 35 rotamers capture 97% of the Dunbrack rotamers and 95% of the MakeRotLib rotamers, while at the -60/40 ϕ/ψ bin, the top 35 rotamers capture 98% of the Dunbrack rotamers and 96% of the MakeRotLib rotamers. Overlap for the -110/130 ϕ/ψ bin is 77% while overlap for the -60/-40 ϕ/ψ bin is 91%. Deviations in the β-strand regions are due to the MakeRotLib protocol having a stronger preference for the χ1 m rotamer than the Dunbrack library. The MakeRotLib protocol favors compact side chain conformations with the side chain packing against the backbone. For example in the -110/130 ϕ/ψ bin the top rotamer is mmt-85 which is the 17th most popular Dunbrack rotamer. Dunbrack favors a more extended conformation as evidenced by the top 14 rotamers having a χ2 of 180. Low overlap probabilities occur in the regions where the Dunbrack library has low counts on the extreme of the β-sheet region and

the 3/10-helical region. Of the overlapping rotamers, the average RMS angle distance is 7.7 degrees for both the -110/130 and -60/-40 ϕ/ψ bins. χ1-3 rotamer angles cluster well around -60, 60, and 180. χ4 rotamers agree with the Dunbrack rotamers and cluster close to -85, 180, and 85. We do not see the χ4 = 105 rotamer in the -110/130 ϕ/ψ bin and only once in the -60/-40 ϕ/ψ bin. Others have shown however that when built with ideal bond angles there is a moderate clash but that in examples of that rotamer in crystal structures there are bond angle deviations that relieve the strain and permit the rotamer[2].

Supplemental Figure 15

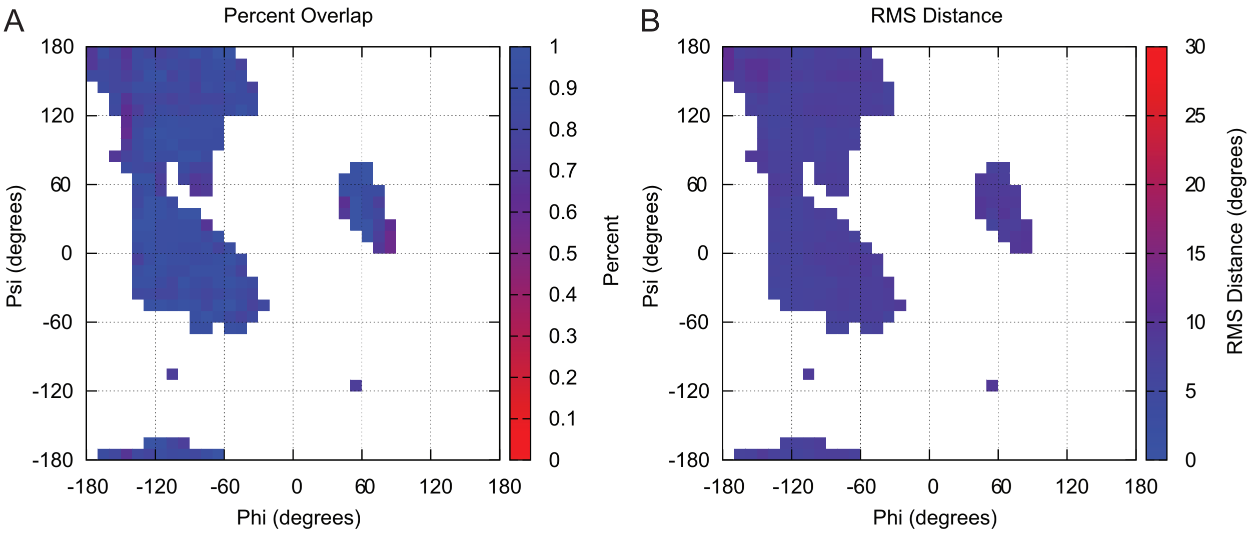

Comparing the percent of overlapping rotamer bins (A) and the RMS distance between overlapping bins (B) between the Dunbrack rotamer library and the rotamers predicted by the MakeRotLib protocol for arginine.

Aspartic Acid

Aspartic acid has 2 χ angles with 3 χ1 rotamer wells (mpt), 3 χ2 rotamer wells (centered on -60, 0, 60). At the -110/130 ϕ/ψ bin the top 6 rotamers capture 98% of the Dunbrack rotamers and 96% of the MakeRotLib rotamers, while at the -60/40 ϕ/ψ bin the top 6 rotamers capture 97% of the Dunbrack rotamers and 99% of the MakeRotLib rotamers. Overlap for the -110/130 ϕ/ψ bin is 67% while overlap for the -60/-40 ϕ/ψ bin is 67%. The MakeRotLib protocol finds rotamers with a χ1 of -60, 60, and 180. The χ2 rotamer wells depend on the χ1 with χ1 t centered on -70, 0, and 70, χ2 m centered on -50 and 90,

and χ1 p centered on -90. The MakeRotLib protocol is unable to match few of the χ2 rotamer wells near 0, which are often high probability in the Dunbrack library consequently lowering the overlap. Of the overlapping rotamers the average RMS angle distance is 14.3 for the -110/130 ϕ/ψ bin and 14.3 for the -60/-40 ϕ/ψ bin.

Supplemental Figure 16

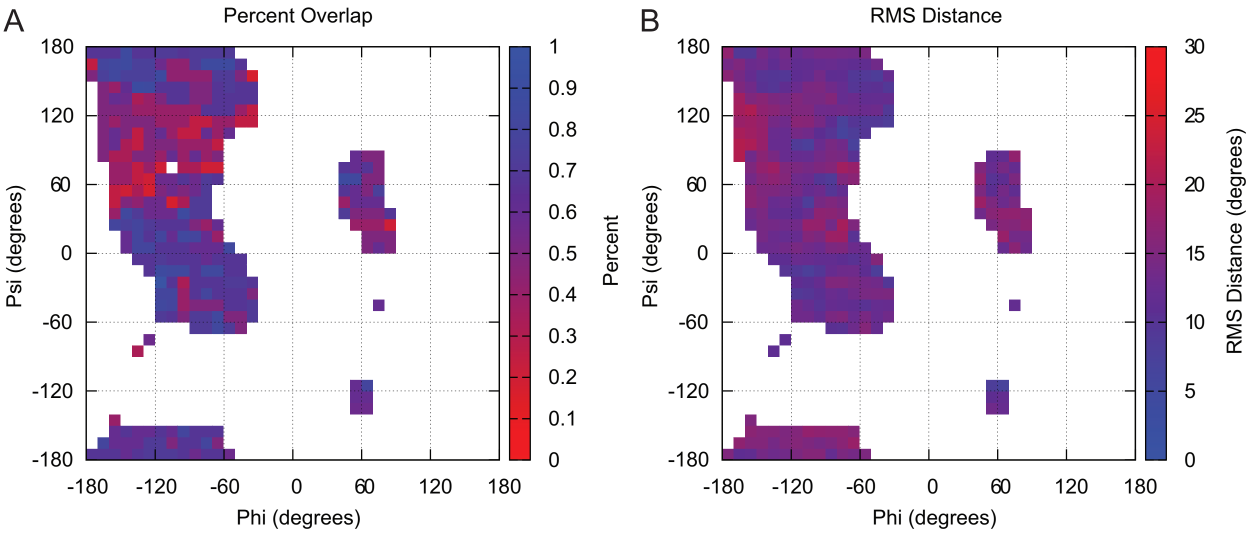

Comparing the percent of overlapping rotamer bins (A) and the RMS distance between overlapping bins (B) between the Dunbrack rotamer library and the rotamers predicted by the MakeRotLib protocol for aspartic acid.

Cysteine

Cysteine has 1 χ angle with 3 χ1 rotamer wells (mpt), for a total of 3 rotamers. At the -110/130 ϕ/ψ bin the top 2 rotamers capture 99% of the Dunbrack rotamers and 99% of the MakeRotLib rotamers, while at the -60/40 ϕ/ψ bin the top 3 rotamers capture 100% of the Dunbrack rotamers and 100% of the MakeRotLib rotamers. Overlap for the -110/130 ϕ/ψ bin is 100% while overlap for the -60/-40 ϕ/ψ bin is 100%. Overall the agreement between the MakeRotLib protocol and the Dunbrack library is the highest with an average percent overlap of 98% and an average RMS distance of 6.1 degrees. There is however a distinct ψ dependence shown in the banding pattern in figure 2. In the α-helical region the second most preferred rotamer shifts from t to p around the -20 and -30 ψ bins. The shift does not occur for the

MakeRotLib protocol. Lovell et al. [2] indicate that the p is disfavored with α-helical φ/ψ. Of the overlapping rotamers the average RMS angle distance is 5.8 for the -110/130 φ/ψ bin and 6.1 for the -60/-40 φ/ψ bin. χ1 rotamer angles cluster well around -60, 60, and 180.

Supplemental Figure 17

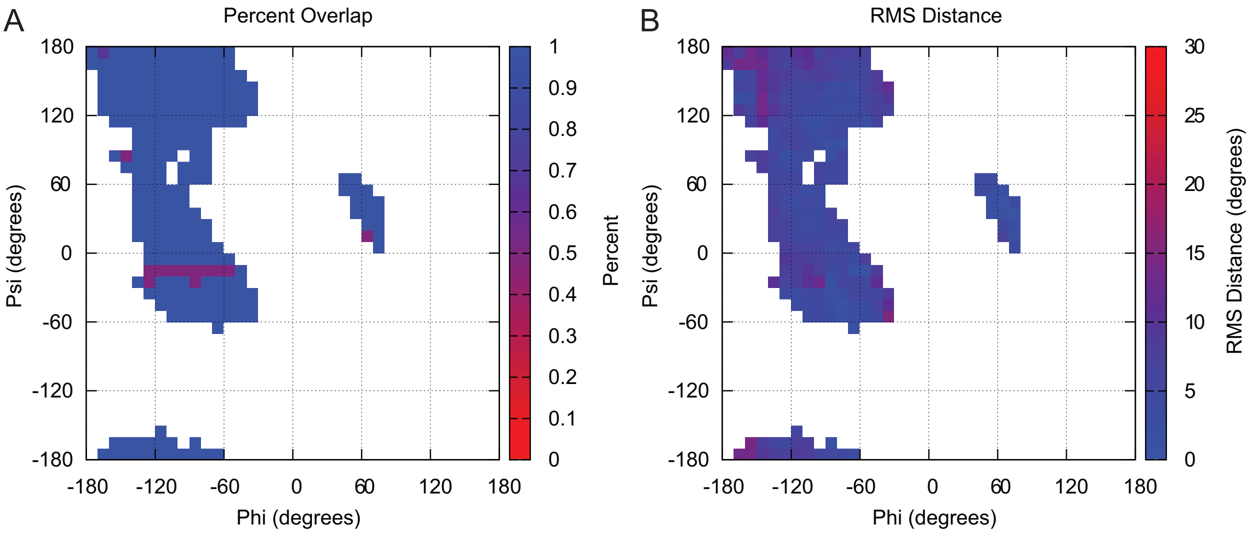

Comparing the percent of overlapping rotamer bins (A) and the RMS distance between overlapping bins (B) between the Dunbrack rotamer library and the rotamers predicted by the MakeRotLib protocol for cystine.

Glutamine

Glutamine has 3 χ angles with 3 χ1 rotamer wells (mpt), 3 χ2 rotamer wells (mpt), 4 χ3 rotamer wells (-120/0, -80/-40, 0/45, 80,120). At the -110/130 φ/ψ bin the top 13 rotamers capture 95% of the Dunbrack rotamers and 96% of the MakeRotLib rotamers, while at the -60/40 φ/ψ bin the top 16 rotamers capture 95% of the Dunbrack rotamers and 98% of the MakeRotLib rotamers. Overlap for the -110/130 φ/ψ bin is 69% while overlap for the -60/-40 φ/ψ bin is 75%. As with asparagine the χ3 dihedral is seen adopting angles that span a full rotation. Dunbrack χ3 angles cluster differently depending on the χ2. When χ2 is m or p the χ3 angles cluster at -120, -40, 40, and 120, when the χ2 is t the χ3 angles cluster at -70, 0, 70, 180. MakeRotLib χ3 angles cluster differently depending on the χ2. When χ2 is m or p the χ3 angles cluster at -100, -60, 0, and 100, when the χ2 is t the χ3 angles cluster at -110, 20, 60, and 110. The

MakeRotLib protocol does not find all the rotamers with a χ3 of 0 because they are wide and have large standard deviations, this brings the overlap down. Of the overlapping rotamers the average RMS angle distance is 15 for the -110/130 ϕ/ψ bin and 16.4 for the -60/-40 ϕ/ψ bin. The standard deviations reported by the Dunbrack library are often 20 degrees or more indicating that the MakeRotLib protocol is finding the correct rotamer wells but that the minimum of well differs between the two methods. χ1-2 rotamer angles cluster well around -60, 60, and 180.

Supplemental Figure 18

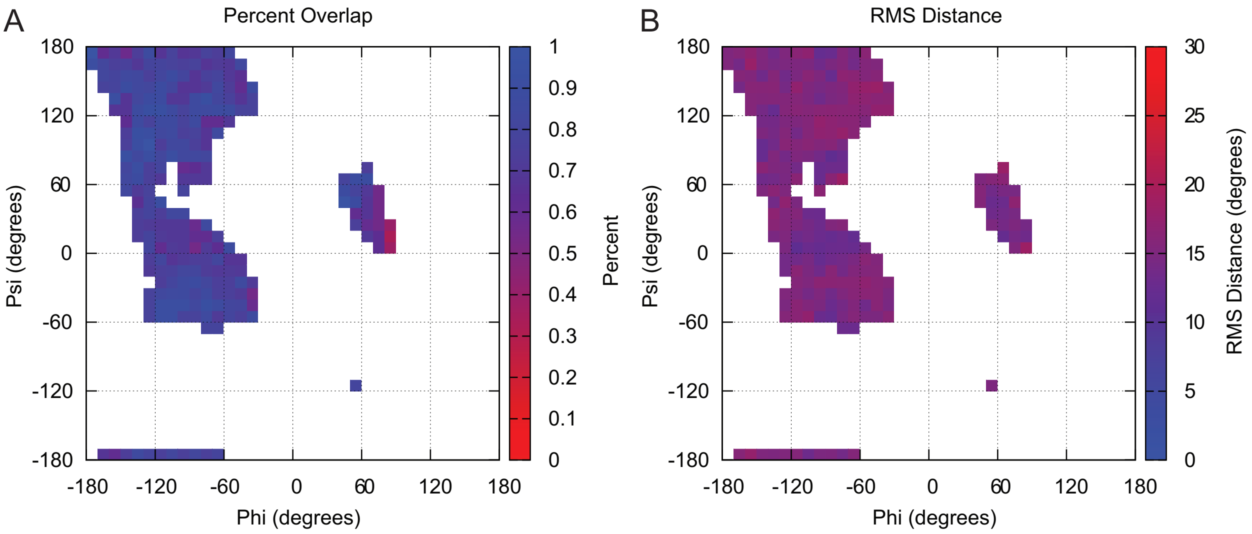

Comparing the percent of overlapping rotamer bins (A) and the RMS distance between overlapping bins (B) between the Dunbrack rotamer library and the rotamers predicted by the MakeRotLib protocol for glutamine.

Glutamic acid

Glutamic acid has 3 χ angles with 3 χ1 rotamer wells (mpt), 3 χ2 rotamer wells (mpt), 3 χ3 rotamer wells (centered on -60, 0, and 60), for a total of 27 rotamers. At the -110/130 ϕ/ψ bin the top 12 rotamers capture 95% of the Dunbrack rotamers and 97% of the MakeRotLib rotamers, while at the -60/40 ϕ/ψ bin the top 14 rotamers capture 95% of the Dunbrack rotamers and 99% of the MakeRotLib rotamers. Overlap for the -110/130 ϕ/ψ bin is 75% while overlap for the -60/-40 ϕ/ψ bin is 77%. As with aspartic acid, the MakeRotLib protocol finds rotamers with χ3 of -70, 0 and 70 but the 0 rotamer well is

considered to be higher energy by the MakeRotLib protocol and decreases the percent overlap. Of the overlapping rotamers the average RMS angle distance is 6.9 for the -110/130 ϕ/ψ bin and 10.0 for the -60/-40 ϕ/ψ bin. χ1-2 rotamer angles cluster well around -60, 60, and 180.

Supplemental Figure 19

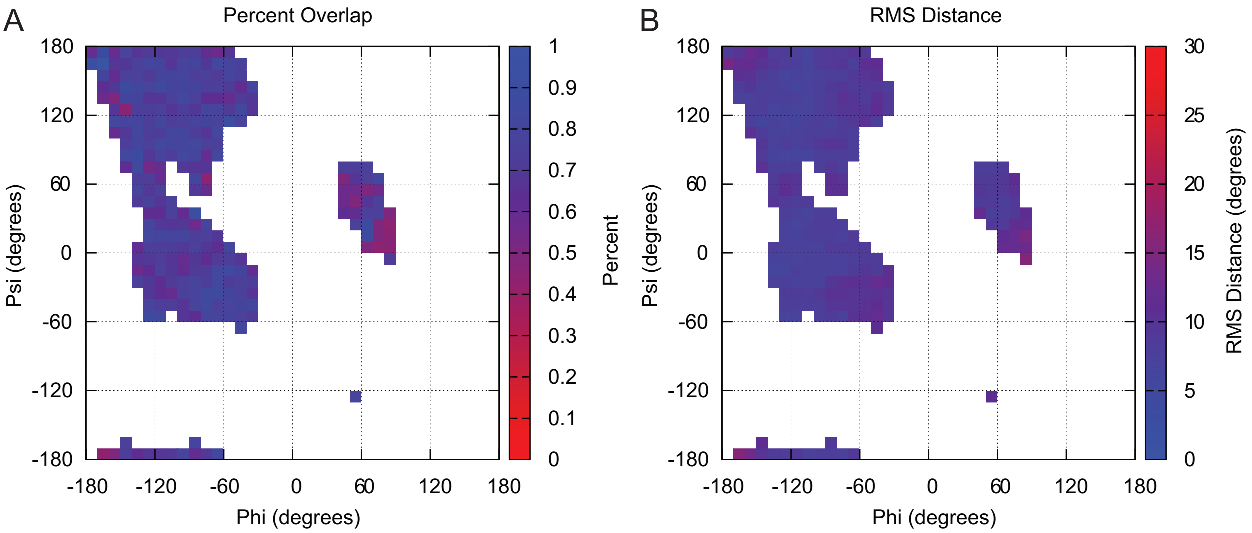

Comparing the percent of overlapping rotamer bins (A) and the RMS distance between overlapping bins (B) between the Dunbrack rotamer library and the rotamers predicted by the MakeRotLib protocol for glutamic acid.

Histidine

Histidine has 2 χ angles with 3 χ1 rotamer wells (mpt), 2 χ2 rotamer wells (centered on -90, 90, and 180), for a total of 9 rotamers. At the -110/130 ϕ/ψ bin the top 7 rotamers capture 100% of the Dunbrack rotamers and 98% of the MakeRotLib rotamers, while at the -60/40 ϕ/ψ bin the top 7 rotamers capture 99% of the Dunbrack rotamers and 98% of the MakeRotLib rotamers. Overlap for the -110/130 ϕ/ψ bin is 86% while overlap for the -60/-40 ϕ/ψ bin is 86%. The m180 and t180 rotamers are significantly populated in the Dunbrack rotamer library however the MakeRotLib protocol does not find them to be rotamers. The standard deviations for the rotamer are large with the βmt rotamer 32.4, the βtt rotamer at 29.4, the αmt rotamer at 21.7, and the αtt rotamer at 26.6. The absence of these rotamers lowers the overlap. Of the overlapping rotamers the average RMS angle distance is 13.8 for the -110/130 ϕ/ψ bin

and 12.6 for the -60/-40 ϕ/ψ bin. χ1 rotamer angles all cluster well around the expected -60, 60, and 180. χ2 rotamer angles all cluster well around the expected -65, 65. The Dunbrack library clusters closer to -80, 80 and is the reason for the overall RMS angle distance of 12.1 and the lowest 7.9. The imidazol ring of histidine can occupy a wide range of angles without large clashes as evidenced by the higher than average standard deviations of the Dunbrack library and the MakeRotLib protocol.

Supplemental Figure 20

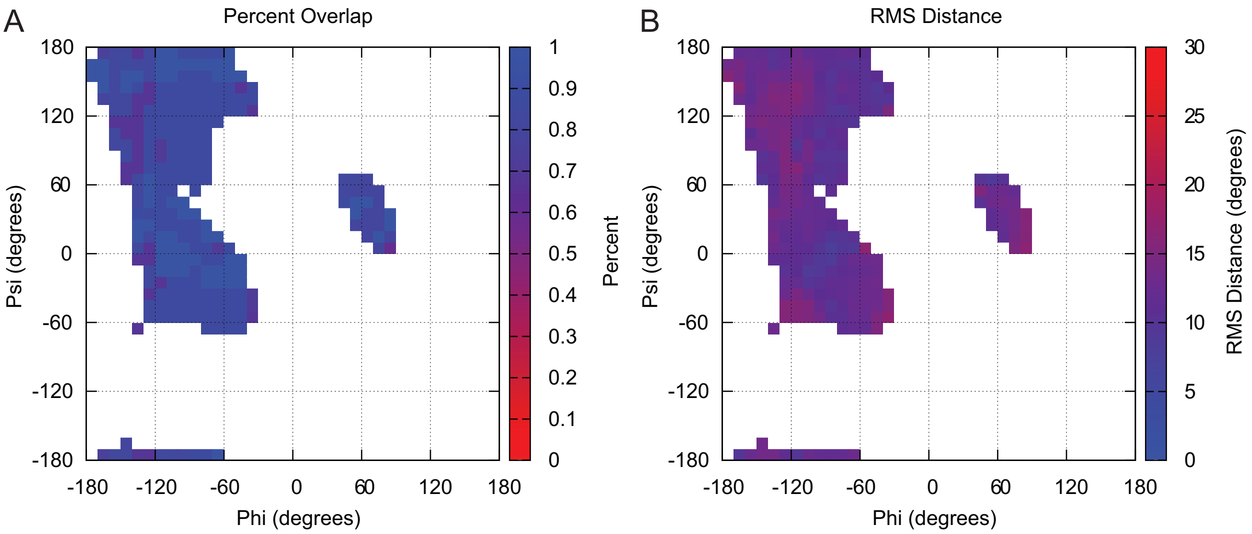

Comparing the percent of overlapping rotamer bins (A) and the RMS distance between overlapping bins (B) between the Dunbrack rotamer library and the rotamers predicted by the MakeRotLib protocol for histidine.

Isoleucine

Isoleucine has 2 χ angles with 3 χ1 rotamer wells (mpt) and 3 χ1 rotamer wells (mpt). At the -110/130 ϕ/ψ bin the top 4 rotamers capture 98% of the Dunbrack rotamers and 95% of the MakeRotLib rotamers, while at the -60/40 ϕ/ψ bin the top 4 rotamers capture 98% of the Dunbrack rotamers and 97% of the MakeRotLib rotamers. Overlap for the -110/130 ϕ/ψ bin is 50% while overlap for the -60/-40 ϕ/ψ bin is 50%. For the -110/130 ϕ/ψ bin and for the -60/-40 ϕ/ψ bin both methods favor the mm and the mt rotamers more than 80%. The overlap between top rotamers for both bins is 50% because of differences in the third and forth rotamers. Dunbrack prefers tt and mp, while the MakeRotLib protocol prefers pt and

tp for β-strand bin. Dunbrack prefers tt and tp, while the MakeRotLib protocol prefers pt and mp for α-helical bin. For the α-helical bin the results are unexpected as Renfrew et al [3] have shown that in the context of a dipeptide, the distribution of rotamer probabilities is more even and that the preferred rotamers are tp, tt, pt, mt, and mm . Of the overlapping rotamers the average RMS angle distance is 8.6 for the -110/130 ϕ/ψ bin and 15.6 for the -60/-40 ϕ/ψ bin. χ1-2 rotamer angles cluster well around -60, 60, and 180. However the -60/-40 ϕ/ψ bin the tp is skewed to 40, 160.

Supplemental Figure 21

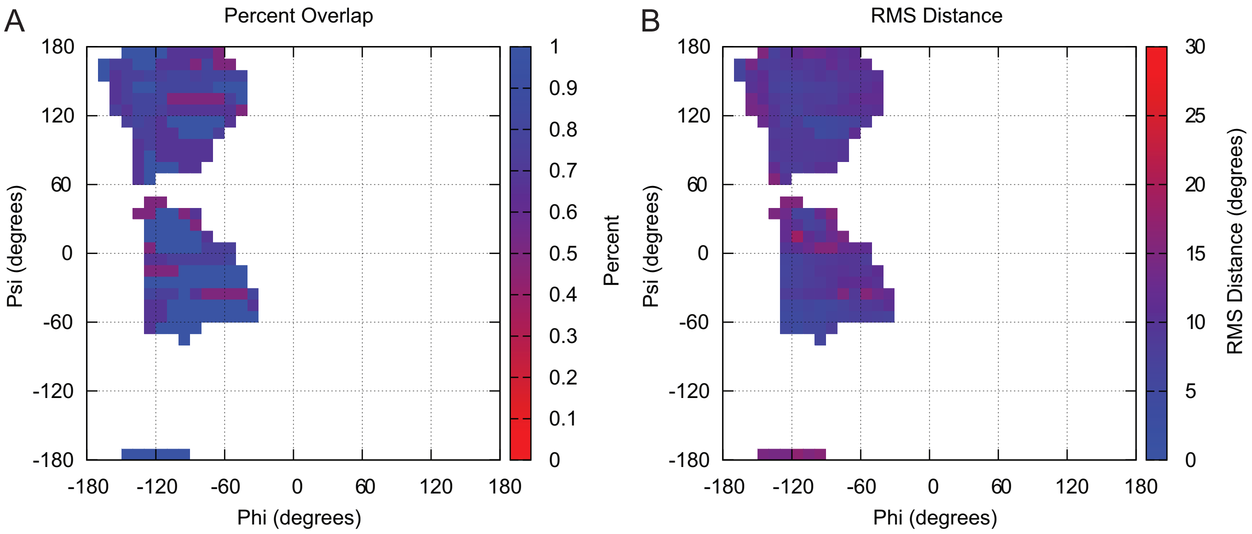

Comparing the percent of overlapping rotamer bins (A) and the RMS distance between overlapping bins (B) between the Dunbrack rotamer library and the rotamers predicted by the MakeRotLib protocol for isoleucine.

Lysine

Lysine has 4 χ angles with 3 χ1 rotamer wells (mpt), 3 χ2 rotamer wells (mpt), 3 χ3 rotamer wells (mpt) and 3 χ4 rotamer wells (mpt) for a total of 81 rotamers. At the -110/130 ϕ/ψ bin the top 24 rotamers capture 95% of the Dunbrack rotamers and 98% of the MakeRotLib rotamers, while at the At the -60/40 ϕ/ψ bin the top 26 rotamers capture 95% of the Dunbrack rotamers and 95% of the MakeRotLib rotamers. Overlap for the -110/130 ϕ/ψ bin is 92% while overlap for the -60/-40 ϕ/ψ bin is 85%. Low overlap probabilities occur in the regions where the Dunbrack library has low counts near the boundary of

the 3/10-helical region. Of the overlapping rotamers the average RMS angle distance is 5.7 for the -110/130 ϕ/ψ bin and 6.5 for the -60/-40 ϕ/ψ bin. χ1-4 rotamer angles all cluster well around the expected -60, 60, and 180.

Supplemental Figure 22

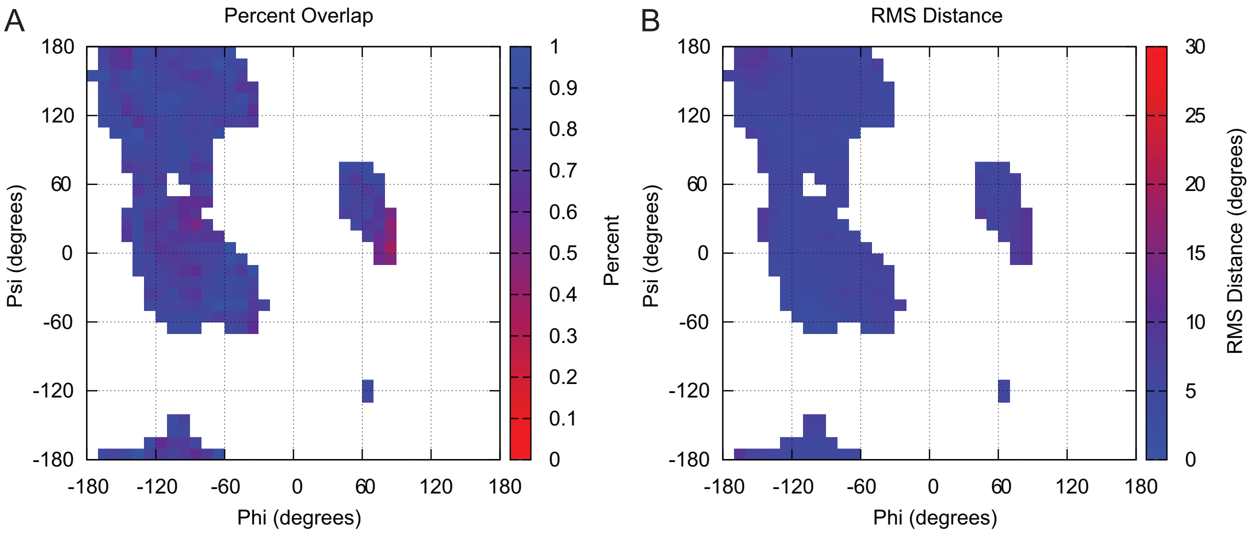

Comparing the percent of overlapping rotamer bins (A) and the RMS distance between overlapping bins (B) between the Dunbrack rotamer library and the rotamers predicted by the MakeRotLib protocol for lysine.

Methionine

Methionine has 3 χ angles with 3 χ1 rotamer wells (mpt), 3 χ2 rotamer wells (mpt), and 3 χ3 rotamer wells (mpt). At the -110/130 ϕ/ψ bin the top 12 rotamers capture 96% of the Dunbrack rotamers and 96% of the MakeRotLib rotamers, while at the -60/40 ϕ/ψ bin the top 11 rotamers capture 95% of the Dunbrack rotamers and 96% of the MakeRotLib rotamers. Overlap for the -110/130 ϕ/ψ bin is 92% while overlap for the -60/-40 ϕ/ψ bin is 91%. Of the overlapping rotamers the average RMS angle distance is 5.5 for the -110/130 ϕ/ψ bin and 5.5 for the -60/-40 ϕ/ψ bin. χ1-2 rotamer angles all cluster well around the expected -60, 60, and 180. χ3 angles cluster around -70, 70, and 180 with the exception of the mmp rotamer in the α-helical ϕ/ψ where the χ3 is 105. This value is consistent with the Dunbrack library for that rotamer.

Supplemental Figure 23

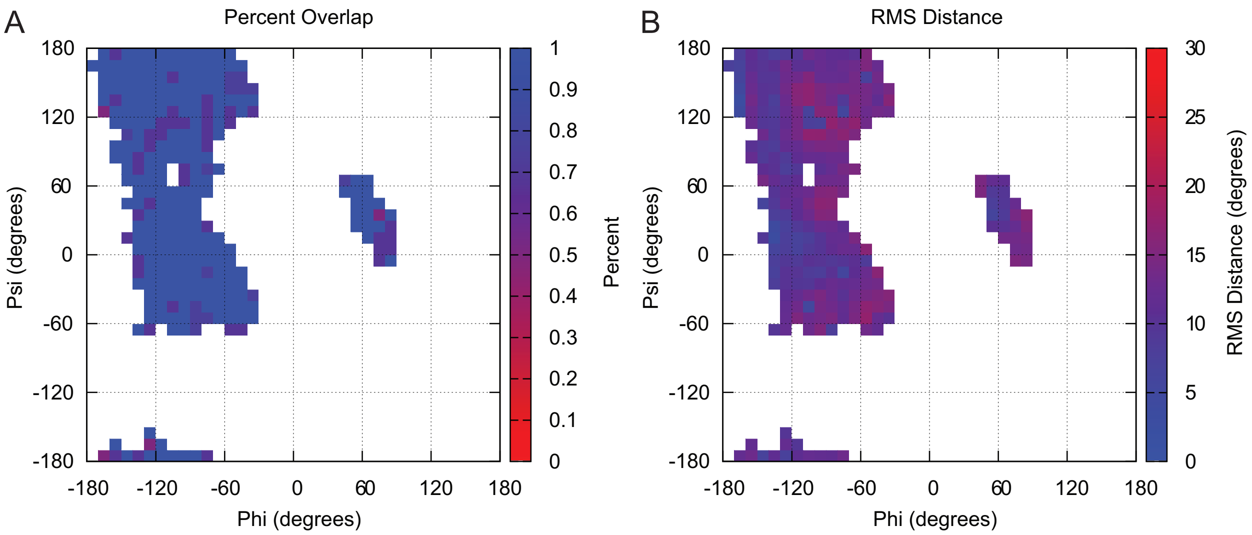

Comparing the percent of overlapping rotamer bins (A) and the RMS distance between overlapping bins (B) between the Dunbrack rotamer library and the rotamers predicted by the MakeRotLib protocol for methionine.

Serine

Serine has 1 χ angle with 3 χ1 rotamer wells (mpt). At the -110/130 ϕ/ψ bin the top 3 rotamers capture 100% of the Dunbrack rotamers and 100% of the MakeRotLib rotamers, while at the -60/40 ϕ/ψ bin the top 3 rotamers capture 100% of the Dunbrack rotamers and 100% of the MakeRotLib rotamers. Overlap for the -110/130 ϕ/ψ bin is 100% while overlap for the -60/-40 ϕ/ψ bin is 100%. For the -110/130 ϕ/ψ bin the Dunbrack and MakeRotLib protocol differ significantly in their preferred rotamers. Dunbrack orders rotamers t,m, and p at 46%, 45%, and 9% respectively. While MakeRotLib orders the rotamers m,p and t at 79%, 11%, and 10% respectively. For the -60/40 ϕ/ψ bin the MakeRotlib protocol only favors the m rotamer, at 98%. The Dunbrack is much more evenly distributed which allows for the comparison of all rotamers and is the reason for the 100% overlap. The narrow distribution is the result of improper ideal coordinates that place the hydroxyl hydrogen in a position to clash with the backbone. Of the overlapping rotamers the average RMS angle distance is 5.5 for the -110/130 ϕ/ψ bin and 8.4 for the -60/-40 ϕ/ψ bin.

Supplemental Figure 24

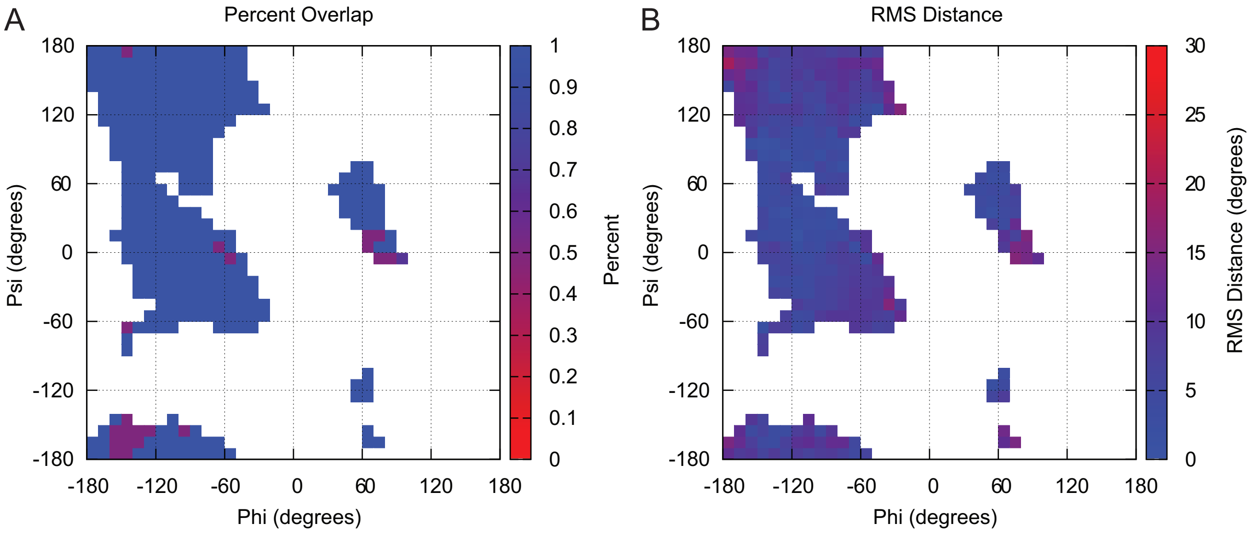

Comparing the percent of overlapping rotamer bins (A) and the RMS distance between overlapping bins (B) between the Dunbrack rotamer library and the rotamers predicted by the MakeRotLib protocol for serine.

Threonine

Threonine has 1 χ angle with 3 $\chi 1$ rotamer wells (mpt). At the -110/130 ϕ/ψ bin the top 2 rotamers capture 98% of the Dunbrack rotamers and 100% of the MakeRotLib rotamers, while at the -60/40 ϕ/ψ bin the top 2 rotamers capture 99% of the Dunbrack rotamers and 100% of the MakeRotLib rotamers. Overlap for the -110/130 ϕ/ψ bin is 100% while overlap for the -60/-40 ϕ/ψ bin is 100%. Three ϕ/ψ bins have zero overlap probabilities: 80/-10, 70/170, 70/-10. Both Dunbrack and the MakeRotLib protocol have very strongly prefer (>95%) a different rotamers and the overlap is therefore 0%. The bins are on the borders of the 3/10 helical region and have very low counts. As with serine the p rotamer is significantly populated in the α-helical ϕ/ψ bin. Of the overlapping rotamers the average RMS angle distance is 8.4 for the -110/130 ϕ/ψ bin and 9.4 for the -60/-40 ϕ/ψ bin. $\chi 1$ rotamer angles all cluster well around the expected -60, 60, and 180.

Supplemental Figure 25

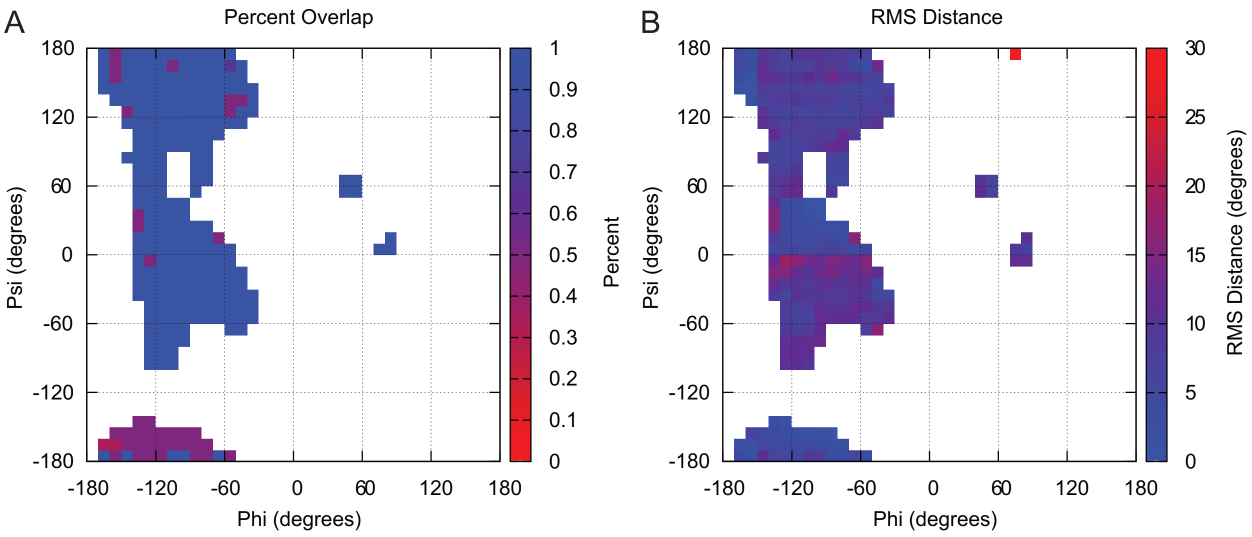

Comparing the percent of overlapping rotamer bins (A) and the RMS distance between overlapping bins (B) between the Dunbrack rotamer library and the rotamers predicted by the MakeRotLib protocol for threonine.

Tryptophan

Tryptophan has 2 χ angles with 3 χ1 rotamer wells (mpt), and 3 χ2 rotamer wells (centered on -120, 0, 120). At the -110/130 ϕ/ψ bin the top 5 rotamers capture 99% of the Dunbrack rotamers and 98% of the MakeRotLib rotamers, while at the -60/40 ϕ/ψ bin the top 6 rotamers capture 98% of the Dunbrack rotamers and 100% of the MakeRotLib rotamers. Overlap for the -110/130 ϕ/ψ bin is 80% while overlap for the -60/-40 ϕ/ψ bin is 67%. At both α-helical and β-strand ϕ and ψ the MakeRotlib protocol does not find the m0 rotamer. The standard deviation in the Dunbrack library is given as 23.4 degrees for -110/130 ϕ/ψ bin and 22.2 degrees for the -60/-40 ϕ/ψ bin suggesting that it is quite wide. The absence of this rotamer lowers the overlap in these regions. Additionally for the -60/-40 ϕ/ψ bin the Dunbrack rotamer library gives the m-90 rotamer a probability of 1.6% and the rotamer library of Lovell et al. gives the same rotamer a 0% while it is the most favorable rotamer for the MakeRotLib protocol at 49%. Constructing this rotamer in the context of a dipeptide with helical ϕ and ψ shows no major clashes. However, if it is constructed in the context of a α-helix, there is a large clash with the neighbor side chain in the helix. Indeed the m-90 rotamer in the Dunbrack library has a significantly shifted angles χ1 = -90,

$\chi2$ = -120. The large RMS angle distance is 30.99 for this rotamer placing it out of the cutoff range and decreasing the overlap in the -60/-40 ϕ/ψ bin. Of the overlapping rotamers the average RMS angle distance is 9.9 for the -110/130 ϕ/ψ bin and 8.4 for the -60/-40 ϕ/ψ bin. $\chi1$ rotamer angles all cluster well around the expected -60, 60, and 180. $\chi2$ rotamer angles all cluster well around the expected -90 and 90 but are missing the 0 rotamer.

Supplemental Figure 26

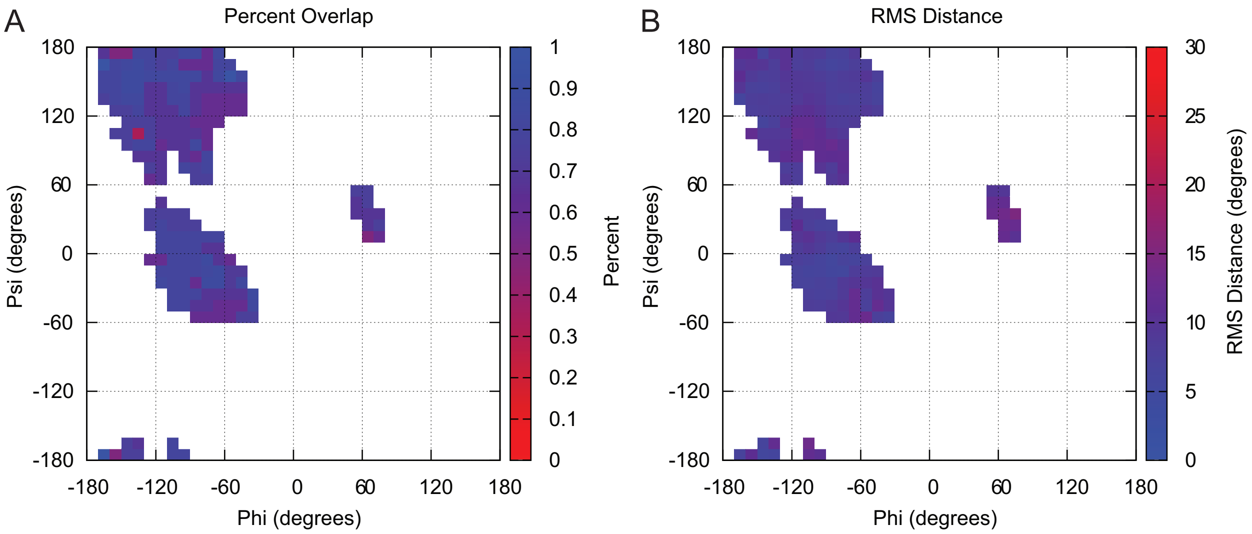

Comparing the percent of overlapping rotamer bins (A) and the RMS distance between overlapping bins (B) between the Dunbrack rotamer library and the rotamers predicted by the MakeRotLib protocol for tryptophan.

Tyrosine

Tyrosine has 2 χ angles with 3 $\chi1$ rotamer wells (mpt), 2 $\chi2$ rotamer wells (centered on 90 and 0) for a total of 6 rotamers. At the -110/130 ϕ/ψ bin the top 4 rotamers capture 99% of the Dunbrack rotamers and 99% of the MakeRotLib rotamers, while at the -60/40 ϕ/ψ bin the top 4 rotamers capture 98% of the Dunbrack rotamers and 98% of the MakeRotLib rotamers. Overlap for the -110/130 ϕ/ψ bin is 100% while overlap for the -60/-40 ϕ/ψ bin is 100%. As with phenylalanine, $\chi2$ rotamer wells centered on 0 and are typically at 20 and -20 are shifted to -40. This could potentially be the result of bond angle deviations

that would not be taken into account by the MakeRotLib protocol. Of the overlapping rotamers the average RMS angle distance is 15.2 for the -110/130 ϕ/ψ bin and 13.3 for the -60/-40 ϕ/ψ bin.

Supplemental Figure 27

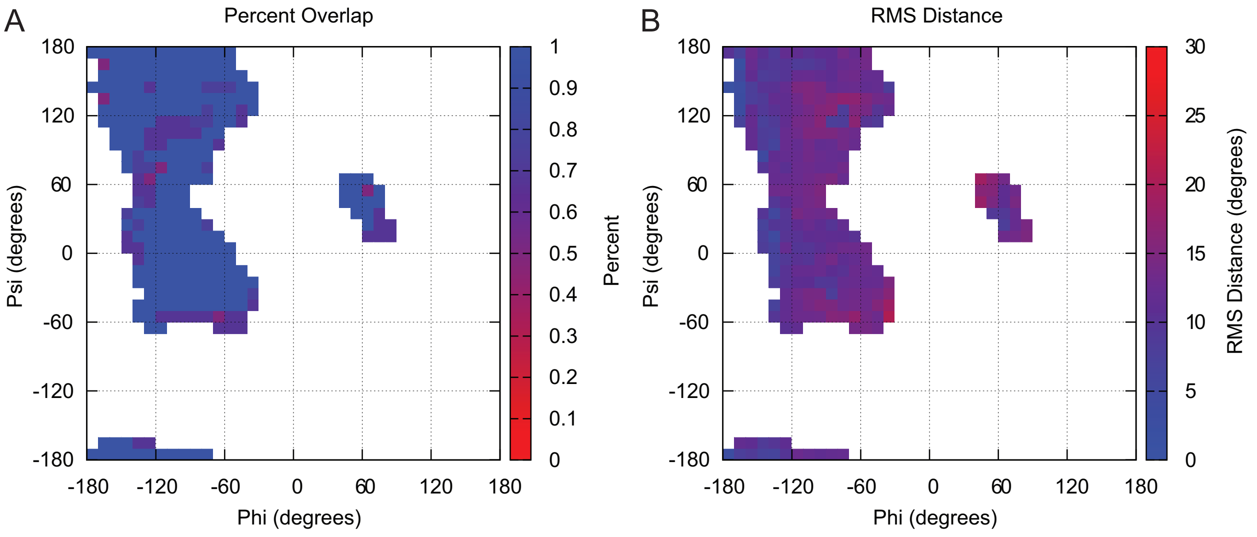

Comparing the percent of overlapping rotamer bins (A) and the RMS distance between overlapping bins (B) between the Dunbrack rotamer library and the rotamers predicted by the MakeRotLib protocol for tyrosine.

Valine

Valine has 1 χ angles with 3 χ1 rotamer wells (mpt). At the -110/130 ϕ/ψ bin the top 2 rotamers capture 97% of the Dunbrack rotamers and 100% of the MakeRotLib rotamers, while at the -60/40 ϕ/ψ bin the top 2 rotamers capture 97% of the Dunbrack rotamers and 100% of the MakeRotLib rotamers. Overlap for the -110/130 ϕ/ψ bin is 50% while overlap for the -60/-40 ϕ/ψ bin is 50%. Looking at the over overlap performance the results are good with the MakeRotLib protocol finding 88% of the Dunbrack rotamers. There is a distinct ψ dependence shown in the banding pattern in figure 2. In the β-strand region the preferred rotamer shifts from m to t between the -150 and-160 ψ bins. The shift occurs between the -140 and -150 ψ bins for the MakeRotLib protocol. For the -60/-40 ϕ/ψ bin the Dunbrack rotamer library both strongly prefer (>90%) the t rotamer. This result was unexpected since we have previously shown that when using a dipeptide model system and using a full molecular-mechanics force

field or high-level quantum mechanics to calculate the energy of a dipeptide free from long-range interactions that could bias the rotamer probabilities, the rotamer distribution for each rotamer well was approximately even for the MM and the QM [3]. Of the overlapping rotamers the average RMS angle distance is 10.5 for the -110/130 ϕ/ψ bin and 16.5 for the -60/-40 ϕ/ψ bin. $\chi 1$ rotamer angles cluster well around -60, 60, and 180. Both the MakeRotLib protocol and the Dunbrack rotamer library prefer the t rotamer for the α-helical region. The angle preferred by the MakeRotlib protocol for the t rotamer differs from the Dunbrack by 14 degrees which is higher than the average for all seen positions. This trend was also seen Renfrew et al. with the full CHARMM potential preferring a t rotamer with a value of ~190 degrees.

Supplemental Figure 28

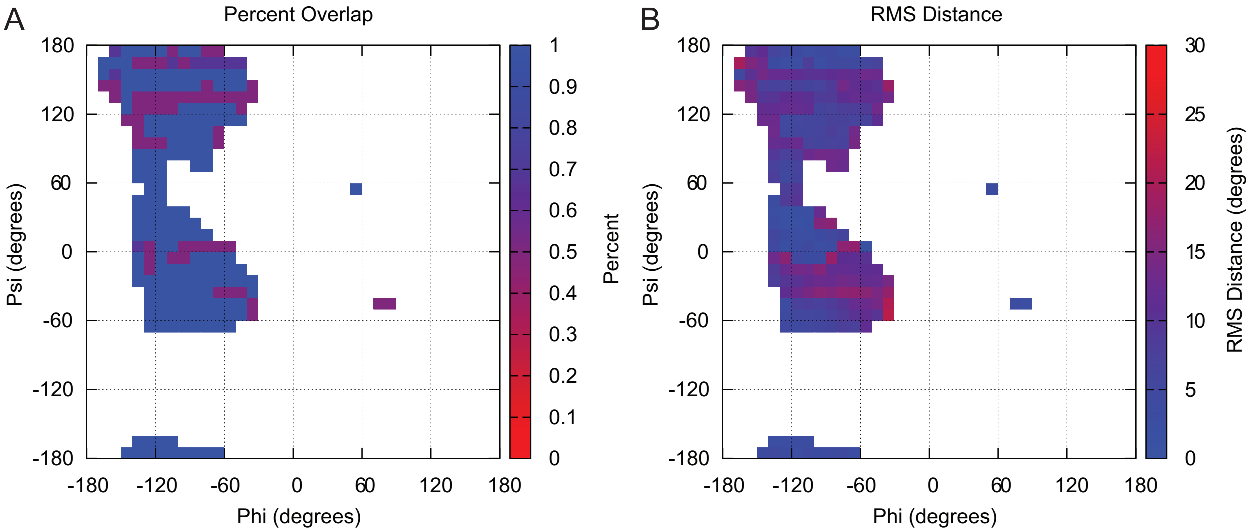

Comparing the percent of overlapping rotamer bins (A) and the RMS distance between overlapping bins (B) between the Dunbrack rotamer library and the rotamers predicted by the MakeRotLib protocol for valine.

Fluorescence Polarization Binding Assays

Supplemental Figure 29

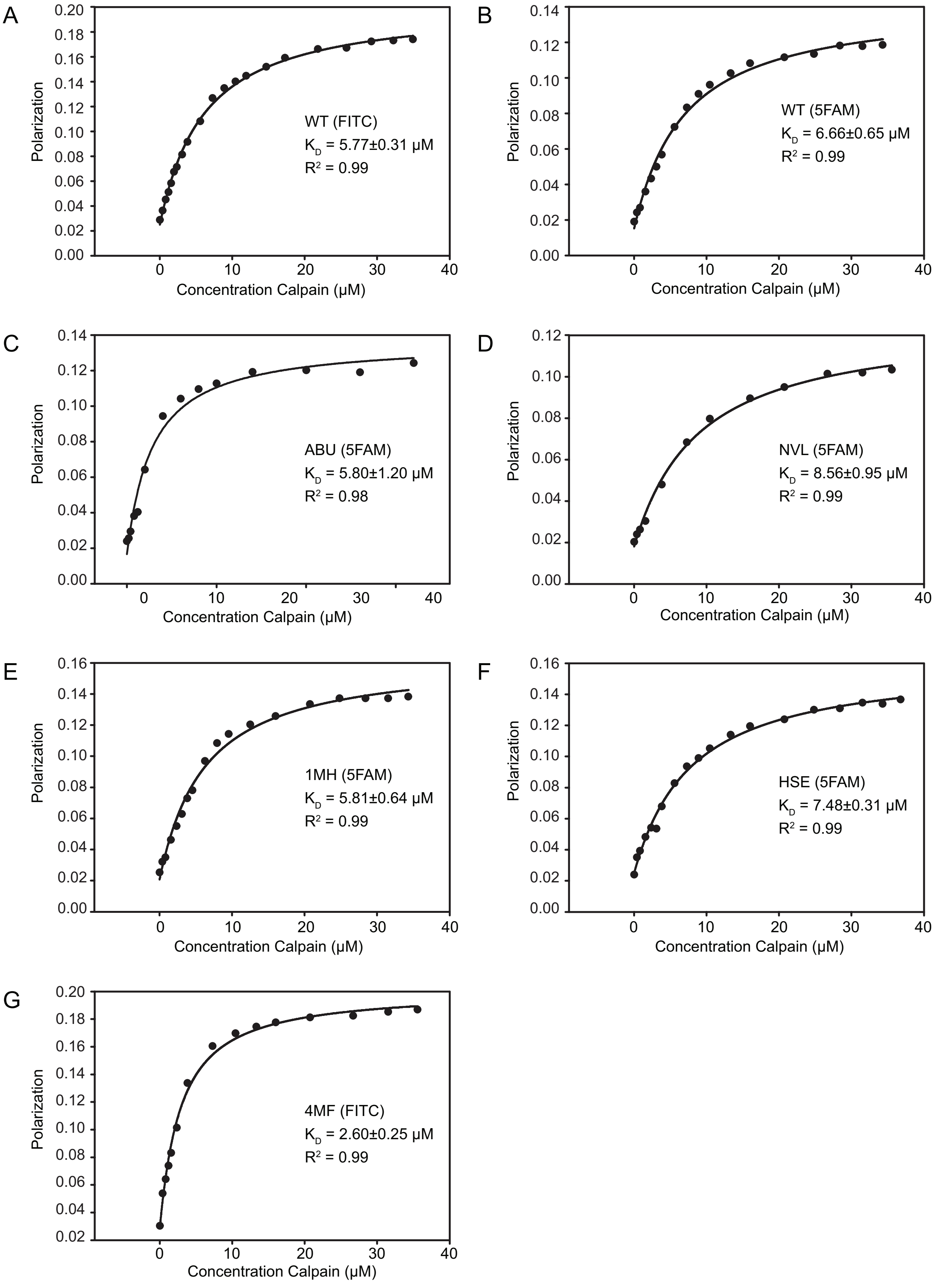

Fluorescence polarization binding curves for calpain and a peptide with wild-type calpastatin sequence labeled with a FITC dye (A) or 5FAM dye (B), and peptides with mutations of position 607 to amino

butyric acid (C) norvaline (D), position 609 to 1-methyl-histidine (E) and homoserine (F), and position 610 to 4-methyl-phenylalanine. Disassociation constants and correlation constants for fitting are shown inset.

Supplementary Bibliography

1. DeLano WL (2002) The Pymol Molecular Graphics System. Palo Alto, CA: DeLano Scientific.

2. Lovell SC, Word JM, Richardson JS, Richardson DC (2000) The penultimate rotamer library. Proteins: Structure, Function, and Bioinformatics 40: 389-408.

3. Renfrew PD, Butterfoss GL, Kuhlman B (2008) Using quantum mechanics to improve estimates of amino acid side chain rotamer energies. Proteins 71: 1637-1646.
