# Supplementary material for: Incorporation of Noncanonical Amino Acids into Rosetta and Use in Computational Protein-Peptide Interface Design
Source: PLoS One. 2012 Mar 14;7(3):e32637. doi: 10.1371/journal.pone.0032637 (PMC3303795; doi:10.1371/journal.pone.0032637)
Supplement: Supporting Information S2 — “Protocol capture” that includes descriptions, instructions, command lines and processing scripts to reproduce the NCAA parameter files, explicit unfolded state energies, and rotamer libraries. Additionally the protocol capture includes descriptions, instructions, command lines and processing scripts used to redesign the NCAA peptides. (GZ) [file pone.0032637.s002.gz › using_ncaas_protein_peptide_interface_design/HowToMakeResidueTypeParamFiles/openbabel/openbabel-2.2.0.tar.gz!/openbabel-2.2.0/doc/babel.html]

babel

Table of Contents

## Name

**babel** -- a converter for chemistry and molecular modeling data files

## **Synopsis**

**babel** [**-H** *help-options*]  
**babel** [*OPTIONS*] [**-i** *input-type*] *infile* [**-o** *output-type*] *outfile*

## **Description**

Babel is a cross-platform program designed to interconvert between many
file formats used in molecular modeling and computational chemistry and
related areas.

Open Babel is also a complete programmers toolkit for developing chemistry
software. For more information, se the Open Babel web pages
<**http://openbabel.org/**>.

## **Options**

If only input and ouput files are given, Open Babel will guess the file
type from the filename extension.

**-a** *options*
:   Format-specific input options. See **-H** *format-ID* for options allowed
    by a particular format

**--addtotitle**
:   Append text to the current molecule title

**--addformula**
:   Append the molecular formula after the current molecule title

**-b**
:   Convert dative bonds: e.g., [N+]([O-])=O to N(=O)=O

**-c**
:   Center atomic coordinates at (0,0,0)

**-C**
:   Combine molecules in first file with others having the same name

**-e**
:   Continue after errors

**-d**
:   Delete Hydrogens

**---errorlevel** *2*
:   Filter the level of errors and warnings displayed:
    1 = critical errors only
    2 = include warnings too (default)
    3 = include informational messages too
    4 = include “audit log” messages of changes to data
    5 = include debugging messages too

**-f** *#*
:   For multiple entry input, start import with molecule # as the first
    entry

**-F**
:   Output the available fingerprint types

**-h**
:   Add hydrogens

**-H**
:   Output usage information

**-H** *format-ID*
:   Output formatting information and options for format specified

**-Hall**
:   Output formatting information and options for all formats

**-i<format-ID>**
:   Specifies input format, see below for the available formats

**-j**


**--join**
:   Join all input molecules into a single output molecule entry

**-k**
:   Translate computational chemistry modeling keywords (e.g., GAMESS
    and Gaussian)

**-m**
:   Produce multiple output files, to allow:

**-**
:   Splitting one input file - put each molecule into consecutively
    numbered output files

**-**
:   Batch conversion - convert each of multiple input files
    into a specified output format

**-l** *#*
:   For multiple entry input, stop import with molecule # as the last
    entry

**-o** *format-ID*
:   Specifies output format, see below for the available formats

**-p**
:   Add Hydrogens appropriate for pH (use transforms in phmodel.txt)

**--property**
:   Add or replace a property (e.g., in an MDL SD file)

**-s** *SMARTS*
:   Convert only molecules matching the SMARTS pattern specified

**--separate**
:   Separate disconnected fragments into individual molecular records

**-t**
:   All input files describe a single molecule

**--title** *title*
:   Add or replace molecular title

**-x** *options*
:   Format-specific output options. See **-H** *format-ID* for options
    allowed by a particular format

**-v** *SMARTS*
:   Convert only molecules **NOT** matching SMARTS pattern specified

**-V**
:   Output version number and exit

**-z**
:   Compress the output with gzip

## **File** **Formats**

The following formats are currently supported by Open Babel:

- acr -- Carine ASCI Crystal
- alc -- Alchemy format
- arc -- Accelrys/MSI Biosym/Insight II CAR format [Read-only]
- bgf -- MSI BGF format
- box -- Dock 3.5 Box format
- bs -- Ball and Stick format
- c3d1 -- Chem3D Cartesian 1 format
- c3d2 -- Chem3D Cartesian 2 format
- caccrt -- Cacao Cartesian format
- cache -- CAChe MolStruct format [Write-only]
- cacint -- Cacao Internal format [Write-only]
- can -- Canonical SMILES format
- car -- Accelrys/MSI Biosym/Insight II CAR format [Read-only]
- ccc -- CCC format [Read-only]
- cdx -- ChemDraw binary format [Read-only]
- cdxml -- ChemDraw CDXML format
- cht -- Chemtool format [Write-only]
- cif -- Crystallographic Information File
- cml -- Chemical Markup Language
- cmlr -- CML Reaction format
- com -- Gaussian 98/03 Cartesian Input [Write-only]
- copy -- Copies raw text [Write-only]
- crk2d -- Chemical Resource Kit 2D diagram format
- crk3d -- Chemical Resource Kit 3D format
- csr -- Accelrys/MSI Quanta CSR format [Write-only]
- cssr -- CSD CSSR format [Write-only]
- ct -- ChemDraw Connection Table format
- dmol -- DMol3 coordinates format
- ent -- Protein Data Bank format
- fa -- FASTA format [Write-only]
- fasta -- FASTA format [Write-only]
- fch -- Gaussian formatted checkpoint file format [Read-only]
- fchk -- Gaussian formatted checkpoint file format [Read-only]
- fck -- Gaussian formatted checkpoint file format [Read-only]
- feat -- Feature format
- fh -- Fenske-Hall Z-Matrix format [Write-only]
- fix -- SMILES FIX format [Write-only]
- fpt -- Fingerprint format [Write-only]
- fract -- Free Form Fractional format
- fs -- Open Babel FastSearching database
- fsa -- FASTA format [Write-only]
- g03 -- Gaussian 98/03 Output [Read-only]
- g98 -- Gaussian 98/03 Output [Read-only]
- gam -- GAMESS Output [Read-only]
- gamin -- GAMESS Input [Write-only]
- gamout -- GAMESS Output [Read-only]
- gau -- Gaussian 98/03 Cartesian Input [Write-only]
- gjc -- Gaussian 98/03 Cartesian Input [Write-only]
- gjf -- Gaussian 98/03 Cartesian Input [Write-only]
- gpr -- Ghemical format
- gr96 -- GROMOS96 format [Write-only]
- hin -- HyperChem HIN format
- inchi -- IUPAC InChI [Write-only]
- inp -- GAMESS Input [Write-only]
- ins -- ShelX format [Read-only]
- jin -- Jaguar input format [Write-only]
- jout -- Jaguar output format [Read-only]
- mdl -- MDL MOL format
- mmd -- MacroModel format
- mmod -- MacroModel format
- mol -- MDL MOL format
- mol2 -- Sybyl Mol2 format
- molreport -- Open Babel molecule report [Write-only]
- moo -- MOPAC Output format [Read-only]
- mop -- MOPAC Cartesian format
- mopcrt -- MOPAC Cartesian format
- mopin -- MOPAC Internal
- mopout -- MOPAC Output format [Read-only]
- mpc -- MOPAC Cartesian format
- mpd -- Sybyl descriptor format [Write-only]
- mpqc -- MPQC output format [Read-only]
- mpqcin -- MPQC simplified input format [Write-only]
- nw -- NWChem input format [Write-only]
- nwo -- NWChem output format [Read-only]
- pc -- PubChem format [Read-only]
- pcm -- PCModel format
- pdb -- Protein Data Bank format
- pov -- POV-Ray input format [Write-only]
- pqs -- Parallel Quantum Solutions format
- prep -- Amber Prep format [Read-only]
- qcin -- Q-Chem input format [Write-only]
- qcout -- Q-Chem output format [Read-only]
- report -- Open Babel report format [Write-only]
- res -- ShelX format [Read-only]
- rxn -- MDL RXN format
- sd -- MDL MOL format
- sdf -- MDL MOL format
- smi -- SMILES format
- sy2 -- Sybyl Mol2 format
- tdd -- Thermo format
- test -- Test format [Write-only]
- therm -- Thermo format
- tmol -- TurboMole Coordinate format
- txyz -- Tinker MM2 format [Write-only]
- unixyz -- UniChem XYZ format
- vmol -- ViewMol format
- xed -- XED format [Write-only]
- xml -- General XML format [Read-only]
- xyz -- XYZ cartesian coordinates format
- yob -- YASARA.org YOB format
- zin -- ZINDO input format [Write-only]

## **Format** **Options**

Individual file formats may have additional formatting options.

Input format options are preceded by ‘a’, e.g. `-as`

Output format options are preceded by ‘x’, e.g. `-xn`

For further specific information and options, use `-H<format-type>`
e.g., `-Hcml`

## **Examples**

Standard conversion:  

```
babel -ixyz ethanol.xyz -opdb ethanol.pdb
```

Conversion from a SMI file in STDIN to a Mol2 file written to STDOUT:  

```
babel -ismi -omol2
```

Split a multi-molecule file into new1.smi, new2.smi, etc.:  

```
babel infile.mol new.smi -m
```

## **See** **Also**

obenergy(1)
, obfit(1)
, obgrep(1)
, obminimize(1)
, obprop(1)
, obrotate(1)
, obrotamer(1)
.

The web pages for Open Babel can be found at:
<**http://openbabel.org/**>

More details on the formats supported by Open Babel can be found at:
<**http://openbabel.org/wiki/Formats**>

## **Authors**

A cast of many, including the currrent maintainers Geoff Hutchison, Chris
Morley, Michael Banck, and innumerable others who have contributed fixes
and additions. For more contributors to Open Babel, see <**http://openbabel.org/wiki/THANKS**>

## **Copyright**

Copyright (C) 1998-2001 by OpenEye Scientific Software, Inc.  
Some portions Copyright (C) 2001-2007 by Geoffrey R. Hutchison

This program is free software; you can redistribute it and/or modify it
under the terms of the GNU General Public License as published by the
Free Software Foundation version 2 of the License.

This program is distributed in the hope that it will be useful, but
WITHOUT ANY WARRANTY; without even the implied warranty of MERCHANTABILITY
or FITNESS FOR A PARTICULAR PURPOSE. See the GNU General Public
License for more details.

---

**Table of Contents**

- Name
- Synopsis
- Description
- Options
- File Formats
- Format Options
- Examples
- See Also
- Authors
- Copyright
