# Supplementary material for: Incorporation of Noncanonical Amino Acids into Rosetta and Use in Computational Protein-Peptide Interface Design
Source: PLoS One. 2012 Mar 14;7(3):e32637. doi: 10.1371/journal.pone.0032637 (PMC3303795; doi:10.1371/journal.pone.0032637)
Supplement: Supporting Information S2 — “Protocol capture” that includes descriptions, instructions, command lines and processing scripts to reproduce the NCAA parameter files, explicit unfolded state energies, and rotamer libraries. Additionally the protocol capture includes descriptions, instructions, command lines and processing scripts used to redesign the NCAA peptides. (GZ) [file pone.0032637.s002.gz › using_ncaas_protein_peptide_interface_design/HowToMakeResidueTypeParamFiles/openbabel/openbabel-2.2.0.tar.gz!/openbabel-2.2.0/doc/FAQ.html]

Open Babel: Frequently Asked Questions


# FAQ

$Date: 2008-07-03 12:02:45 -0400 (Thu, 03 Jul 2008) $

## General:

### 1.1 What is Open Babel?

Put simply, Open Babel is a free, open-source version of the Babel
chemistry file translation program. Open Babel is a project designed to pick up where Babel left off, as
a cross-platform program and library designed to interconvert between
many file formats used in molecular modeling, computational
chemistry, and many related areas.

Open Babel includes two components, a command-line utility and a
C++ library. The command-line utility is intended to be used as a
replacement for the original babel program, to translate between
various chemical file formats. The C++ library includes all of the
file-translation code as well as a wide variety of utilities to
foster development of other open source scientific software.

### 1.2 How does this relate to BabelChat, BabelFish, Babel IM, etc. ...?

It doesn't. Not surprisingly, "babel" is used frequently in a lot of software names.

### 1.3 Is it Open Babel or OpenBabel?

Your choice. It's probably easier to call it Open Babel since
that's what it is--an open version of Babel. But if you like
one-word, mixed-case project names, then go for OpenBabel. In that
case, the space is just too small to be printed.

```
;-)
```

### 1.4 How does this relate to the original Babel and OELib, the "next" Babel?

The original Babel was written by Pat Walters and Matt Stahl, based
on the "convert" program by Ajay Shah, and is
still a remarkable application. Both Pat and Matt have moved on to
other work. The original Babel is hosted by Smog.com on a
Babel homepage, by the Computational Chemistry List (CCL) and of course by Open Babel at SourceForge.net

Along the way, the two original authors started a rewrite of Babel
into C++ they called OBabel, which was never really publicly
released. But Matt used some of these ideas in OELib, which was generously
released under the GNU GPL by his employer, OpenEye Software, and the last known
version of this OELib is still available from our file repository.

OpenEye decided that for their purposes, OELib needed a rewrite,
(now called OEChem
but this would be closed-source to include some advanced
algorithms. So the GPL'ed version of OELib would not be maintained.
Instead, the free version of OELib was renamed and has become "Open
Babel" with the blessing of Matt and other contributors.

Open Babel has evolved quite a lot in since 2001, when it was born.

### 1.5 What's the latest version?

As of this writing, the latest version is 2.0, which represents a stable version for widespread use and development.

### 1.6 Can I use Open Babel code in a personal project?

One common misconception about the GNU GPL license for Open Babel
is that it requires users to release any code that uses the Open Babel
library. This is completely untrue. There are no restrictions on use
of Open Babel code for personal projects, regardless of where you work (academia, industry, ... wherever).

**However**, if you intend on releasing a software
package that uses Open Babel code, the GPL requires that your package
be released under the GNU GPL license. The distinction is between
**use** and **distribution**. See section 3 of this FAQ
for more on the licensing issues and why you might want to contribute.

## Features, Formats, Roadmap:

### 2.1 Why don't you support file format X?

The file formats currently supported are some of the more common
file formats and, admittedly, those we use in our work. If you'd like
to see other file formats added, we need one of:

- documentation on the file format
- working code to read the file format or translate it
- example files in the new file format and in some other format

The latter obviously is the easiest with text file formats. Binary
files take some time to reverse engineer without documentation or
working code. Also consider pointing developers to this FAQ and the
"What's in it for me?" section.

### 2.2 What doesn't Open Babel support yet?

Lots of things. See the proposed
roadmap for examples of things we'd like to see in future
versions.

### 2.3 What sorts of features will be added in the future?

It's an open project, so if features are suggested or donated,
they'll be considered as much as anything else on the drawing board.
Some things are pretty clear from the roadmap.

## What's in it for me to contribute?

### 3.1 What's in it for my chemistry software company?

If your product is closed-source or otherwise incompatible with
the GPL, you cannot link directly to the code library. You can, however,
distribute Open Babel in unmodified form with your products to use the
command-line interface. This is fairly easy because the Open Babel
babel program allow reading from the standard input and writing to the
standard output (functioning as a POSIX pipe).

If you decide to distribute binaries, you
should either offer users the source if they want, or point them to
the Open Babel website. Note that if you modify the source, you
obviously can't point back to the Open Babel website -- the GPL
requires that you distribute the changed source. (Or you can convince
us to incorporate the changes and point back to us.)

What's not to like with this deal? You can have Open Babel
translate foreign file formats for you and can point users at the
website for distribution. You don't need to write tons of code for
all these formats and bug reports can be passed back to us.

Of course, there's one catch. You'll most likely need to add
feature-rich support for *your* file formats. So if you contribute a
small amount of code under the GPL to read/write your files,
everything else is handled by Open Babel.

It's a win-win for everyone. The community benefits by having
feature-rich translation code and open file formats. Your company and
its programs benefit by the ability to read just about every format
imaginable. Users benefit by using the programs they need for the
tasks they need.

### 3.2 What's in it for me as an academic?

If you're an academic developer, you certainly should read the
previous answer too. It takes little work on your part to interface
with Open Babel and you get a lot in return.

But even if you're just an academic user, there's a lot of reasons
to contribute. Most of us deal with a variety of file formats in our
work. So it's useful to translate these cleanly. If a format isn't
currently supported by Open Babel, see question 2.1 above. If you
find bugs please report them. Since it's open source, you can patch
the code yourself, recompile and have the problem fixed very quickly.

If you're inclined to write code, the GPL is an excellent option
for the academic. You're the original copyright holder, so you can do
whatever you want with the code, in addition to selling it. But if
you've also licensed it under the GPL, no one can distribute it
proprietarily (i.e., closed-source) without your agreement.
Fellow acadmics can use it
directly, learn from it, improve it and contribute back to you. Isn't
that why many of us went into science?

Once licensed under the GPL, the code **must** remain free to
interested parties. If someone modifies it, that code must still
remain under the GPL, free for all.

### 3.3 What's in it for an open-source software project?

Certainly the answers for closed-source software and academics
also apply for you. Beyond that, if your code is compatible with the
GPL, you can directly use Open Babel and all of the API. This is
already happening with the Ghemical molecular editor, available under
the GPL and many others There's a lot of
code in Open Babel beyond file translation and more to come. Why
reinvent the wheel?

### 3.4 Why is this covered under the GPL instead of license X?

The short answer is that OpenEye Scientific Software employs Matt
Stahl, one of the authors of the original Babel. They released a
library called OELib under the GPL that did many things that Babel
did. Later they decided to release the next version of OELib as a
closed-source project--their choice for their code. We took the
version of OELib still under GPL and went from there.

If you'd like to see Open Babel licensed differently, we'd suggest
asking OpenEye if they'd consider releasing the old code under a new
license, e.g. the LGPL. At that point, we'd consider whether Open
Babel should be relicensed or not. Obviously all copyright holders
must agree to the new license.

It's worth noting that since OpenEye is developing a
closed-source library called OEChem
and implies one reason for purchase is in closed-source development
products. So we think it's highly unlikely that OpenEye would allow
Open Babel to become a competitor by relicensing under the LGPL.

### Where can I read more about the GNU GPL?

The Free Software Foundation maintains a FAQ list about the
GNU GPL. The FAQ attempts to
address common questions in an easy-to-read (i.e., not in legal
language) form.
