# Supplementary material for: Incorporation of Noncanonical Amino Acids into Rosetta and Use in Computational Protein-Peptide Interface Design
Source: PLoS One. 2012 Mar 14;7(3):e32637. doi: 10.1371/journal.pone.0032637 (PMC3303795; doi:10.1371/journal.pone.0032637)
Supplement: Supporting Information S2 — “Protocol capture” that includes descriptions, instructions, command lines and processing scripts to reproduce the NCAA parameter files, explicit unfolded state energies, and rotamer libraries. Additionally the protocol capture includes descriptions, instructions, command lines and processing scripts used to redesign the NCAA peptides. (GZ) [file pone.0032637.s002.gz › using_ncaas_protein_peptide_interface_design/HowToMakeResidueTypeParamFiles/openbabel/openbabel-2.2.0.tar.gz!/openbabel-2.2.0/doc/obchiral.html]

obchiral

Table of Contents

## Name

**obchiral** -- print molecular chirality information

## **Synopsis**

**obchiral** *filename*

## **Description**

The obchiral program is a tool to print the chirality information for all
molecules in a file. It also serves as example code for using the Open
Babel library (libopenbabel).

## **Examples**

```
obchiral pyridines.sdf
```

## **See** **Also**

babel(1)
, obfit(1)
, obgrep(1)
, obprop(1)
, obrotate(1)
.

The web pages for Open Babel can be found at: <**http://openbabel.org/**>

## **Authors**

The obchiral program was written by Nick England

Open Babel is developed by a cast of many, including currrent maintainers
Geoff Hutchison, Chris Morley, Michael Banck, and innumerable others who
have contributed fixes and additions. For more contributors to Open
Babel, see <**http://openbabel.org/wiki/THANKS**>

## **Copyright**

Copyright (C) 2005 Nick England.  
Some portions Copyright (C) 1998-2001 by OpenEye Scientific Software,
Inc.  
Some portions Copyright (C) 2001-2007 by Geoffrey R. Hutchison and other
contributors.

This program is free software; you can redistribute it and/or modify it
under the terms of the GNU General Public License as published by the
Free Software Foundation version 2 of the License.

This program is distributed in the hope that it will be useful, but
WITHOUT ANY WARRANTY; without even the implied warranty of MERCHANTABILITY
or FITNESS FOR A PARTICULAR PURPOSE. See the GNU General Public
License for more details.

---

**Table of Contents**

- Name
- Synopsis
- Description
- Examples
- See Also
- Authors
- Copyright
