# Supplementary material for: Incorporation of Noncanonical Amino Acids into Rosetta and Use in Computational Protein-Peptide Interface Design
Source: PLoS One. 2012 Mar 14;7(3):e32637. doi: 10.1371/journal.pone.0032637 (PMC3303795; doi:10.1371/journal.pone.0032637)
Supplement: Supporting Information S2 — “Protocol capture” that includes descriptions, instructions, command lines and processing scripts to reproduce the NCAA parameter files, explicit unfolded state energies, and rotamer libraries. Additionally the protocol capture includes descriptions, instructions, command lines and processing scripts used to redesign the NCAA peptides. (GZ) [file pone.0032637.s002.gz › using_ncaas_protein_peptide_interface_design/HowToMakeResidueTypeParamFiles/openbabel/openbabel-2.2.0.tar.gz!/openbabel-2.2.0/doc/obconformer.html]

obconformer

Table of Contents

## Name

**obconformer** -- generate conformer coordinates

## **Synopsis**

**obconformer** *# of conformers to test* *# of geometry
optimization steps* *filename*

## **Description**

The obconformer tool can be used as part of a conformational study
by generating random conformers using a Monte Carlo search. The best
conformer out of the batch of conformers will be output, after
taking the supplied number of geometry optimization steps. By default,
obconformer uses the MMFF94 force field.

## **Examples**

Generate the best conformer (out of 250) of baseconformer.sdf and write
it to rotamer1.sdf after 100 geometry optimization steps.  

```
obconformer 250 100 baseconformer.sdf >rotamer1.sdf
```

## **See** **Also**

babel(1)
, obenergy(1)
, obfit(1)
, obgrep(1)
, obminimize(1)
, obrotate(1)
.

The web pages for Open Babel can be found at: <**http://openbabel.org/**>

## **Authors**

Open Babel is developed by a cast of many, including currrent maintainers
Geoff Hutchison, Chris Morley, Michael Banck, and innumerable others who
have contributed fixes and additions. For more contributors to Open
Babel, see <**http://openbabel.org/wiki/THANKS**>

## **Copyright**

Copyright (C) 1998-2001 by OpenEye Scientific Software, Inc.  
Some portions Copyright (C) 2001-2008 by Geoffrey R. Hutchison and other
contributors.

This program is free software; you can redistribute it and/or modify it
under the terms of the GNU General Public License as published by the
Free Software Foundation version 2 of the License.

This program is distributed in the hope that it will be useful, but
WITHOUT ANY WARRANTY; without even the implied warranty of MERCHANTABILITY
or FITNESS FOR A PARTICULAR PURPOSE. See the GNU General Public
License for more details.

---

**Table of Contents**

- Name
- Synopsis
- Description
- Examples
- See Also
- Authors
- Copyright
