# Supplementary material for: Incorporation of Noncanonical Amino Acids into Rosetta and Use in Computational Protein-Peptide Interface Design
Source: PLoS One. 2012 Mar 14;7(3):e32637. doi: 10.1371/journal.pone.0032637 (PMC3303795; doi:10.1371/journal.pone.0032637)
Supplement: Supporting Information S2 — “Protocol capture” that includes descriptions, instructions, command lines and processing scripts to reproduce the NCAA parameter files, explicit unfolded state energies, and rotamer libraries. Additionally the protocol capture includes descriptions, instructions, command lines and processing scripts used to redesign the NCAA peptides. (GZ) [file pone.0032637.s002.gz › using_ncaas_protein_peptide_interface_design/HowToMakeResidueTypeParamFiles/openbabel/openbabel-2.2.0.tar.gz!/openbabel-2.2.0/doc/obenergy.html]

obenergy

Table of Contents

## Name

**obenergy** -- calculate the energy for a molecule

## **Synopsis**

**obenergy** [*OPTIONS*] *filename*

## **Description**

The obenergy tool can be used to calculate the energy for molecules
inside (multi-)molecule files (e.g., MOL2, etc.)

## **Options**

If no filename is given, obenergy will give all options including the
available forcefields.

**-v**
:   Verbose: print out all individual energy interactions

**-ff** *forcefield*
:   Select the forcefield

## **Examples**

View the possible options, including available forcefields:  

```
obenergy
```

Calculate the energy for the molecule(s) in file test.mol2:  

```
obenergy test.mol2
```

Calculate the energy for the molecule(s) in file test.mol2 using the
Ghemical forcefield:  

```
obenergy -ff Ghemical test.mol2
```

Calculate the energy for the molecule(s) in file test.mol2 and print out
all individual energy interactions:  

```
obenergy -v test.mol2
```

## **See** **Also**

babel(1)
, obminimize(1)
, obrotamer(1)
.

The web pages for Open Babel can be found at: <**http://openbabel.org/**>

The web pages for Open Babel Molecular Mechanics can be found at:
<**http://openbabel.org/wiki/Molecular\_mechanics**>

## **Authors**

The obenergy program was contributed by Tim Vandermeersch.

Open Babel is developed by a cast of many, including currrent maintainers
Geoff Hutchison, Chris Morley, Michael Banck, and innumerable others who
have contributed fixes and additions. For more contributors to Open
Babel, see <**http://openbabel.org/wiki/THANKS**>

## **Copyright**

Copyright (C) 2007 by Tim Vandermeersch.

This program is free software; you can redistribute it and/or modify it
under the terms of the GNU General Public License as published by the
Free Software Foundation version 2 of the License.

This program is distributed in the hope that it will be useful, but
WITHOUT ANY WARRANTY; without even the implied warranty of MERCHANTABILITY
or FITNESS FOR A PARTICULAR PURPOSE. See the GNU General Public
License for more details.

---

**Table of Contents**

- Name
- Synopsis
- Description
- Options
- Examples
- See Also
- Authors
- Copyright
