# Supplementary material for: Incorporation of Noncanonical Amino Acids into Rosetta and Use in Computational Protein-Peptide Interface Design
Source: PLoS One. 2012 Mar 14;7(3):e32637. doi: 10.1371/journal.pone.0032637 (PMC3303795; doi:10.1371/journal.pone.0032637)
Supplement: Supporting Information S2 — “Protocol capture” that includes descriptions, instructions, command lines and processing scripts to reproduce the NCAA parameter files, explicit unfolded state energies, and rotamer libraries. Additionally the protocol capture includes descriptions, instructions, command lines and processing scripts used to redesign the NCAA peptides. (GZ) [file pone.0032637.s002.gz › using_ncaas_protein_peptide_interface_design/HowToMakeResidueTypeParamFiles/openbabel/openbabel-2.2.0.tar.gz!/openbabel-2.2.0/doc/obfit.html]

obfit

Table of Contents

## Name

**obfit** -- superimpose two molecules based on a pattern

## **Synopsis**

**obfit** *SMARTS-pattern* *fixed-file* *outfile*

## **Description**

Superimpose two molecules using a quaternion fit. The atoms used to fit
the two molecules are defined by the SMARTS pattern given by the user. It
is useful to align congeneric series of molecules on a common structural
scaffold for 3D-QSAR studies. It can also be useful for displaying the
results of conformational generation.

Any molecules matching the supplied SMARTS pattern will be rotated and
translated to provide the smallest possible RMSD between the matching
regions. If a molecule does not match the SMARTS pattern, it will be output
with no transformation.

## **Examples**

Align all the molecules in ‘testmv.sdf’ on a single molecule of
‘testref.sdf’ by superimposing them on its N-methylpiperidyl portion (and outputting a new SD file to the standard output):

```
obfit '[nh]1c2c(=O)n(C)c(=O)n(C)c2cc1' testref.sdf testmv.sdf
```

## **See** **Also**

babel(1)
, obchiral(1)
, obfit(1)
, obgrep(1)
, obrotate(1)
.

The web pages for Open Babel can be found at: <**http://openbabel.org/**>

A guide for constructing SMARTS patterns can be found at:
<**http://www.daylight.com/dayhtml/doc/theory/theory.smarts.html**>

## **Authors**

The obfit program was contributed by Fabien Fontaine

Open Babel is developed by a cast of many, including currrent maintainers
Geoff Hutchison, Chris Morley, Michael Banck, and innumerable others who
have contributed fixes and additions. For more contributors to Open
Babel, see <**http://openbabel.org/wiki/THANKS**>

## **Copyright**

Copyright (C) 1998-2001 by OpenEye Scientific Software, Inc.  
Some portions Copyright (C) 2001-2007 by Geoffrey R. Hutchison and other
contributors.

This program is free software; you can redistribute it and/or modify it
under the terms of the GNU General Public License as published by the
Free Software Foundation version 2 of the License.

This program is distributed in the hope that it will be useful, but
WITHOUT ANY WARRANTY; without even the implied warranty of MERCHANTABILITY
or FITNESS FOR A PARTICULAR PURPOSE. See the GNU General Public
License for more details.

---

**Table of Contents**

- Name
- Synopsis
- Description
- Examples
- See Also
- Authors
- Copyright
