# Supplementary material for: Incorporation of Noncanonical Amino Acids into Rosetta and Use in Computational Protein-Peptide Interface Design
Source: PLoS One. 2012 Mar 14;7(3):e32637. doi: 10.1371/journal.pone.0032637 (PMC3303795; doi:10.1371/journal.pone.0032637)
Supplement: Supporting Information S2 — “Protocol capture” that includes descriptions, instructions, command lines and processing scripts to reproduce the NCAA parameter files, explicit unfolded state energies, and rotamer libraries. Additionally the protocol capture includes descriptions, instructions, command lines and processing scripts used to redesign the NCAA peptides. (GZ) [file pone.0032637.s002.gz › using_ncaas_protein_peptide_interface_design/HowToMakeResidueTypeParamFiles/openbabel/openbabel-2.2.0.tar.gz!/openbabel-2.2.0/doc/obgen.html]

obgen

Table of Contents

## Name

**obgen** -- generate 3D coordinates for a molecule

## **Synopsis**

**obgen** [*OPTIONS*] *filename*

## **Description**

The obgen tool will generate 3D coordinates for molecules in a file
(e.g. multi-molecule SMILES files). The resulting structure will be
optimized using the given forcefield and checked for the
lowest-energy conformer using a Monte Carlo search.
Output will be sent to standard output in the SDF file format.

## **Options**

If no filename is given, obminimize will give all options including the
available forcefields.

**-ff** *forcefield*
:   Select the forcefield

## **Examples**

View the possible options, including available forcefields:  

```
obgen
```

Generate 3D coordinates for the molecule(s) in file test.smi:  

```
obgen test.smi
```

Generate 3D coordinates for the molecule(s) in file test.smi using the
UFF forcefield:  

```
obgen -ff UFF test.smi
```

## **See** **Also**

babel(1)
, obminimize(1)
, obconformer(1)
.

The web pages for Open Babel can be found at: <**http://openbabel.org/**>

The web pages for Open Babel Molecular Mechanics can be found at:
<**http://openbabel.org/wiki/Molecular\_mechanics**>

## **Authors**

The obgen program was contributed by Tim Vandermeersch.

Open Babel is developed by a cast of many, including currrent maintainers
Geoff Hutchison, Chris Morley, Michael Banck, and innumerable others who
have contributed fixes and additions. For more contributors to Open
Babel, see <**http://openbabel.org/wiki/THANKS**>

## **Copyright**

Copyright (C) 2007-2008 by Tim Vandermeersch.

This program is free software; you can redistribute it and/or modify it
under the terms of the GNU General Public License as published by the
Free Software Foundation version 2 of the License.

This program is distributed in the hope that it will be useful, but
WITHOUT ANY WARRANTY; without even the implied warranty of MERCHANTABILITY
or FITNESS FOR A PARTICULAR PURPOSE. See the GNU General Public
License for more details.

---

**Table of Contents**

- Name
- Synopsis
- Description
- Options
- Examples
- See Also
- Authors
- Copyright
