# Supplementary material for: Incorporation of Noncanonical Amino Acids into Rosetta and Use in Computational Protein-Peptide Interface Design
Source: PLoS One. 2012 Mar 14;7(3):e32637. doi: 10.1371/journal.pone.0032637 (PMC3303795; doi:10.1371/journal.pone.0032637)
Supplement: Supporting Information S2 — “Protocol capture” that includes descriptions, instructions, command lines and processing scripts to reproduce the NCAA parameter files, explicit unfolded state energies, and rotamer libraries. Additionally the protocol capture includes descriptions, instructions, command lines and processing scripts used to redesign the NCAA peptides. (GZ) [file pone.0032637.s002.gz › using_ncaas_protein_peptide_interface_design/HowToMakeResidueTypeParamFiles/openbabel/openbabel-2.2.0.tar.gz!/openbabel-2.2.0/doc/obgrep.html]

obgrep

Table of Contents

## Name

**obgrep** -- an advanced molecular search program using SMARTS

## **Synopsis**

**obgrep** [*OPTIONS*] *‘SMARTS-pattern’* *filename*

## **Description**

The obgrep tool can be used to search for molecules inside multi-molecule
database files (e.g., SMILES, SDF, etc.) or across multiple files.

## **Options**

If only a filename is given, obgrep will attempt to guess the file type
from the filename extension.

**-c**
:   Print the number of matches

**-f**
:   Full match, print matching-molecules only when the number of heavy
    atoms is also equal to the number of atoms in the SMARTS pattern

**-i** *format*
:   Specifies input and output format, see babel(1)
    for available formats

**-n**
:   Only print the name of the molecules

**-t** *#*
:   Print a molecule only if the pattern occurs # times inside the molecule

**-v**
:   Invert the matching, print non-matching molecules

## **Examples**

Note that in all examples, the SMARTS pattern is enclosed in single
quotes '...' to ensure it is not changed by the shell.

Print all the molecules with a methylamine group:  

```
obgrep 'CN' database.smi
```

Print all the molecules without a methylamine group:  

```
obgrep -v 'CN' database.smi
```

Print the number of molecules without a methylamine group:  

```
obgrep -v -c 'CN' database.smi
```

Print methylamine (if it exists in the file):  

```
obgrep -f 'CN' database.smi
```

Print methylamine and/or methanol (if they exist):  

```
obgrep -f 'C[N,O]' database.smi
```

Print all molecules with aromatic carbon in all SMILES files in the
directory (i.e., \*.smi):  

```
obgrep 'c' *.smi
```

## **See** **Also**

babel(1)
, obfit(1)
, obrotate(1)
.

The web pages for Open Babel can be found at: <**http://openbabel.org/**>

A guide for constructing SMARTS patterns can be found at:
<**http://www.daylight.com/dayhtml/doc/theory/theory.smarts.html**>

## **Authors**

The obgrep program was contributed by Fabien Fontaine

Open Babel is developed by a cast of many, including currrent maintainers
Geoff Hutchison, Chris Morley, Michael Banck, and innumerable others who
have contributed fixes and additions. For more contributors to Open
Babel, see <**http://openbabel.org/wiki/THANKS**>

## **Copyright**

Copyright (C) 1998-2001 by OpenEye Scientific Software, Inc.  
Some portions Copyright (C) 2001-2007 by Geoffrey R. Hutchison and other
contributors.

This program is free software; you can redistribute it and/or modify it
under the terms of the GNU General Public License as published by the
Free Software Foundation version 2 of the License.

This program is distributed in the hope that it will be useful, but
WITHOUT ANY WARRANTY; without even the implied warranty of MERCHANTABILITY
or FITNESS FOR A PARTICULAR PURPOSE. See the GNU General Public
License for more details.

---

**Table of Contents**

- Name
- Synopsis
- Description
- Options
- Examples
- See Also
- Authors
- Copyright
