# Supplementary material for: Incorporation of Noncanonical Amino Acids into Rosetta and Use in Computational Protein-Peptide Interface Design
Source: PLoS One. 2012 Mar 14;7(3):e32637. doi: 10.1371/journal.pone.0032637 (PMC3303795; doi:10.1371/journal.pone.0032637)
Supplement: Supporting Information S2 — “Protocol capture” that includes descriptions, instructions, command lines and processing scripts to reproduce the NCAA parameter files, explicit unfolded state energies, and rotamer libraries. Additionally the protocol capture includes descriptions, instructions, command lines and processing scripts used to redesign the NCAA peptides. (GZ) [file pone.0032637.s002.gz › using_ncaas_protein_peptide_interface_design/HowToMakeResidueTypeParamFiles/openbabel/openbabel-2.2.0.tar.gz!/openbabel-2.2.0/doc/obminimize.html]

obminimize

Table of Contents

## Name

**obminimize** -- optimize the geometry, minimize the energy for a molecule

## **Synopsis**

**obminimize** [*OPTIONS*] *filename*

## **Description**

The obminimize tool can be used to minimize the energy for molecules
inside (multi-)molecule files (e.g., MOL2, etc.)

## **Options**

If no filename is given, obminimize will give all options including the
available forcefields.

**-n** *steps*
:   Specify the maximum number of steps (default=2500)

**-cg**
:   Use conjugate gradients algorithm (default)

**-sd**
:   Use steepest descent algorithm

**-c** *criteria*
:   Set convergence criteria (default=1e-6)

**-ff** *forcefield*
:   Select the forcefield

## **Examples**

View the possible options, including available forcefields:  

```
obminimize
```

Minimize the energy for the molecule(s) in file test.mol2:  

```
obminimize test.mol2
```

Minimize the energy for the molecule(s) in file test.mol2 using the Ghemical
forcefield:  

```
obminimize -ff Ghemical test.mol2
```

Minimize the energy for the molecule(s) in file test.mol2 by taking at
most 300 geometry optimization steps  

```
obminimize -n 300 test.mol2
```

Minimize the energy for the molecule(s) in file test.mol2 using the
steepest descent algorithm and convergence criteria 1e-5:  

```
obminimize -sd -c 1e-5 test.mol2
```

## **See** **Also**

babel(1)
, obenergy(1)
, obrotamer(1)
.

The web pages for Open Babel can be found at: <**http://openbabel.org/**>

The web pages for Open Babel Molecular Mechanics can be found at:
<**http://openbabel.org/wiki/Molecular\_mechanics**>

## **Authors**

The minimize program was contributed by Tim Vandermeersch.

Open Babel is developed by a cast of many, including currrent maintainers
Geoff Hutchison, Chris Morley, Michael Banck, and innumerable others who
have contributed fixes and additions. For more contributors to Open
Babel, see <**http://openbabel.org/wiki/THANKS**>

## **Copyright**

Copyright (C) 2007 by Tim Vandermeersch.

This program is free software; you can redistribute it and/or modify it
under the terms of the GNU General Public License as published by the
Free Software Foundation version 2 of the License.

This program is distributed in the hope that it will be useful, but
WITHOUT ANY WARRANTY; without even the implied warranty of MERCHANTABILITY
or FITNESS FOR A PARTICULAR PURPOSE. See the GNU General Public
License for more details.

---

**Table of Contents**

- Name
- Synopsis
- Description
- Options
- Examples
- See Also
- Authors
- Copyright
