# Supplementary material for: Incorporation of Noncanonical Amino Acids into Rosetta and Use in Computational Protein-Peptide Interface Design
Source: PLoS One. 2012 Mar 14;7(3):e32637. doi: 10.1371/journal.pone.0032637 (PMC3303795; doi:10.1371/journal.pone.0032637)
Supplement: Supporting Information S2 — “Protocol capture” that includes descriptions, instructions, command lines and processing scripts to reproduce the NCAA parameter files, explicit unfolded state energies, and rotamer libraries. Additionally the protocol capture includes descriptions, instructions, command lines and processing scripts used to redesign the NCAA peptides. (GZ) [file pone.0032637.s002.gz › using_ncaas_protein_peptide_interface_design/HowToMakeResidueTypeParamFiles/openbabel/openbabel-2.2.0.tar.gz!/openbabel-2.2.0/doc/obprobe.html]

obprobe

Table of Contents

## Name

**obprobe** -- create electrostatic probe grid

## **Synopsis**

**obprop** *[options]* *type* *pcharge* *filename*

## **Description**

The obprobe tool creates a grid around a molecule, placing a probe
atom with a specified atom type and partial charge at each point to
calculate the MMFF94 energy. This can be used for docking experiments
to test hydrogen-bond affinity, electrostatic potential, etc.
Output is sent to standard output using the Gaussian Cube format.

## **Options**

If no filename is given, obprobe will give all options including the
example probes.

**-s** *step size*
:   Set the resolution of the grid (stepsize)

**-p** *padding*
:   Set the padding -- extra distance on each side of the box formed by
    the molecule.

*type*
:   The MMFF94 atom type code of the probe atom.

*pchg*
:   The partial charge on the probe atom.

## **Examples**

Probe the file pyridines.sdf using a carbonyl oxygen -- a hydrogen
bond acceptor with partial charge -0.57:

```
obprobe 7 -0.57 pyridines.sdf
```

Probe the file pyridines.sdf using a phenyl carbon atom -- a
hydrophobic atom with no partial charge:

```
obprobe 37 0.0 pyridines.sdf
```

## **See** **Also**

babel(1).

The web pages for Open Babel can be found at: <**http://openbabel.org/**>

## **Authors**

The obprobe program was contributed by Tim Vandermeersch

Open Babel is developed by a cast of many, including currrent maintainers
Geoff Hutchison, Chris Morley, Michael Banck, and innumerable others who
have contributed fixes and additions. For more contributors to Open
Babel, see <**http://openbabel.org/wiki/THANKS**>

## **Copyright**

Copyright (C) 2006-2008 by Tim Vandermeersch  
Some portions Copyright (C) 2004-2008 by Geoffrey R. Hutchison and other
contributors.

This program is free software; you can redistribute it and/or modify it
under the terms of the GNU General Public License as published by the
Free Software Foundation version 2 of the License.

This program is distributed in the hope that it will be useful, but
WITHOUT ANY WARRANTY; without even the implied warranty of MERCHANTABILITY
or FITNESS FOR A PARTICULAR PURPOSE. See the GNU General Public
License for more details.

---

**Table of Contents**

- Name
- Synopsis
- Description
- Options
- Examples
- See Also
- Authors
- Copyright
