# Supplementary material for: Incorporation of Noncanonical Amino Acids into Rosetta and Use in Computational Protein-Peptide Interface Design
Source: PLoS One. 2012 Mar 14;7(3):e32637. doi: 10.1371/journal.pone.0032637 (PMC3303795; doi:10.1371/journal.pone.0032637)
Supplement: Supporting Information S2 — “Protocol capture” that includes descriptions, instructions, command lines and processing scripts to reproduce the NCAA parameter files, explicit unfolded state energies, and rotamer libraries. Additionally the protocol capture includes descriptions, instructions, command lines and processing scripts used to redesign the NCAA peptides. (GZ) [file pone.0032637.s002.gz › using_ncaas_protein_peptide_interface_design/HowToMakeResidueTypeParamFiles/openbabel/openbabel-2.2.0.tar.gz!/openbabel-2.2.0/doc/obrotate.html]

obrotate

Table of Contents

## Name

**obrotate** -- batch-rotate dihedral angles matching SMARTS patterns

## **Synopsis**

**obrotate** *‘SMARTS-pattern’* *filename* *atom1* *atom2* *atom3* *atom4* *angle*

## **Description**

The obrotate program rotates the torsional (dihedral) angle of a specified
bond in molecules to that defined by the user. In other words, it
does the same as a user setting an angle in a molecular modelling package,
but much faster and in batch mode (i.e. across multiple molecules in
a file).

The four atom IDs required are indexes into the SMARTS pattern, which
starts at atom 0 (zero). The angle supplied is in degrees. The two atoms
used to set the dihedral angle <atom1> and <atom4> do not need to be connected
to the atoms of the bond <atom2> and <atom3> in any way.

The order of the atoms matters -- the portion of the molecule attached to
<atom1> and <atom2> remain fixed, but the portion bonded to <atom3> and &
<atom4> moves.

## **Examples**

Let’s say that you want to define the conformation of a large number of
molecules with a pyridyl scaffold and substituted with an aliphatic chain
at the 3-position, for example for docking or 3D-QSAR purposes.

To set the value of the first dihedral angle to 90 degrees:  

```
obrotate ‘c1ccncc1CCC’ pyridines.sdf 5 6 7 8 90
```

Here 6 and 7 define the bond to rotate in the SMARTS patter, i.e., c1-C
and atoms 5 and 8 define the particular dihedral angle to rotate.

Since the atoms to define the dihedral do not need to be directly connected,
the nitrogen in the pyridine can be used:  

```
obrotate ‘c1ccncc1CCC’ pyridines.sdf 4 6 7 8 90
```

Keep the pyridyl ring fixed and moves the aliphatic chain:  

```
obrotate ‘c1ccncc1CCC’ pyridines.sdf 5 6 7 8 90
```

Keep the aliphatic chain fixed and move the pyridyl ring:  

```
obrotate ‘c1ccncc1CCC’ pyridines.sdf 8 7 6 5 90
```

## **See** **Also**

babel(1)
, obchiral(1)
, obfit(1)
, obgrep(1)
, obprop(1)
, obrotate(1)
.

The web pages for Open Babel can be found at: <**http://openbabel.org/**>

A guide for constructing SMARTS patterns can be found at:
<**http://www.daylight.com/dayhtml/doc/theory/theory.smarts.html**>

## **Authors**

The obgrep program was contributed by Fabien Fontaine

Open Babel is developed by a cast of many, including currrent maintainers
Geoff Hutchison, Chris Morley, Michael Banck, and innumerable others who
have contributed fixes and additions. For more contributors to Open
Babel, see <**http://openbabel.org/wiki/THANKS**>

## **Copyright**

Copyright (C) 1998-2001 by OpenEye Scientific Software, Inc.  
Some portions Copyright (C) 2001-2007 by Geoffrey R. Hutchison and other
contributors.

This program is free software; you can redistribute it and/or modify it
under the terms of the GNU General Public License as published by the
Free Software Foundation version 2 of the License.

This program is distributed in the hope that it will be useful, but
WITHOUT ANY WARRANTY; without even the implied warranty of MERCHANTABILITY
or FITNESS FOR A PARTICULAR PURPOSE. See the GNU General Public
License for more details.

---

**Table of Contents**

- Name
- Synopsis
- Description
- Examples
- See Also
- Authors
- Copyright
