# Supplementary material for: Incorporation of Noncanonical Amino Acids into Rosetta and Use in Computational Protein-Peptide Interface Design
Source: PLoS One. 2012 Mar 14;7(3):e32637. doi: 10.1371/journal.pone.0032637 (PMC3303795; doi:10.1371/journal.pone.0032637)
Supplement: Supporting Information S2 — “Protocol capture” that includes descriptions, instructions, command lines and processing scripts to reproduce the NCAA parameter files, explicit unfolded state energies, and rotamer libraries. Additionally the protocol capture includes descriptions, instructions, command lines and processing scripts used to redesign the NCAA peptides. (GZ) [file pone.0032637.s002.gz › using_ncaas_protein_peptide_interface_design/HowToMakeResidueTypeParamFiles/openbabel/openbabel-2.2.0.tar.gz!/openbabel-2.2.0/scripts/python/pybelapi.html]

Python: module pybel

|  |  |
| --- | --- |
| **pybel** | index d:\tools\cinfony\trunk\cinfony\pybel.py |

**Modules**

|  |  |  |  |
| --- | --- | --- | --- |
| Image  math | oasa  openbabel | os  ImageTk | tempfile  Tkinter |

**Classes**

\_\_builtin\_\_.object
:   Atom

    Fingerprint

    Molecule

    MoleculeData

    Outputfile

    Smarts

|  |  |  |
| --- | --- | --- |
| class **Atom**(\_\_builtin\_\_.object) | | |
|  | Represent a Pybel atom.     Required parameter:     OBAtom -- an Open Babel OBAtom        Attributes:     atomicmass, atomicnum, cidx, coords, coordidx, exactmass,     formalcharge, heavyvalence, heterovalence, hyb, idx,     implicitvalence, index, isotope, partialcharge, spin, type,     valence, vector.     (refer to the Open Babel library documentation for more info).     The original Open Babel atom can be accessed using the attribute:     OBAtom | |
|  |  | Methods defined here:  **\_\_getattr\_\_**(self, attr)  **\_\_init\_\_**(self, OBAtom)  **\_\_str\_\_**(self)  Create a string representation of the atom.     >>> a = Atom()  >>> print a  Atom: 0 (0.0, 0.0, 0.0)   ---  Data descriptors defined here:  **\_\_dict\_\_**  dictionary for instance variables (if defined)  **\_\_weakref\_\_**  list of weak references to the object (if defined) |

|  |  |  |
| --- | --- | --- |
| class **Fingerprint**(\_\_builtin\_\_.object) | | |
|  | A Molecular Fingerprint.     Required parameters:     obFingerprint -- a vector calculated by OBFingerprint.FindFingerprint()     Attributes:     fp -- the original obFingerprint     bits -- a list of bits set in the Fingerprint     Methods:     The "\|" operator can be used to calculate the Tanimoto coeff. For example,     given two Fingerprints 'a', and 'b', the Tanimoto coefficient is given by:        tanimoto = a \| b | |
|  |  | Methods defined here:  **\_\_getattr\_\_**(self, attr)  **\_\_init\_\_**(self, obFingerprint)  **\_\_or\_\_**(self, other)  **\_\_str\_\_**(self)   ---  Data descriptors defined here:  **\_\_dict\_\_**  dictionary for instance variables (if defined)  **\_\_weakref\_\_**  list of weak references to the object (if defined) |

|  |  |  |
| --- | --- | --- |
| class **Molecule**(\_\_builtin\_\_.object) | | |
|  | Represent a Pybel molecule.     Required parameter:     OBMol -- an Open Babel OBMol     or     Molecule -- any type of cinfony Molecule (e.g. one from cinfony.rdkit)     If a cinfony Molecule is provided it will be converted into a pybel Molecule.            Attributes:     atoms, charge, data, dim, energy, exactmass, flags, formula,      mod, molwt, spin, sssr, title, unitcell.  (refer to the Open Babel library documentation for more info).     Methods:     addh(), calcfp(), calcdesc(), draw(), localopt(), make3D(), removeh(), write()       The original Open Babel molecule can be accessed using the attribute:     OBMol | |
|  |  | Methods defined here:  **\_\_getattr\_\_**(self, attr)  Return the value of an attribute     Note: The values are calculated on-the-fly. You may want to store the value in  a variable if you repeatedly access the same attribute.  **\_\_init\_\_**(self, OBMol)  **\_\_iter\_\_**(self)  Iterate over the Atoms of the Molecule.     This allows constructions such as the following:     for atom in mymol:         print atom  **\_\_str\_\_**(self)  **addh**(self)  Add hydrogens.  **calcdesc**(self, descnames=[])  Calculate descriptor values.     Optional parameter:     descnames -- a list of names of descriptors     If descnames is not specified, the full list of Open Babel  descriptors is calculated: LogP, PSA and MR.  **calcfp**(self, fptype='FP2')  Calculate a molecular fingerprint.     Optional parameters:     fptype -- the name of the Open Babel fingerprint type.     If fptype is not specified, the FP2 fingerprint type  is used. See the Open Babel library documentation for more  details.  **draw**(self, show=True, filename=None, update=False, usecoords=False)  Create a 2D depiction of the molecule.     Optional parameters:    show -- display on screen (default is True)    filename -- write to file (default is None)    update -- update the coordinates of the atoms to those              determined by the structure diagram generator              (default is False)    usecoords -- don't calculate 2D coordinates, just use                 the current coordinates (default is False)     OASA is used for 2D coordinate generation and depiction. Tkinter and  Python Imaging Library are required for image display.  **localopt**(self, forcefield='MMFF94', steps=500)  Locally optimize the coordinates.     Optional parameters:     forcefield -- default is "MMFF94"     steps -- default is 500     If the molecule does not have 2D or 3D coordinates, make3D() is  called before the optimization. Note that the molecule needs  to have explicit hydrogens. If not, call addh().  **make3D**(self, forcefield='MMFF94', steps=50)  Generate 3D coordinates.     Optional parameters:     forcefield -- default is "MMFF94"     steps -- default is 50     Once coordinates are generated, hydrogens are added and a quick  local optimization is carried out with 50 steps and the  MMFF94 forcefield. Call localopt() if you want  to improve the coordinates further.  **removeh**(self)  Remove hydrogens.  **write**(self, format='SMI', filename=None, overwrite=False)  Write the molecule to a file or return a string.     Optional parameters:     format -- default is "SMI"     filename -- default is None     overwite -- default is False     If a filename is specified, the result is written to a file.  Otherwise, a string is returned containing the result.  The overwrite flag is ignored if a filename is not specified.  It controls whether to overwrite an existing file.   ---  Data descriptors defined here:  **\_\_dict\_\_**  dictionary for instance variables (if defined)  **\_\_weakref\_\_**  list of weak references to the object (if defined) |

|  |  |  |
| --- | --- | --- |
| class **MoleculeData**(\_\_builtin\_\_.object) | | |
|  | Store molecule data in a dictionary-type object     Required parameters:    obmol -- an Open Babel OBMol      Methods and accessor methods are like those of a dictionary except  that the data is retrieved on-the-fly from the underlying OBMol.     Example:  >>> mol = readfile("sdf", 'head.sdf').next()  >>> data = mol.data  >>> print data  {'Comment': 'CORINA 2.61 0041  25.10.2001', 'NSC': '1'}  >>> print len(data), data.keys(), data.has\_key("NSC")  2 ['Comment', 'NSC'] True  >>> print data['Comment']  CORINA 2.61 0041  25.10.2001  >>> data['Comment'] = 'This is a new comment'  >>> for k,v in data.iteritems():  ...    print k, "-->", v  Comment --> This is a new comment  NSC --> 1  >>> del data['NSC']  >>> print len(data), data.keys(), data.has\_key("NSC")  1 ['Comment'] False | |
|  |  | Methods defined here:  **\_\_contains\_\_**(self, key)  **\_\_delitem\_\_**(self, key)  **\_\_getitem\_\_**(self, key)  **\_\_init\_\_**(self, obmol)  **\_\_iter\_\_**(self)  **\_\_len\_\_**(self)  **\_\_repr\_\_**(self)  **\_\_setitem\_\_**(self, key, value)  **clear**(self)  **has\_key**(self, key)  **items**(self)  **iteritems**(self)  **keys**(self)  **update**(self, dictionary)  **values**(self)   ---  Data descriptors defined here:  **\_\_dict\_\_**  dictionary for instance variables (if defined)  **\_\_weakref\_\_**  list of weak references to the object (if defined) |

|  |  |  |
| --- | --- | --- |
| class **Outputfile**(\_\_builtin\_\_.object) | | |
|  | Represent a file to which \*output\* is to be sent.     Although it's possible to write a single molecule to a file by  calling the write() method of a molecule, if multiple molecules  are to be written to the same file you should use the Outputfile  class.     Required parameters:     format     filename  Optional parameters:     overwrite (default is False) -- if the output file already exists,                                     should it be overwritten?  Methods:     write(molecule), close() | |
|  |  | Methods defined here:  **\_\_init\_\_**(self, format, filename, overwrite=False)  **close**(self)  Close the Outputfile to further writing.  **write**(self, molecule)  Write a molecule to the output file.     Required parameters:     molecule   ---  Data descriptors defined here:  **\_\_dict\_\_**  dictionary for instance variables (if defined)  **\_\_weakref\_\_**  list of weak references to the object (if defined) |

|  |  |  |
| --- | --- | --- |
| class **Smarts**(\_\_builtin\_\_.object) | | |
|  | A Smarts Pattern Matcher     Required parameters:     smartspattern     Methods:     findall(molecule)     Example:  >>> mol = readstring("smi","CCN(CC)CC") # triethylamine  >>> smarts = Smarts("[#6][#6]") # Matches an ethyl group  >>> print smarts.findall(mol)   [(1, 2), (4, 5), (6, 7)]     The numbers returned are the indices (starting from 1) of the atoms  that match the SMARTS pattern. In this case, there are three matches  for each of the three ethyl groups in the molecule. | |
|  |  | Methods defined here:  **\_\_init\_\_**(self, smartspattern)  Initialise with a SMARTS pattern.  **findall**(self, molecule)  Find all matches of the SMARTS pattern to a particular molecule.     Required parameters:     molecule   ---  Data descriptors defined here:  **\_\_dict\_\_**  dictionary for instance variables (if defined)  **\_\_weakref\_\_**  list of weak references to the object (if defined) |

|  |  |  |
| --- | --- | --- |
| **Functions** | | |
|  |  | **readfile**(format, filename)  Iterate over the molecules in a file.     Required parameters:     format     filename     You can access the first molecule in a file using:      mol = readfile("smi", "myfile.smi").next()        You can make a list of the molecules in a file using:      mols = [mol for mol in readfile("smi", "myfile.smi")]        You can iterate over the molecules in a file as shown in the  following code snippet...     >>> atomtotal = 0  >>> for mol in readfile("sdf","head.sdf"):  ...     atomtotal += len(mol.atoms)  ...  >>> print atomtotal  43  **readstring**(format, string)  Read in a molecule from a string.     Required parameters:     format     string     >>> input = "C1=CC=CS1"  >>> mymol = readstring("smi",input)  >>> len(mymol.atoms)  5 |

|  |  |  |
| --- | --- | --- |
| **Data** | | |
|  |  | **descs** = ['LogP', 'MR', 'TPSA']  **forcefields** = ['UFF', 'MMFF94', 'Ghemical']  **fps** = ['FP2', 'FP3', 'FP4']  **informats** = {'acr': 'ACR format', 'alc': 'Alchemy format', 'arc': 'Accelrys/MSI Biosym/Insight II CAR format', 'bgf': 'MSI BGF format', 'box': 'Dock 3.5 Box format', 'bs': 'Ball and Stick format', 'c3d1': 'Chem3D Cartesian 1 format', 'c3d2': 'Chem3D Cartesian 2 format', 'caccrt': 'Cacao Cartesian format', 'car': 'Accelrys/MSI Biosym/Insight II CAR format', ...}  **operations** = ['Gen3D']  **outformats** = {'alc': 'Alchemy format', 'bgf': 'MSI BGF format', 'box': 'Dock 3.5 Box format', 'bs': 'Ball and Stick format', 'c3d1': 'Chem3D Cartesian 1 format', 'c3d2': 'Chem3D Cartesian 2 format', 'cac': 'CAChe MolStruct format', 'caccrt': 'Cacao Cartesian format', 'cache': 'CAChe MolStruct format', 'cacint': 'Cacao Internal format', ...} |
