# Supplementary material for: Incorporation of Noncanonical Amino Acids into Rosetta and Use in Computational Protein-Peptide Interface Design
Source: PLoS One. 2012 Mar 14;7(3):e32637. doi: 10.1371/journal.pone.0032637 (PMC3303795; doi:10.1371/journal.pone.0032637)
Supplement: Supporting Information S2 — “Protocol capture” that includes descriptions, instructions, command lines and processing scripts to reproduce the NCAA parameter files, explicit unfolded state energies, and rotamer libraries. Additionally the protocol capture includes descriptions, instructions, command lines and processing scripts used to redesign the NCAA peptides. (GZ) [file pone.0032637.s002.gz › using_ncaas_protein_peptide_interface_design/HowToMakeResidueTypeParamFiles/openbabel/openbabel-2.2.0.tar.gz!/openbabel-2.2.0/windows-vc2005/OBGUI/OpenBabelGUI.html]

OpenBabelGUI Help


## OpenBabelGUI Help

### Basic operation

OpenBabel converts chemical objects (currently molecules or reactions) from one file format to another. This interface is an alternative to a command line and has the same capabilities.

- Select the type of the type of the input file from the dropdown list.

- Click the "..." button and select the file. Its contents are displayed.

- Choose the output format and file in a similar way. You can merely display the
output without saving it by not selecting an output file or by checking "Output below only..".

- Click the "Convert" button.

The message window at the top of the right hand pane gives the number of molecules converted, and the contents of the output file are displayed.

By default, all the molecules in an input file are converted if the output format allows multiple molecules.

### Options

The options in the middle are those appropriate for the type of chemical object being converted (molecule or reaction) and the input and output formats. They are derived from the description text that is displayed with the -Hxxx option in the command line interface and with the "Format info" buttons here. You can switch off the display of any of the various types of option using the View menu, if the screen is getting too cluttered.

### Multiple input files

You can select multiple input files in the input file dialog in the normal way (using the Control key in Windows). In the input filename box, each filename is displayed relative to the path shown just above the box, which is the path of the first file. You can display any of the files by moving the highlight with Tab/Shift Tab, Page Up/Down, the mouse wheel, or by double clicking.

Selecting one or more new file names normally removes those already present, but they can instead be appended by holding the Control key down when leaving the file selection dialog.

In Windows, files can be also be dragged from Windows Explorer, with or without the Control key.

Normally each file is converted according to its extension and the input files do not have to be all the same type, but if you want to use non-standard file names set the checkbox "Use this format for all input files..."

If you want to combine multiple molecules (from one or more files) into a single molecule with disconnected parts, use option "Join all input molecules..."

### Wildcards in filenames

When an input filenames are typed in directly, any of them can contain the wildcard characters \* and ? Typing Enter will replace these by a list of the matching files. The wildcarded names can be restored by typing Enter while holding down the Shift key. The original or the expanded versions will behave the same when the "Convert" button is pressed.

By including the wildcard \* in the both the input and output filenames you can carry out batch conversion. Suppose there were files first.smi, second.smi, third.smi. Using \*.smi as the input filename and \*.mol as the output filename would produce three files first.mol, second.mol and third.mol. If the output filename was NEW\_\*.mol the output files would be NEW\_first.mol, etc.

### Local input

By checking the "Input below..." checkbox you can type the input text directly. The text box changes colour to remind you that it is this text and not the contents of any files that will be converted.

### Output file

The output file name can be fully specified with a path, but if it is not, then it is considered to be relative to the input file path.

### Using a restriced set of formats

It is probable that most of the large range of formats will usually not be required when OpenBabel is used regularly. You can restrict the choice offered in the dropdown boxes, which mkes routine selection easier. Clicking "Select set of formats" on the View menu allows the formats to be displayed to be selected. Subsequently, clicking "Use restricted set of formats" on the View menu toggles this facility on and off.

Using a restricted set overcomes an irritating bug in the Windows version. In the file Open and Save dialogs the files displayed can be filtered by the current format, All Chemical Formats, or All Files. The All Chemical Formats filter will only display the first 30 possible formats (alphabetically). The All Files will indeed display all files and the conversion processes are unaffected.

### Other features

Most of the interface parameters, such as the selected format and the window size and position are remembered between sessions.

Using the View menu, the input and output text boxes can be set not to wrap the text. At present you have to restart the program for this to take effect, but its hoped to improve this soon.

The message box at the top of the output text window receives program output on error and audit logging, and some progress reports. It can be expanded by dragging down the divider between the windows.

### Example files

There are three chemical files in the executable folder to try out:  
**serotonin.mol** which is has 3D atom coordinates;  
**oxamide.cml** which is 2D and has a large number of properties that will be seen when converting to sdf.  
**FourSmallMols.cml** which (unsurprisingly) contains four molecules with no atom coordinates and can be used to illustrate the handling of multiple molecules. Setting the output format to smi (which is easy to see), you can convert only the second and third molecules by entering 2 and 3 in the appropriate option boxes. Or convert only molecules with C-O single bonds by entering CO in the SMARTS option box.

---

Please report bugs or suggestions for improvement to the OpenBabel website.
